# Selective Substitution of 31/42–OH in Rapamycin Guided by an *in Situ* IR Technique

**DOI:** 10.3390/molecules19067770

**Published:** 2014-06-10

**Authors:** Shuang Cao, Xinbo Zhou, Yuanshuai Yang, Wu Zhong, Tiemin Sun

**Affiliations:** 1 Key Laboratory of Structure-Based Drug Design and Discovery, Shenyang Pharmaceutical University, Ministry of Education, Shenyang 110016, China; E-Mails: caoshuangknight@163.com (S.C.); yangmarshal88@163.com (Y.Y.); 2 Laboratory of Computer-Aided Drug Design & Discovery, Beijing Institute of Pharmacology and Toxicology, Beijing 100850, China; E-Mail: hapwave@163.com

**Keywords:** rapamycin derivatives, *in situ* IR, selective substitution, aqueous solubility

## Abstract

An *in situ* IR technique was applied in the selective synthesis of the key intermediate for rapamycin derivatives, which made the reaction endpoint easily defined. This technology solved a bothersome problem in the preparation of rapamycin derivatives, and based on this technique, the 31-OH and 42-OH of rapamycin were chemically modified by a series of quaternary ammonium salts to generate 11 compounds. The solubility of all these compounds was remarkably improved (25,000 times higher than that of rapamycin) and their structures were confirmed by MS, IR, 1D and 2D NMR techniques.

## 1. Introduction

**Figure 1:**
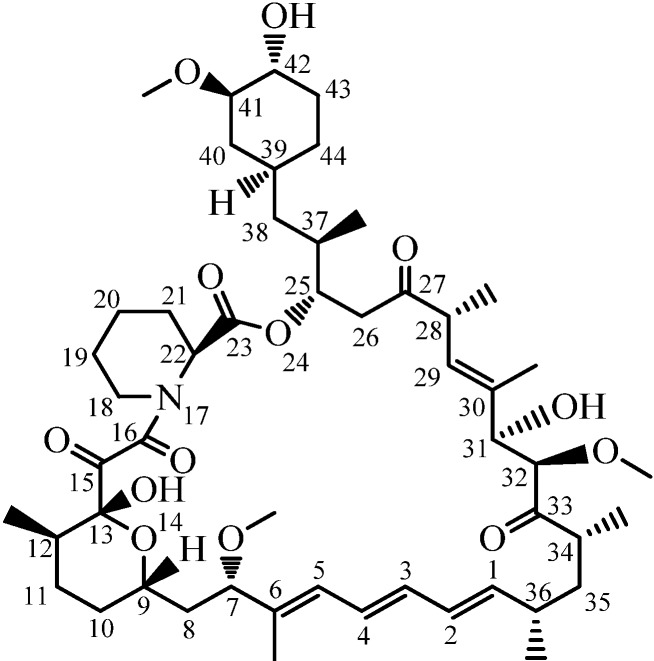
Structure of rapamycin (Rapa).

Rapamycin (Rapa, Figure 1) is a carboxylic lactone-lactam macrolide derived from the bacterium *Streptomyces hygroscopicus* [1]. It was originally used as an immunosuppressant in the treatment of organ rejection in transplant recipients, and then as an anti-cancer drug [2]. In recent years, some new applications of rapamycin and rapamycin analogues have been disclosed, such as anti-aging, anti-HIV, and so on [3]. Rapamycin has brought a new dawn to cancer treatment due to its special mechanism of inhibiting cancer cell growth via inhibition of the mammalian target of rapamycin (mTOR), which is the key factor in cell proliferation regulation [4].

Although Rapa has shown great potential in numerous ways, it also has two severe drawbacks, namely poor aqueous solubility (2.6 μg/mL) [5] and low oral bioavailability (10%) [6]. To overcome these problems, a number of Rapa derivatives have been developed, the majority of which are derived from modifications on the 42-OH of Rapa. For example, Temsirolimus (CCI-779, Wyeth, Madison, NJ, USA, 2007) [7], Everolimus (RAD001, Novartis, Basel, Switzerland, 2003), and Zotarolimus (ABT 578, Abbott, Chicago, IL, USA, 2006) [2], These successful drugs suggest that the modification on the 42-OH may be a more wise choice to optimize rapamycin, but the selective substitution of 42-OH is very difficult because the structure of Rapa is very complicated and there are three -OHs in the structure thereof.

**Scheme 1:**
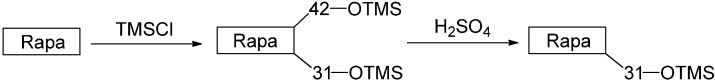
Synthesis of Rapa-31-OTMS intermediate.

The traditional avenue for selective synthesis of 42-Rapa derivatives was first reported in US Patent 6277983 [8]. The crucial point of this method is preparation of the intermediate Rapa-31-OTMS (Scheme 1), which is also the key intermediate for preparating 31-Rapa derivatives. However, in practice, we found that though the yield of this preparation could reach up to 70% (column separation), it is instable, and additionally, a lot of raw material and by-product are comingled with the end-product.

What contributes to this problem is the difficulty in defining a suitable reaction endpoint, in other words, the amount of acid and the reaction time are difficult to control just right. On one hand, it is necessary to use enough acid and the correct reaction time to convert Rapa-31,42-OTMS intoRapa-31-OTMS. On the other hand, too much of them will lead to the decomposition of the Rapa-31-OTMS into Rapa. Moreover, there exists a connection between the amount of acid and the reaction time. The amount of acid can be reduced by extending the reaction time, and *vice versa*. These problems manifest especially prominently in industrial production.

In view of the high cost of the raw material (Rapa) and the difficulty of accurate control in the traditional method, a new technology is seriously required to solve these problems. Herein, we report an application of *in situ* IR in selective synthesis of the key intermediate for Rapa derivatives. Based on this method, a series of mono-substituted Rapa derivatives with quaternary ammonium groups were obtained, which all had excellent aqueous solubility.

## 2. Results and Discussion

### 2.1. In Situ IR

**Figure 2:**
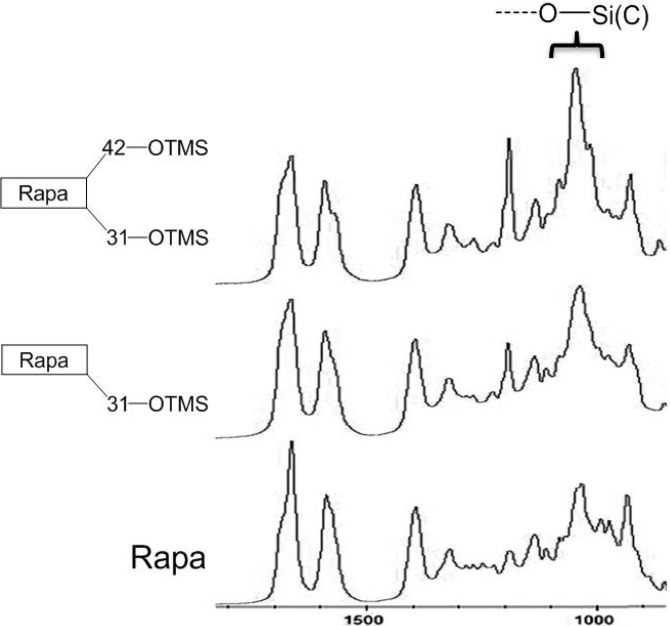
Expanded solid state FT-IR spectra for Rapa, Rapa-31-OTMS and Rapa-31,42-OTMS.

**Figure 3:**
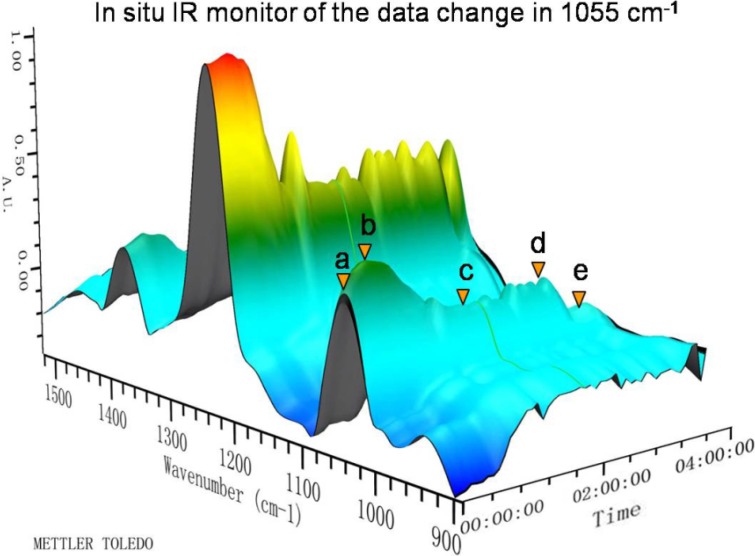
3D FT-IR spectrum fragments for the preparation of Rapa-31-OTMS from Rapa. The reaction was monitored from 0 to 230 min. (**a**) Addition of TMSCl and imidazole (00:05:00). (**b**) Addition of 0.5 N H_2_SO_4_ (00:25:00 to 03:25:00). (**a**) to (**b**) Rapa is totally converted to Rapa-31,42-OTMS. (**b**) to (**c**) Rapa-31,42-OTMS is mostly converted into Rapa-31-OTMS. (**d**) to (**e**): Rapa-31-OTMS is totally converted into Rapa.

Through analysis of a series of features in the IR spectrum of Rapa-31-OTMS (Figure 2), the intensity of the absorption peak at 1.055 cm^−1^, a characteristic peak attributed to -Si-O(C) [9,10,11,12], was selected as marker to monitor the levels of Rapa, Rapa-31,42-OTMS and Rapa-31-OTMS indirectly (Figure 3) [13]. The structure of Rapa-31,42-OTMS and Rapa-31-OTMS had been identified by ^1^H-NMR (Supplementary Material).

**Figure 4:**
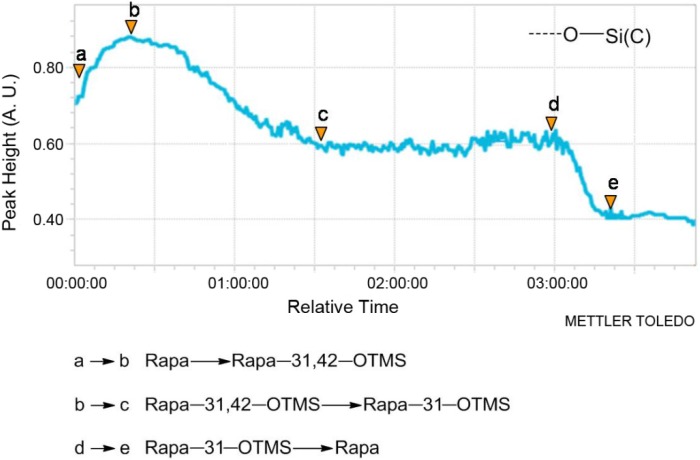
Kinetic profile (peak height *vs.* time) of the intensity of the absorption peak at 1055 cm^−1^ (the characteristic peak attributed to -Si-O(C)) for the reaction of preparation of Rapa-31-OTMS from Rapa. (**a**) Addition of TMSCl and imidazole (00:05:00). (**b**) Addition of 0.5 N H_2_SO_4_ (00:25:00 to 03:25:00).

It can be seen from the profile (Figure 3 and Figure 4) that the peak intensity at 1055 cm^−1^ increases markedly from **a** to **b**, indicating that Rapa is quickly converted to Rapa-31,42-OTMS. After adding H_2_SO_4_ at point **b**, the peak gradually decreased and then remained stable from **c** to **d**. This result indicates that Rapa-31,42-OTMS is slowly transformed into Rapa-31-OTMS and then the level of Rapa-31-OTMS remains mostly unchanged, while the sharp decrease of the absorption peak from **d** to **e** indicates that Rapa-31-OTMS begin to convert back to Rapa. The long reaction time and continuously rising acidity lead to the quick hydrolysis of Rapa-31-OTMS after the breakthrough point (**d**). From the *in situ* IR data, we concluded that the reaction endpoint lay between **c** and **d** with a stable yield of 88% (column separation). *In situ* IR has the advantages of being highly sensitive, continuous and real-time, which make it more useful than TLC and HPLC, especially for a complicated reaction. This technology could give explicit instructions for process optimization and quality control in the preparation of Rapa-31-OTMS, especially in industrial production.

### 2.2. Synthesis

After determining the preparation and control method, the technique was immediately applied in the synthesis of new Rapa derivatives. The choice of substituent groups is another key point to this subject. To avoid the toxicity and side effects produced by metabolism of substituent groups, *N*-(carboxymethyl) thiazolium (or pyridine) derivative side chains were introduced into 31-OH and 42-OH by esterification. The quaternary ammonium salt derivatives developed by our team were nontoxic and showed good water-solubility, and all of them were themselves drugs (AGEs cross-link breakers).

**Scheme 2:**
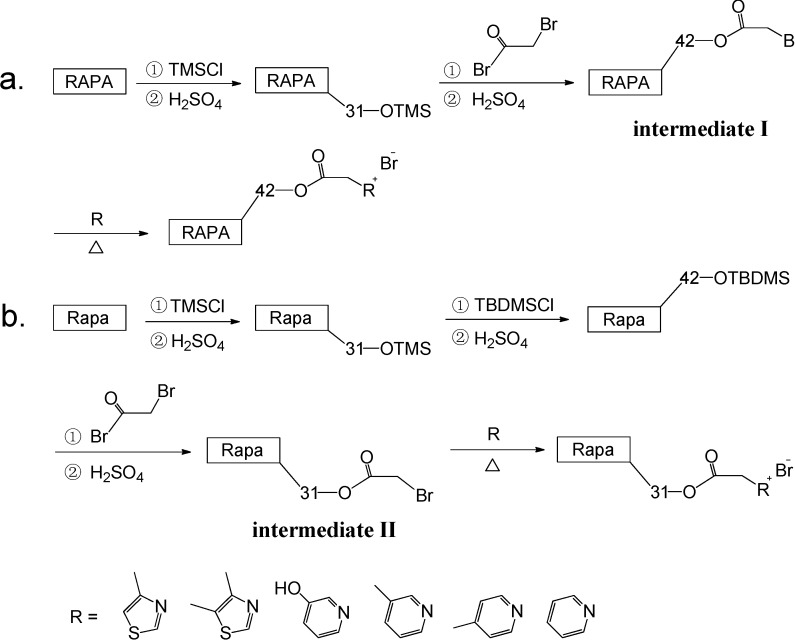
Synthesis routes of 42-Rapa derivatives (**a**) and 31-Rapaderivatives (**b**).

In the paper, the traditional synthesis method was optimized by merging two reactions into one (Scheme 2), so that the reaction steps in the preparation of 42-Rapa derivatives were decreased from 5 to 3 (overall yield 48%). The reaction merging strategy could also be implemented in the synthesis of 31-Rapa derivatives, in which the number of steps could be decreased from 7 to 4 (overall yield 15%). These selective synthesis strategies were achieved by tactfully taking advantage of the subtle differences in reactivity between the two positions, whereby that of the 42-OH is greater than the 31-OH, as well as the greater reactivity of the *tert*-butyldimethylsilyl (TBDMS) ether which is stronger than that of the trimethylsilyl (TMS) ether [14,15,16]. Smaller steric hindrance may be the main reason for the high reaction activity of the 42-OH position.

Finally, 11 compounds were produced (Table 1), which were all quaternary ammonium salts. According to the reaction point, these compounds can be classified into two series, 42-Rapa derivatives and 31-Rapa derivatives.

### 2.3. Structure

For these complicated compounds, the determination of the linkage position of the substituent groups is difficult but indispensable. Here, a method for confirming the attachment position and number of substituent groups was developed based on the corresponding NMR spectra.

**Figure 5:**
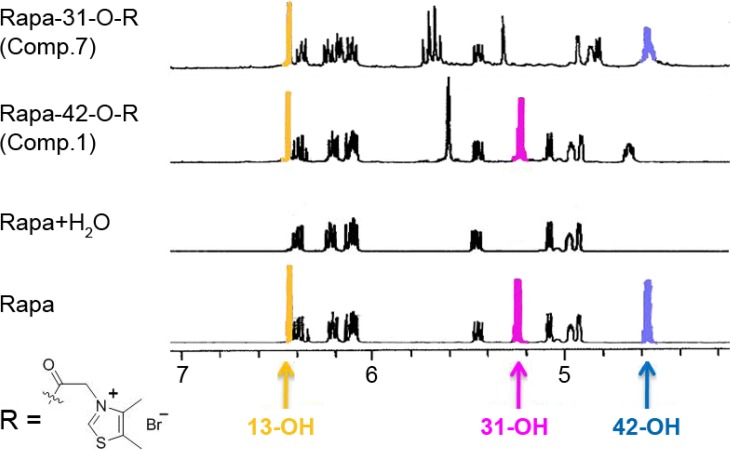
Expanded ^1^H-NMR spectra (600 MHz, DMSO-*d*_6_) for compounds Rapa, **1** and **7**.

In order to verify the structures of these 11 derivatives, 2D NMR data including HSQC, HMBC, COSY, TOCSY and ROESY were utilized to assign ^1^H and ^13^C-NMR signals (Supplementary Material). For some particular groups, such as the -OH, the combination of hydrogen-deuterium exchange and HMBC technique were used to assign their chemical shifts. Specifically, the 13-OH, 31-OH and 42-OH signals were first located by the hydrogen-deuterium exchange method, and then accurately assigned at 6.44, 5.25 and 4.57 ppm by relevant HMBC correlations, respectively (Table 2). As shown in Figure 5, the three characteristic peaks could serve as markers to locate the position where the substituent group was linked to Rapa.

**Table 1:** Structures and solubility for compounds **1**–**11**.

Comp.	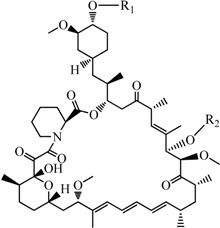		Aqueous Solubility (mg/mL)	Times Increased of Aqueous Solubility (Comp. *vs*. Rapa)
	R_1_	R_2_		
**Rapa**	H	H	0.0026	1
**1**	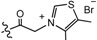	H	4.2	1615
**2**	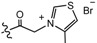	H	0.028	11
**3**	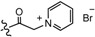	H	0.025	10
**4**	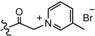	H	6.7	2577
**5**	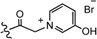	H	0.01	4
**6**	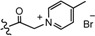	H	6	2308
**7**	H	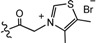	23.3	8962
**8**	H	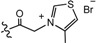	10	3846
**9**	H	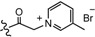	26.7	10269
**10**	H	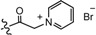	52.6	20231
**11**	H	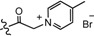	62.5	24038

**Table 2:** ^1^H (600 MHz) and ^13^C (150 MHz) NMR chemical shifts (δ, ppm) of Rapa, **1** and **7** in DMSO-*d*_6_.

C	Rapa		Comp. 1		Comp. 7	
	δC	δH	δC	δH	δC	δH
1	139.26	5.46	139.28	5.46	138.73	5.46
2	130.38	6.15	130.37	6.15	130.64	6.12
3	132.28	6.22	132.33	6.22	132.23	6.25
4	126.99	6.37	126.94	6.37	127.18	6.37
5	126.94	6.11	126.87	6.11	126.94	6.20
6	137.81	—	137.83	—	138.03	—
7	82.22	3.61	82.19	3.61	81.91	3.63
8	39.91	1.24,1.83	39.92	1.21,1.83	40.03	1.08,1.84
9	66.18	4.01	66.17	4.06	66.00	4.03
10	29.60	1.80,1.16	29.65	1.80,1.17	29.54	1.16,1.88
11	31.08	0.83,1.51	30.27	0.97,1.61	31.02	0.82,1.49
12	34.76	2.01	34.77	2.01	34.57	1.99
13	98.97	—	98.97	—	98.91	—
13-OH	—	6.44	—	6.49	—	6.45
15	198.84	—	198.76	—	198.21	—
16	166.95	—	166.97	—	167.00	—
18	43.45	3.41,3.15	43.46	3.42,3.15	43.67	2.99,3.43
19	24.42	1.55,1.26	24.33	1.28,1.56	24.41	1.28,1.57
20	20.32	1.65,1.38	20.31	1.65,1.38	20.40	1.43,1.69
21	26.39	2.08,1.56	26.40	1.59,2.08	26.27	1.60,2.20
22	50.72	4.93	50.81	4.93	51.25	4.93
23	169.15	—	169.24	—	169.46	—
25	73.55	4.98	73.57	4.97	73.56	4.87
26	39.50	2.72,2.37	39.50	2.79,2.38	40.03	2.34,2.66
27	207.48	—	207.47	—	207.03	—
28	45.16	3.25	45.18	3.24	45.07	3.20
29	124.93	5.08	124.73	5.08	124.30	4.81
30	137.09	—	137.06	—	138.03	—
31-OH	—	5.25	—	5.23	—	—
31	75.70	4.01	75.77	4.06	78.51	5.35
32	85.52	3.94	85.41	3.97	80.89	4.54
33	210.47	—	210.31	—	208.09	—
34	39.37	2.39	39.36	2.43	39.92	2.48
35	39.22	1.39,1.02	39.22	1.39,1.02	40.02	1.07,1.83
36	35.15	2.20	35.11	2.22	35.05	2.24
37	33.34	1.66	33.27	1.67	33.57	1.59
38	38.36	1.03,0.94	37.98	0.99,1.06	38.24	0.92,0.99
39	32.50	1.23	31.81	1.35	32.42	1.22
40	35.41	1.88,0.58	34.93	2.05,0.73	35.26	0.51,1.87
41	83.71	2.81	79.85	3.15	83.68	2.76
42	73.55	3.15	78.14	4.67	73.15	3.10
42-OH	—	4.57	—	—	—	4.56
43	32.86	1.73,1.15	35.11	2.01,2.21	32.75	1.16,1.74
44	31.08	1.52	26.40	1.55	31.02	1.56

**Figure 6:**
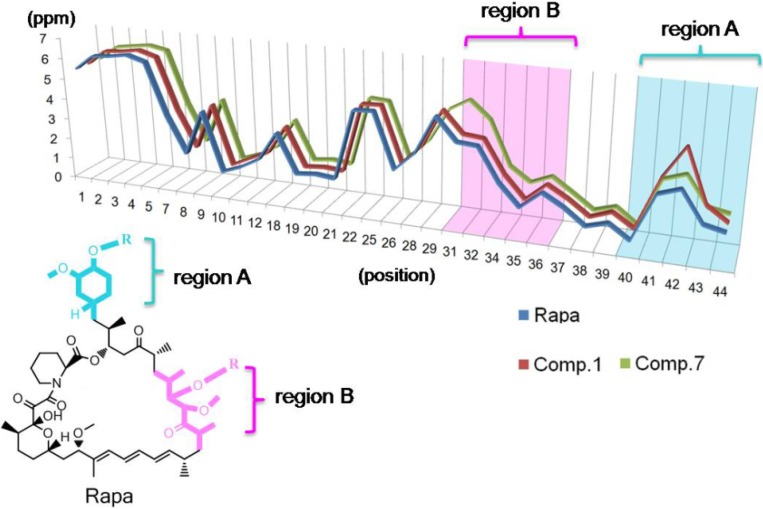
The curves (chemical shifts *vs*. positions) recording the chemical shifts (ppm) for different positions of protons of Rapa (blue curve), **1** (red curve) and **7** (green curve). Regions A and B were marked by blue and red, respectively.

An in-depth analysis of these NMR data revealed more information about the structures. Based on these NMR data (Table 2), a curve was drawn to record the chemical shifts (ppm) of different protons, and the profiles of Rapa, compounds **1** and **7** were put together for comparison (Figure 6). By this way, some structure information was deduced. The chemical shifts of most positions in compounds **1** and **7** are nearly identical, except for those of positions 39 to 44 (region A) and positions 29 to 36 (region B) in three cases (Figure 6). This phenomenon implied that the chemical environments of most of the protons were unchanged, furthermore, we inferred that most of the atoms in compounds **1** and **7** maintain a similar chemical environment as those in Rapa. Thus, the change of chemical shifts in region A and B could be used to locate the substituents directly.

Despite two years of efforts, we had never been able to obtain single crystals of these quaternary ammonium salt derivatives, so we tried to simulate the 3D structure of typical compounds **1** and **7**. Given the structure of rapamycin was extremely complicated, we attempted to construct this 3D structure based on the X-ray single crystal data of rapamycin reported by Swindel [16,17,18].

**Figure 7:**
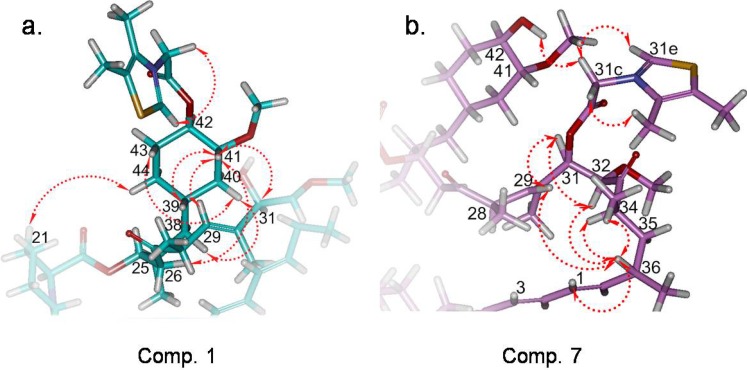
(**a**) Key partial simulated 3D structure of compound **1** (region A) and key ROESY correlations (red arrows); (**b**) Key partial simulated 3D structure of compound **7** (region B) and key ROESY correlations (red arrows).

By comparison of the NMR data, we decided to simulate the configuration of region A, region B and the side chain using the molecular modeling software SYBYL 6.5, and at the same time, maintain most of the 3D structure of rapamycin (Figure 7). The accuracy of this simulated 3D structure was confirmed by ROESY NMR experiments. In compound **1**, the existence of the side chain caused the cyclohexane ring to bend downward, leading to many distinct NOE effects of H41 to H21, H29, H31, H37, H38, H39, H40 and H42 (Figure 7a). In compound **7**, the molecule folds the side chain in region B placing it very closd to the cyclohexane ring in region A, and this situation could be proved easily by observing the NOE effect between H31c and 42-OH. Moreover, the ROESY correlations between H36 and H29, H32, H34 were clearly presented because of the obvious fold in the C26 to C36 region (Figure 7b).

All the above mentioned results not only proved that the reaction took place at the right positions, but also provided useful information regarding the three dimensional structures of these compounds, which might have a significant value in the field of compound design and structure simulation (Figure 7).

### 2.4. Aqueous Solubility

The aqueous solubility for all 11 compounds was improved remarkably (Table 1), and some (**10** and **11**) of them were even up to 25,000 times more soluble than Rapa. After summing up the effects of different substituents, a rule about the effect of substitution on aqueous solubility emerged. Generally speaking, the aqueous solubility of 31-Rapa derivatives is obviously superior to that of 42-Rapa derivatives.

## 3. Experimental

### 3.1. Physical Measurements

Melting points were determined using an RY-1 apparatus. MS spectra were obtained using an Applied Biosystems API-150EX LC/MS mass spectrograph. NMR data was recorded on a Varian Inova 600 MHz spectrometer operating at a proton frequency of 599.6 MHz and a carbon frequency of 150.8 MHz. All NMR spectra were acquired at 27 °C on a sample of 20 mg of rapamycin derivatives dissolved in 0.2 mL of DMSO-*d*_6_, with TMS as an internal standard. 3D-FTIR spectra were obtained from a ReactIR™ iC10 instrument equipped with a MCT Detector and a DiComp (Diamond) probe.

### 3.2. Synthesis

*Preparation of Rapa-42-ester of 2-(4,5-dimethylthiazolium-3-yl)acetic Acid* (**1**)

*Step 1*: Rapa (5 g, 5.5 mmol) and imidazole (3.74 g, 55 mmol) were dissolved in ethyl acetate (50 mL) and the solution was stirred at 0 °C, then a solution of trimethylchlorosilane (2.98 g, 27.5 mmol) in ethyl acetate (5 mL) was added into the reaction solution within 30 min. After the Rapa was transformed into Rapa-31,42-bis-OTMS, dilute sulfuric acid solution (0.5 mol/L, 10 mL) was slowly added dropwise to the reaction mixture within 3 h. The organic layer was washed three times with water. The organic layer was concentrated and dried over MgSO_4_. The residue was purified by chromatography on silica gel (EA/PE v/v = 1:5) to obtain Rapa-31-OTMS (4.7 g, 4.8 mmol, yield 88%).

*Step 2*: Rapa-31-OTMS (4.7 g, 4.8 mmol) and pyridine (3.7 g, 48 mmol) were added to dichloromethane at −10 °C, then, a solution of bromoacetyl bromide (4.8 g, 24 mmol) in dichloromethane (40 mL) was added into the reaction solution within 30 min. The solution was further stirred for 10 min, and warmed to 0–5 °C in 10 min. Then, dilute sulfuric acid (1 mol/L, 8 mL) was added to the reaction mixture over 1 h. This mixture was stirred for 30 min. The organic layer was washed three times with water. The combined organic layers were concentrated and dried over MgSO_4_. The residue was purified by chromatography on silica gel (EA/PE v/v = 1:8) to obtain Rapa-42-ester of bromoacetic acid (intermediate **I**) (3.82 g, 3.7 mmol, yield 77%).

*Step 3*: Intermediate **I** (3.82 g, 3.7 mmol) and 4,5-dimethylthiazole (5 equiv., 2.09 g, 18.5 mmol) were added to acetone (30 mL) at 20 °C, then, the reaction mixture was heated to 60 °C for 3 h. The solution was concentrated to dryness and the residue was purified by chromatography on silica gel (MeOH/DCM v/v = 1:5) to obtain compound 1 (3.61 g, yield 85%). m.p. 135–138 °C; FTIR (KBr): ν_max_/cm^−^^1^ = 3422 (OH) and 2931 (-CH_3_), 1723 (C=O), 1643 (C=O), 1451, 1091 (-C-O-C-); MS:1067.9 [M-Br]^+^; ^1^H-NMR (δ ppm): 10.03 (s, 1H, -S-CH=N-), 6.49 (s, 1H, 13-OH), 6.37 (m, 1H, 4-C), 6.21 (m, 1H, 3-C), 6.14 (m, 2H, 2-C), 5.62 (m, 2H, -CH_2_-), 5.46 (m, 1H, 1-C), 5.23 (s, 1H, 31-OH), 5.08 (s, 1H), 4.97 (s, 1H), 4.93 (s, 1H), 4.68 (s, 1H), 4.06 (s, 2H), 4.01 (s, 1H). ^13^C-NMR (δ ppm): 210.31, 207.47, 198.76, 169.24, 158.14, 141.91, 138.28, 130.37, 98.97. Anal. calcd for C_58_H_87_N_2_O_14_SBr: C 60.67, H 7.64, N 2.44, Br 6.96%. Found: C 60.49, H 7.66, N 2.45, Br 6.93%.

*Rapa-42-ester of 2-(4-methylthiazolium-3-yl)acetic Acid* (**2**). The procedure described above for the preparation of compound **1** was applied to intermediate **I** and 4-methylthiazole to afford the title compound **2** (yield 89%). m.p. 130–133 °C; FTIR (KBr): ν_max_/cm^−^^1^ = 3422 (OH) and 2933 (‑CH_3_), 1720 (C=O), 1643 (C=O), 1451, 1103 (-C-O-C-); MS:1053.8 [M-Br]^+^; ^1^H-NMR (δ ppm): 10.16 (d,1H, -S-CH=N-), 8.06 (s, 1H, -S-CH=), 6.45 (s,1H, 13-OH), 6.38 (m, 1H, 4-C), 6.22 (m, 1H, 3-C), 6.15 (m, 1H, 2-C), 5.62 (s, 2H, -CH_2_-), 5.46 (m, 1H, 1-C), 5.24 (s, 1H, 31-OH), 5.08 (d, 1H), 4.97 (s, 1H), 4.93 (s, 1H), 4.68 (m, 1H), 4.00 (s, 2H), 3.97 (s, 1H). ^13^C-NMR (δ ppm): 214.58, 178.64, 169.24, 164.76, 146.53, 120.99, 98.40, 67.14. Anal. calcd for C_57_H_85_N_2_O_14_SBr: C 60.36, H 7.55, N 2.47, Br 7.04%. Found: C 60.28, H 7.57, N 2.48, Br 7.00%. (89% yield).

*Rapa-42-ester of 2-(pyridinium-1-yl)acetic Acid* (**3**). The procedure described above for the preparation of compound **1** was applied to intermediate **I** and pyridine to afford the title compound **3** (yield 87%). m.p. 135–137 °C; FTIR (KBr): ν_max_/cm^−^^1^ = 3408 (OH) and 2934 (-CH_3_), 1738 (C=O), 1638 (C=O), 1452, 1104 (-C-O-C-); MS:1034.1 [M-Br]^+^; ^1^H-NMR (δ ppm): 9.07 (d, 1H, pyridine), 9.02 (m, 1H, pyridine), 8.71 (m, 1H, pyridine), 8.24 (m, 2H, pyridine), 6.45 (s, 1H, 13-OH), 6.41 (m, 1H, 4-C), 6.22 (m, 1H, 3-C), 6.14 (m, 1H, 2-C), 5.80 (m, 5H), 5.67 (m, 2H, -CH_2_-), 5.44 (m, 1H), 5.25 (s, 1H, 31-OH), 5.08 (m, 1H). ^13^C-NMR (δ ppm): 211.92, 208.23, 193.37, 169.24, 160.10, 145.63, 139.71, 130.17, 98.46. Anal. calcd for C_58_H_85_N_2_O_14_Br: C 62.52, H 7.69, N 2.51, Br 7.17%. Found: C 62.44, H 7.71, N 2.53, Br 7.13%.

*Rapa-42-ester of 2-(3-methylpyridinium-1-yl)acetic Acid* (**4**). The procedure described above for the preparation of compound **1** was applied to intermediate **I** and 3-methylpyridine to afford the title compound **4** (yield 82%). m.p. 138–140 °C; MS:1048 [M-Br]^+^; FTIR (KBr): ν_max_/cm^−^^1^ = 3422 (OH) and 2933 (-CH_3_), 1742 (C=O), 1641 (C=O), 1452, 1103 (-C-O-C-); ^1^H-NMR (δ ppm): 9.07 (d, 1H, pyridine), 9.02 (m, 1H, pyridine), 8.71 (m, 1H, pyridine), 8.14 (m, 2H, pyridine), 6.45 (s, 1H, 13-OH), 6.41 (m, 1H, 4-C), 6.22 (m, 1H, 3-C), 6.14 (m, 1H, 2-C), 5.80 (m, 5H), 5.67 (m, 2H), 5.44 (m, 1H), 5.25 (s, 1H, 31-OH), 5.08 (m, 1H). ^13^C-NMR (δ ppm) 214.60, 208.32, 169.25, 166.67, 165.55, 139.01, 98.41, 80.42. Anal. calcd for C_59_H_87_N_2_O_14_Br: C 62.81, H 7.77, N 2.48, Br 7.08%. Found: C 62.80, H 7.79, N 2.49, Br 7.05%.

*Rapa-42-ester of 2-(3-hydroxypyridinium-1-yl)acetic Acid* (**5**). The procedure described above for the preparation of compound **1** was applied intermediate **I** and 3-hydroxypyridine to afford the title compound **5** (yield 80%). m.p. 146–148 °C; FTIR (KBr): ν_max_/cm^−^^1^ = 3426 (OH) and 2933 (-CH_3_), 1721 (C=O), 1641 (C=O), 1452, 1102 (-C-O-C-); MS:1050 [M-Br]^+^; ^1^H-NMR (δ ppm): 12.08 (s, 1H, pyridine-OH), 8.60 (m, 1H, pyridine), 8.53 (m, 1H, pyridine), 8.02 (m, 2H, pyridine), 6.45 (m, 2H, 13-OH), 6.38 (s, 1H, 4-C), 6.22 (m, 1H, 3-C), 6.12 (m, 2H, 2-C), 5.60 (m, 2H), 5.48 (m, 1H), 5.25 (s, 1H, 31-OH), 5.08 (m, 1H). ^13^C-NMR (δ ppm): 214.41, 208.35, 169.29, 166.66, 164.97, 157.62, 139.53, 133.25, 98.43. Anal. calcd for C_58_H_85_N_2_O_14_Br: C 61.64, H 7.58, N 2.48, Br 7.07%. Found: C 61.56, H 7.60, N 2.49, Br 7.04%.

*Rapa-42-ester of 2-(4-methylpyridinium-1-yl)acetic Acid* (**6**). The procedure described above for the preparation of compound **1** was applied to intermediate **I** and 4-methylpyridine to afford the title compound **6** (yield 84%). m.p. 157–160 °C; FTIR (KBr): ν_max_/cm^−^^1^ = 3425 (OH) and 2933 (‑CH_3_), 1742 (C=O), 1643 (C=O), 1452, 1102 (-C-O-C-); MS:1048.1 [M-Br]^+^; ^1^H-NMR (δ ppm): 8.89 (d, 1H, pyridine), 8.05 (m, 2H, pyridine), 6.45 (s, 1H, 13-OH), 6.41 (m, 1H, 4-C), 6.22 (m, 1H, 3-C), 6.12 (m, 2H, 2-C), 5.61 (m, 3H), 5.46 (m, 1H), 5.25 (m, 1H, 31-OH), 5.08 (d, 1H), 4.93 (m, 2H). ^13^C-NMR (δ ppm): 214.65, 208.23, 192.96, 169.26, 160.09, 145.61, 139.70, 130.17, 98.46. Anal. calcd for C_59_H_87_N_2_O_14_Br: C 62.81, H 7.77, N 2.48, Br 7.08%. Found: C 62.73, H 7.80, N 2.49, Br 7.05%.

*Preparation of Rapa-31-ester of 2-(4,5-dimethylthiazolium-3-yl)acetic Acid* (**7**)

*Step 1*: Rapa (5 g, 5.5 mmol) and imidazole (3.74 g, 55 mmol) were dissolved in ethyl acetate (50 mL) and the solution was stirred at 0 °C, then a solution of trimethylchlorosilane (2.98 g, 27.5 mmol) in ethyl acetate (5 mL) was added into the reaction solution within 30 min. After the Rapa was transformed into Rapa-31,42-bis-OTMS, dilute sulfuric acid (0.5 mol/L, 10 mL) solution was slowly added dropwise into the reaction mixture within 3 h. The organic layer was washed three times with water. The organic layer was concentrated and dried over MgSO_4_. The residue was purified by chromatography on silica gel (EA/PE v/v = 1:5) to obtain Rapa-31-OTMS (4.6 g, 4.7 mmol, yield 88%).

*Step 2*: To a mixture of Rapa-31-OTMS (4.6 g, 4.7 mmol) and imidazole (3.3 g, 48 mmol) in ethyl acetate (40 mL) at 0 °C, a solution of *tert*-butyldimethylsilylchlide (TBDMSCl) (3.54 g, 24 mmol) in ethyl acetate (10 mL) was added within 30 min. After all the reagent was transformed into Rapa-31-OTMS-42-OTBDMS, dilute sulfuric acid (0.5 mol/L, 8 mL) was added into the solution within 1 h. The reaction solution was washed with water three times and the organic layer was concentrated and dried over MgSO_4_. The residue was purified by chromatography on silica gel (EA/PE v/v = 1:5) to obtain Rapa-42-OTBDMS (2.41 g, 2.4 mmol, yield 50%).

*Step 3*: Rapa-42-OTBDMS (2.41 g, 2.4 mmol) and pyridine (1.89 g, 24 mmol) were added into acetone (50 mL) at −10 °C. A solution of bromoacetyl bromide (5 equiv) in acetone (5 mL) was added into this mixture within 30 min and further stirred for 10 min. Then, the reaction was heated to 0–5 °C in 10 min, and dilute sulfuric acid (2 mol/L, 5 mL) was added into the solution in 30 min. The solution was concentrated to dryness and the residue was purified by chromatography on silica gel (EA/PE v/v = 1:8) to obtain Rapa-31-ester of bromoacetic acid (intermediate **II**) (0.94 g, 0.9 mmol, yield 38%).

*Step 4*: Intermediate **II** (0.94 g, 0.9 mmol) and 4,5-dimethylthiazole (5 equiv., 0.51 g, 4.5 mmol) were added to acetone (10 mL) at 20 °C, then, the reaction mixture was heated to 60 °C for 3 h. The solution was concentrated to dryness and the residue was purified by chromatography on silica gel (MeOH/DCM v/v = 1:5) to obtain compound 7 (0.72 g, 0.63 mmol, yield 70%). m.p. 125–127 °C; FTIR (KBr): ν_max_/cm^−1^ = 3421 (OH) and 2933 (-CH_3_), 1720 (C=O), 1643 (C=O), 1452, 1104 (-C-O-C-); MS:1067.7 [M-Br]^+^; ^1^H-NMR (δ ppm): 10.02 (d, 1H, -S-CH=N-), 6.45 (s,1H, 13-OH), 6.37 (m, 1H, 4-C), 6.25 (m, 1H, 3-C), 6.20 (m, 1H, 5-C), 6.15 (m, 1H, 2-C), 5.70 (m, 2H), 5.45 (m, 1H), 5.33 (s, 1H), 5.08 (s, 1H), 4.93 (s, 1H), 4.81 (m, 1H), 4.56 (m, 3H, 42-OH). ^13^C-NMR (δ ppm): 208.09, 207.03, 198.21, 169.46, 157.92, 142.01, 138.03, 130.64, 98.91. Anal. calcd for C_58_H_87_N_2_O_14_SBr: C 60.67, H 7.64, N 2.44, Br 6.96%. Found: C 60.53, H 7.65, N 2.46, Br 6.93%.

*Rapa-31-ester of 2-(4-methylthiazolium-3-yl)acetic Acid* (**8**). The procedure described above for the preparation of compound **7** was applied to intermediate **II** and 4-methylthiazole to afford the title compound **8** (yield 62%). m.p. 130–132 °C; FTIR (KBr): ν_max_/cm^−1^ = 3420 (OH) and 2932 (-CH_3_), 1746 (C=O), 1642 (C=O), 1452, 1106 (-C-O-C-); MS:1054 [M-Br]^+^; ^1^H-NMR (δ ppm): 10.16 (d,1H, -S-CH=N-), 8.05 (s, 1H, S-CH=C), 6.45 (s, 1H, 13-OH), 6.37 (m, 1H, 4-C), 6.25 (m, 1H, 3-C), 6.18 (m, 1H, 5-C), 5.70 (s, 2H), 5.46(m, 1H), 5.35 (s, 1H), 4.55 (d, 2H, 42-OH). ^13^C-NMR (δ ppm): 214.58, 178.64, 169.24, 164.76, 146.53, 120.99, 98.40, 67.14. Anal. calcd for C_57_H_85_N_2_O_14_SBr: C 60.36, H 7.55, N 2.47, Br 7.04%. Found: C 60.28, H 7.57, N 2.48, Br 7.00%.

*Rapa-31-ester of 2-(3-methylpyridinium-1-yl)acetic Acid* (**9**). The procedure described above for the preparation of compound **7** was applied to intermediate **II** and 3-methylpyridine to afford the title compound **9** (yield 82%). m.p. 138–140 °C; FTIR (KBr): ν_max_/cm^−1^ = 3424 (OH) and 2932 (-CH_3_), 1723 (C=O), 1641 (C=O), 1452, 1190 (-C-O-C-); MS:1048.1 [M-Br]^+^; ^1^H-NMR (δ ppm): 8.88 (d, 1H, pyridine), 8.80 (d, 1H, pyridine), 8.57 (m, 1H, pyridine), 8.12 (m, 1H, pyridine), 6.45 (m, 1H, 13-OH), 6.40 (m, 1H, 4-C), 6.23 (m, 1H, 3-C), 6.17 (m, 1H, 5-C), 5.81 (d, 1H), 5.74 (m, 2H), 5.47 (m, 1H), 5.28 (s, 1H), 4.58 (s, 1H, 42-OH). ^13^C-NMR (δ ppm): 212.91, 208.19, 169.30, 166.92, 164.60, 143.79, 139.41, 130.17, 98.29. Anal. calcd for C_59_H_87_N_2_O_14_Br: C 62.81, H 7.77, N 2.48, Br 7.08%. Found: C 62.73, H 7.79, N 2.50, Br 7.04%.

*Rapa-31-ester of 2-(pyridinium-1-yl)acetic Acid* (**10**). The procedure described above for the preparation of compound **7** was applied to intermediate **II** and pyridine to afford the title compound **10** (yield 59%). m.p. 140–142 °C; FTIR (KBr): ν_max_/cm^−1^ = 3425 (OH) and 2932 (-CH_3_), 1723 (C=O), 1640 (C=O), 1452, 1092 (-C-O-C-); MS: 1034 [M-Br]^+^; ^1^H-NMR (δ ppm): 8.96 (d, 2H, pyridine), 8.72 (m, 1H, pyridine), 8.22 (m, 2H, pyridine), 6.44 (s, 1H, 13-OH), 6.40 (m, 1H, 4-C), 6.24 (m, 1H, 3-C), 6.17 (m, 2H, 5-C), 5.87 (m, 1H), 5.79 (m, 1H), 5.48 (m, 1H), 5.29 (s, 1H), 4.95 (m, 2H), 4.81 (s, 1H), 4.52 (s, 1H, 42-OH). ^13^C-NMR (δ ppm): 208.14, 169.29, 166.94, 146.65, 140.02, 126.43, 98.29. Anal. calcd for C_58_H_85_N_2_O_14_Br: C 62.52, H 7.69, N 2.51, Br 7.17%. Found: C 62.44, H 7.71, N 2.52, Br 7.14%.

*Rapa-31-ester of 2-(4-methylpyridinium-1-yl)acetic Acid* (**11**). The procedure described above for the preparation of compound **7** was applied to intermediate **II** and 4-methylpyridine to afford the title compound **11** (yield 65%). m.p. 145–148 °C; FTIR (KBr): ν_max_/cm^−1^ = 3424 (OH) and 2932 (-CH_3_), 1723 (C=O), 1644 (C=O), 1452, 1092 (-C-O-C-); MS:1048.1 [M-Br]^+^; ^1^H-NMR (δ ppm): 8.77 (d, 2H, pyridine), 8.03 (d, 2H, pyridine), 6.44 (s, 1H, 13-OH), 6.40 (m, 1H, 4-C), 6.23 (m, 1H, 3-C), 6.16 (m, 2H, 5-C), 5.80 (d, 1H), 5.70 (d, 1H), 5.44 (m, 1H), 5.29 (m, 1H), 5.29 (s, 1H), 4.52 (s, 1H, 42-OH). ^13^C-NMR (δ ppm): 212.72, 169.35, 164.95, 160.41, 145.50, 139.79, 129.23, 98.17, 84.23. Anal. calcd for C_59_H_87_N_2_O_14_Br: C 62.81, H 7.77, N 2.48, Br 7.08%. Found: C 62.67, H 7.79, N 2.49, Br 7.05%.

## 4. Conclusions

The *in situ* IR technique was successfully applied in the selective synthesis of the key intermediate for preparing Rapa derivatives, which made the determination of the reaction endpoint easier so as to make the reaction high yield (88%) and controllable. In total 11 mono-substituted Rapa derivatives (six 42-Rapa derivatives and five 31-Rapa derivatives) were synthesized, and all of them had a quaternary ammonium salt structures. The aqueous solubility of all these compounds was obviously improved. Among them, the aqueous solubility of compounds **10** and **11** jumped to 62 mg/mL, nearly 25,000 times that of Rapa. The spatial configuration of these Rapa derivatives was simulated by the molecular modeling software SYBYL 6.5, which showed a good agreement with the 2D NMR data. This provides valuable information to guide further compound design and synthesis.

## Supplementary Materials

Supplementary materials can be accessed at: http://www.mdpi.com/1420-3049/19/6/7770/s1.
