# Supplementary material for: Selective Substitution of 31/42–OH in Rapamycin Guided by an in Situ IR Technique
Source: Molecules. 2014 Jun 10;19(6):7770–84. doi: 10.3390/molecules19067770 (PMC6271078; doi:10.3390/molecules19067770)

Supplementary File

Content

1. The selected COSY and HMBC correlations exhibited by compound **1**…………………………...S1
2. ^{1}H-NMR spectra of Rapa-31-OTMS and Rapa-31, 42-OTMS……………………………………..S2
3. 1D, 2D NMR, LCMS and IR spectra of compound **1**–**11**…………………………………….….S3

1. The Complete Simulate 3D Structure and Selected COSY and HMBC Correlations Exhibited by Compound 1

Figure S1. Selected COSY and HMBC correlations exhibited by compound **1**.

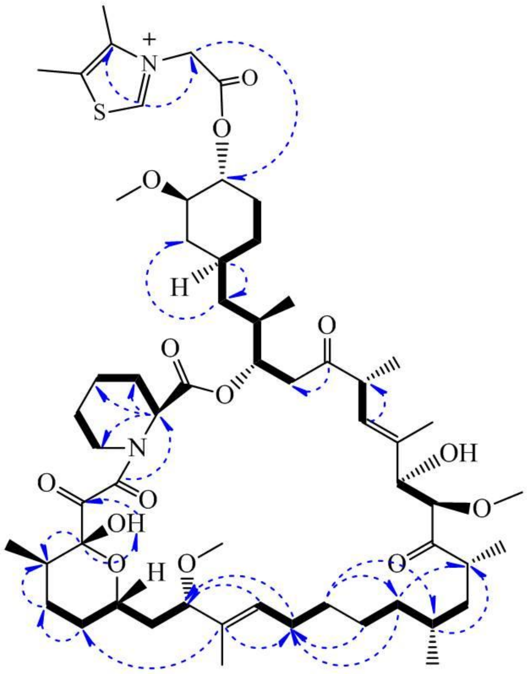

2. ^{1}H-NMR Spectra of Rapa-31-OTMS and Rapa-31, 42-OTMS

Figure S2. ^{1}H-NMR spectra of Rapa-31-OTMS.

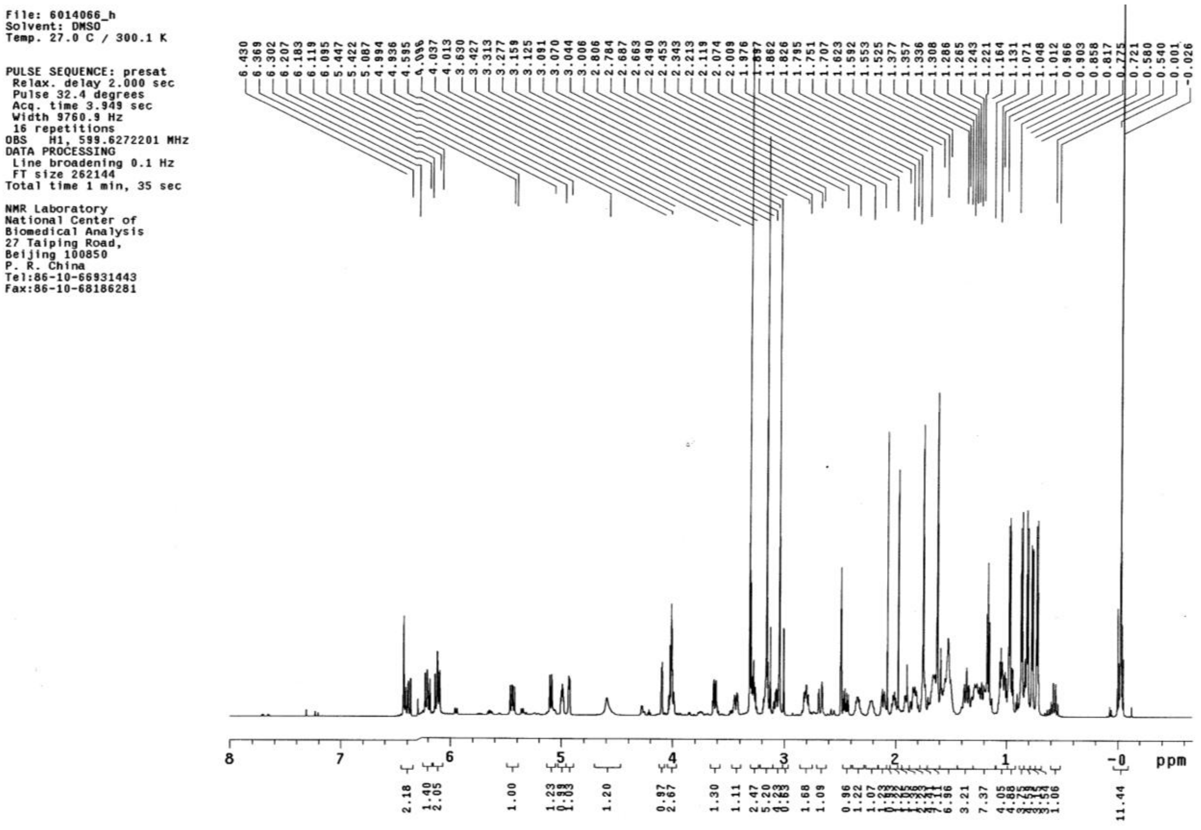

Figure S3. ^{1}H-NMR spectra of Rapa-31, 42-OTMS.

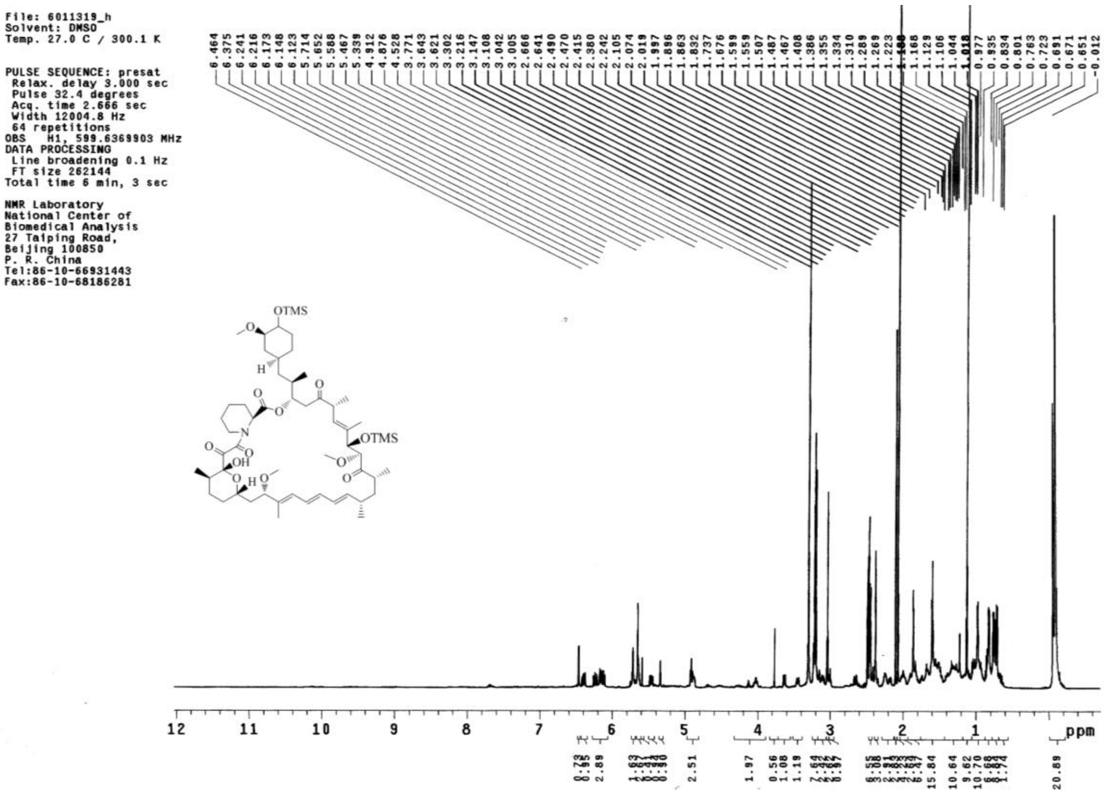

3. 1D, 2D NMR, LCMS and IR Spectra of Compound 1–11

Figure S4. ^{1}H-NMR of compound **1**.

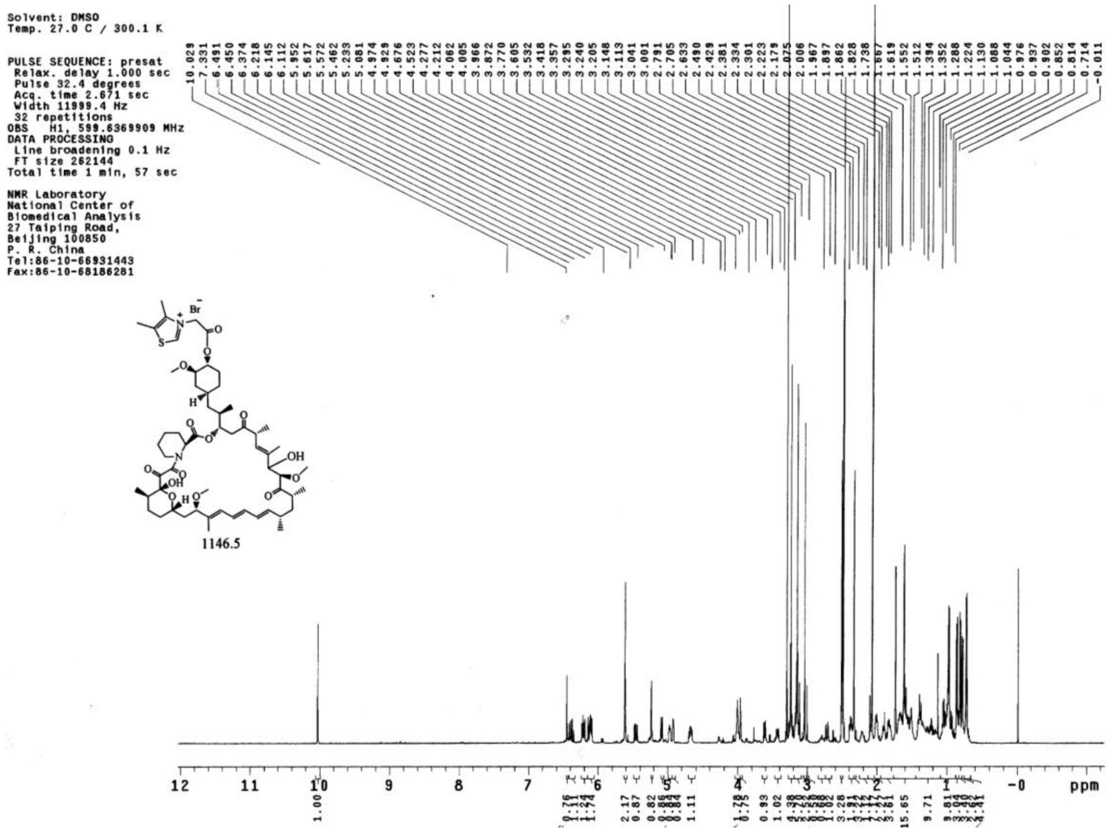

Figure S5. ^{13}C-NMR of compound **1**.

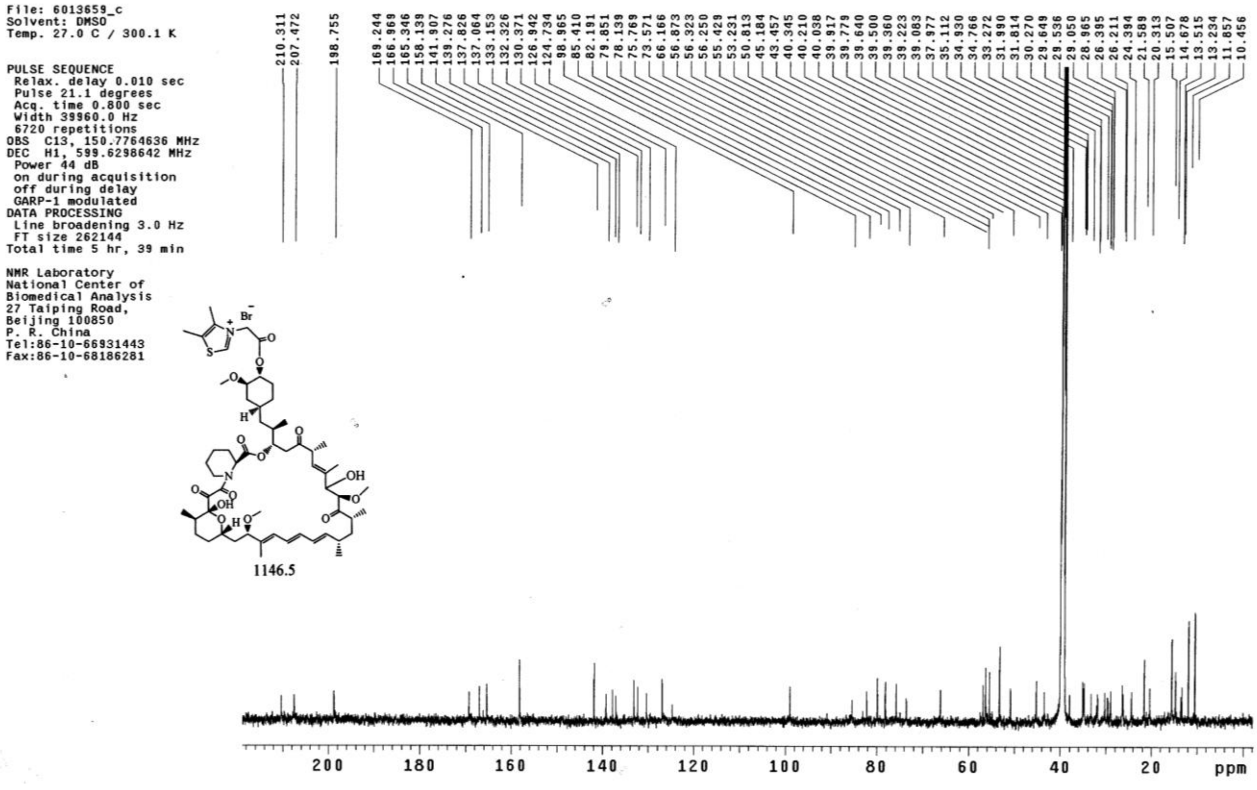

Figure S6. HSQC of compound **1**.

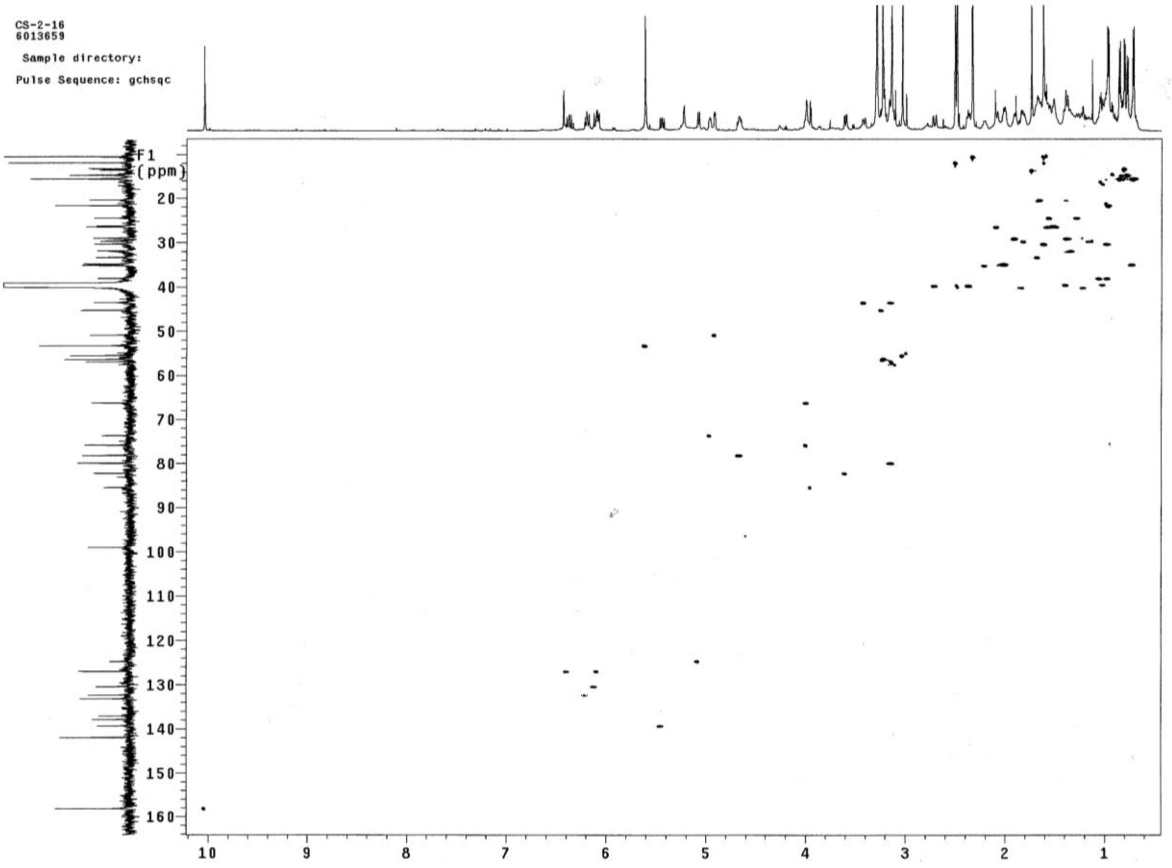

Figure S7. HMBC of compound **1**.

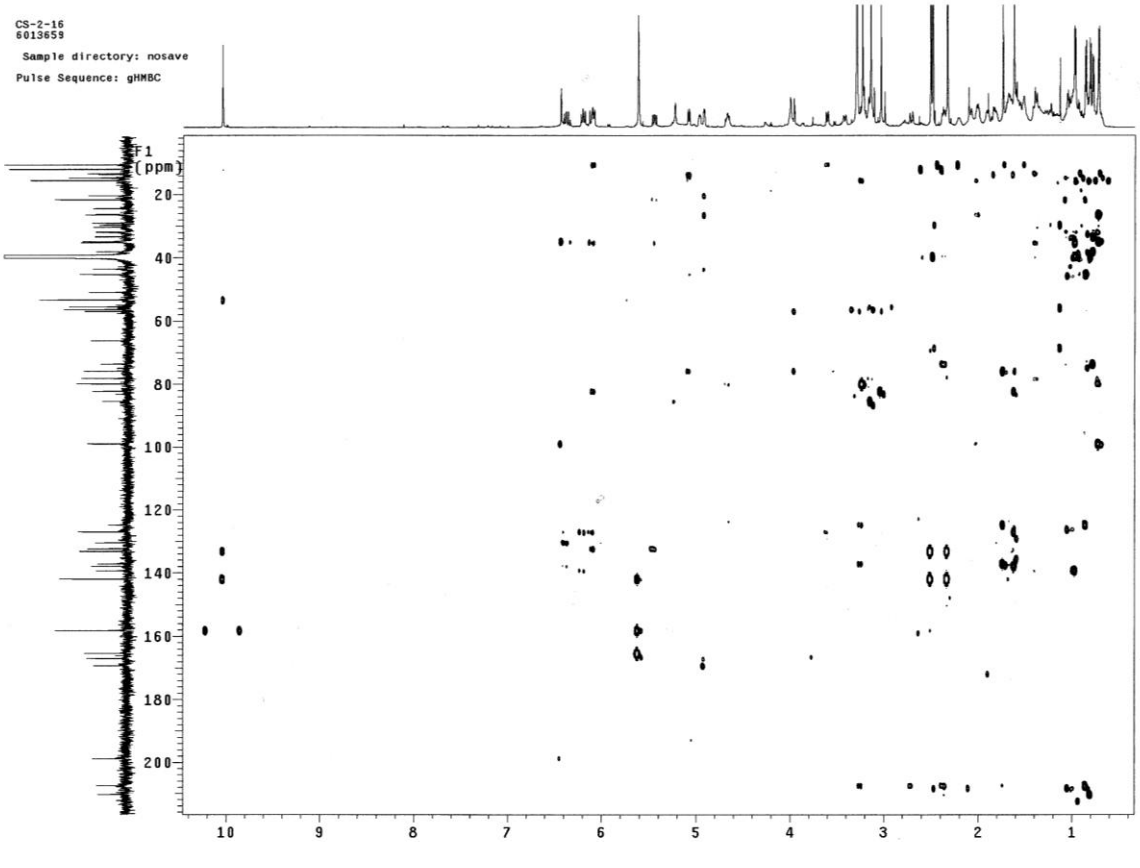

Figure S8. COSY of compound **1**.

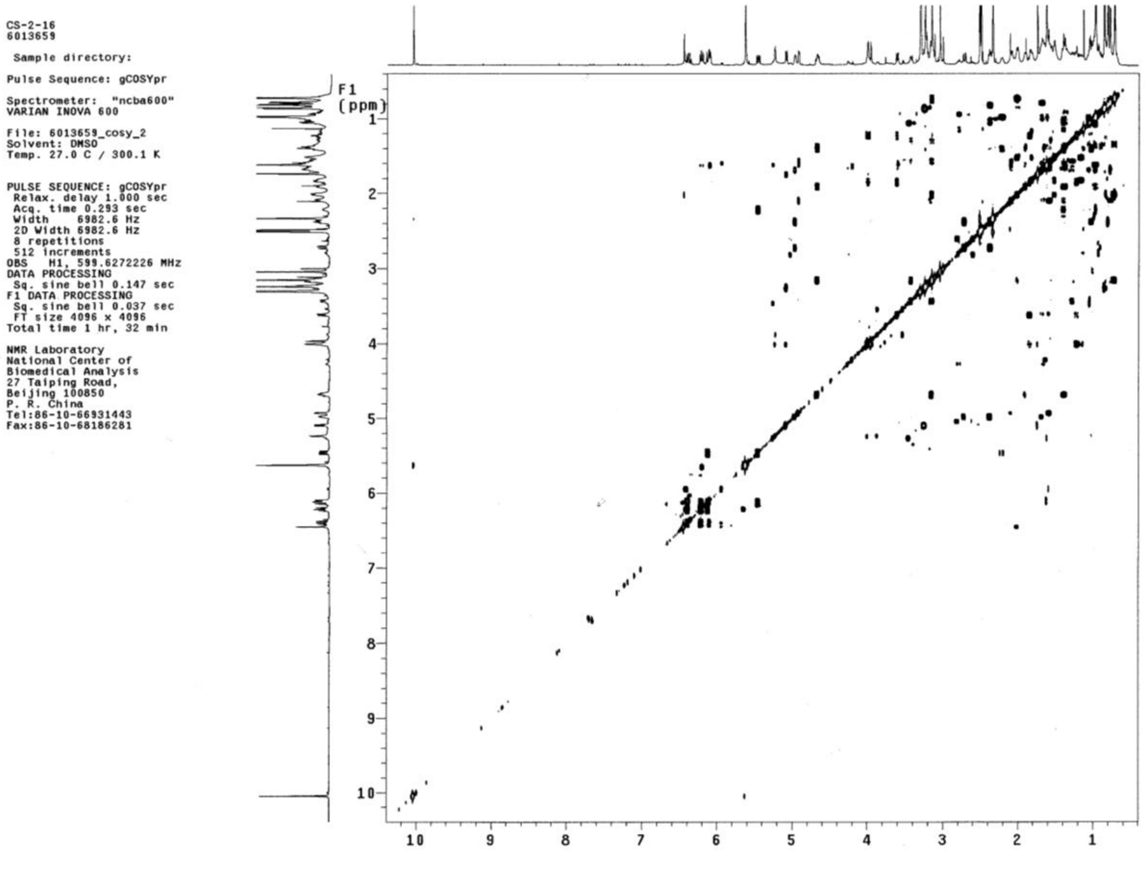

Figure S9. ROESY of compound **1**.

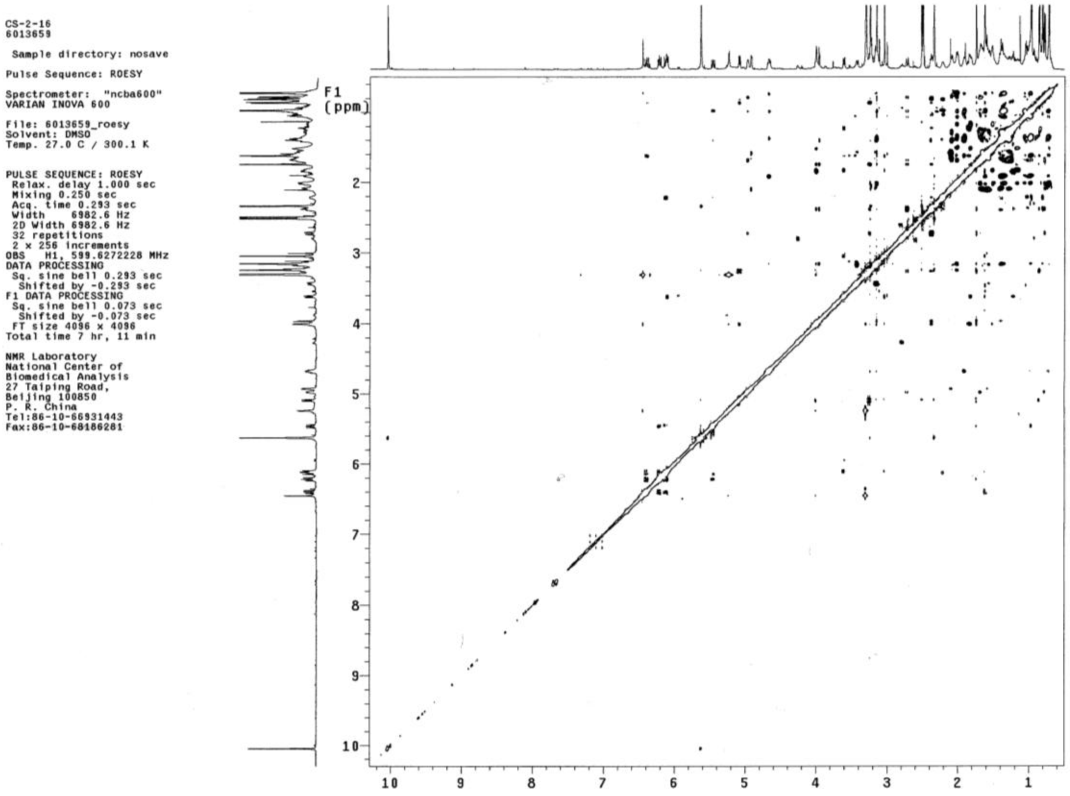

Figure S10. TOCSY of compound **1**.

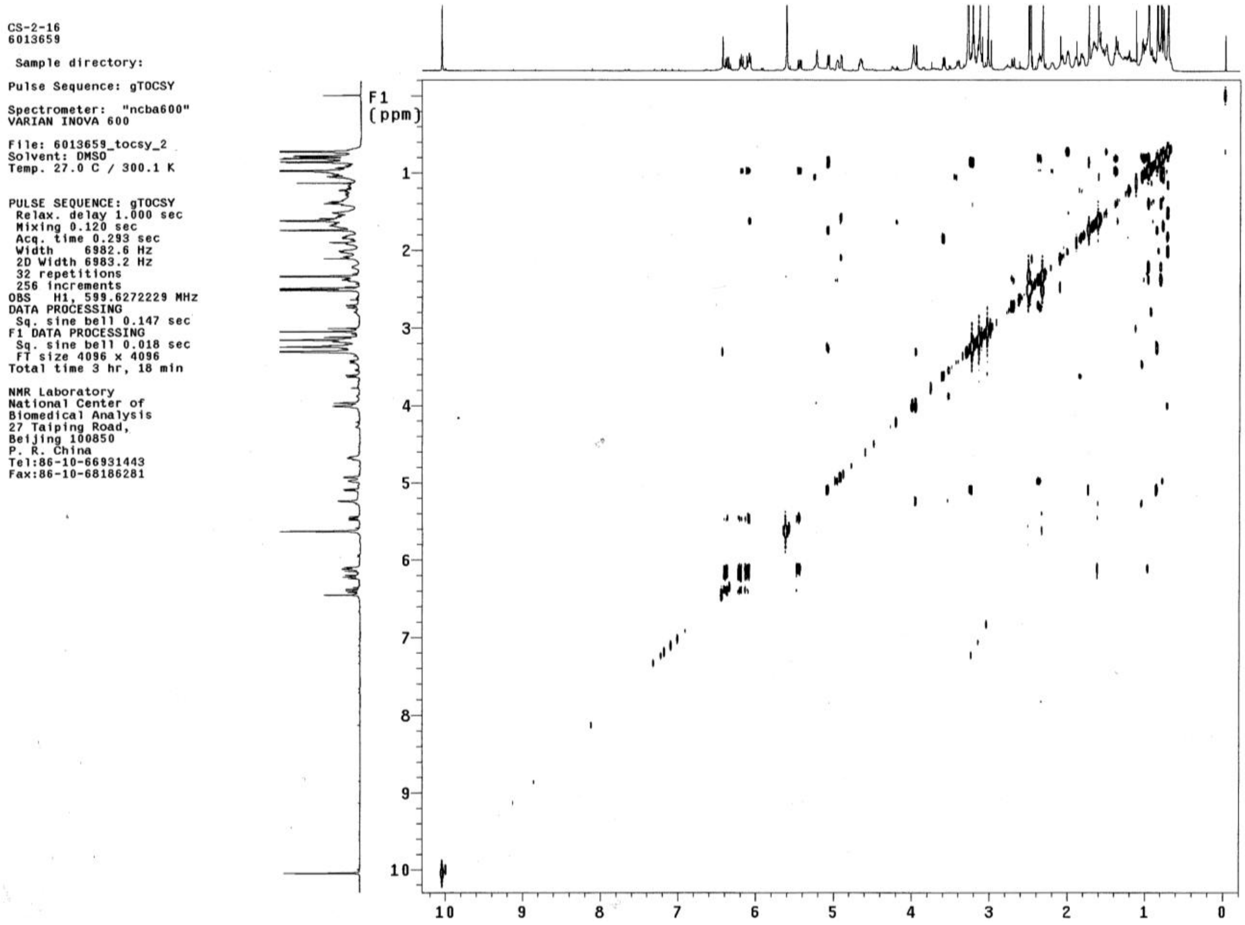

Figure S11. MS of compound **1**.

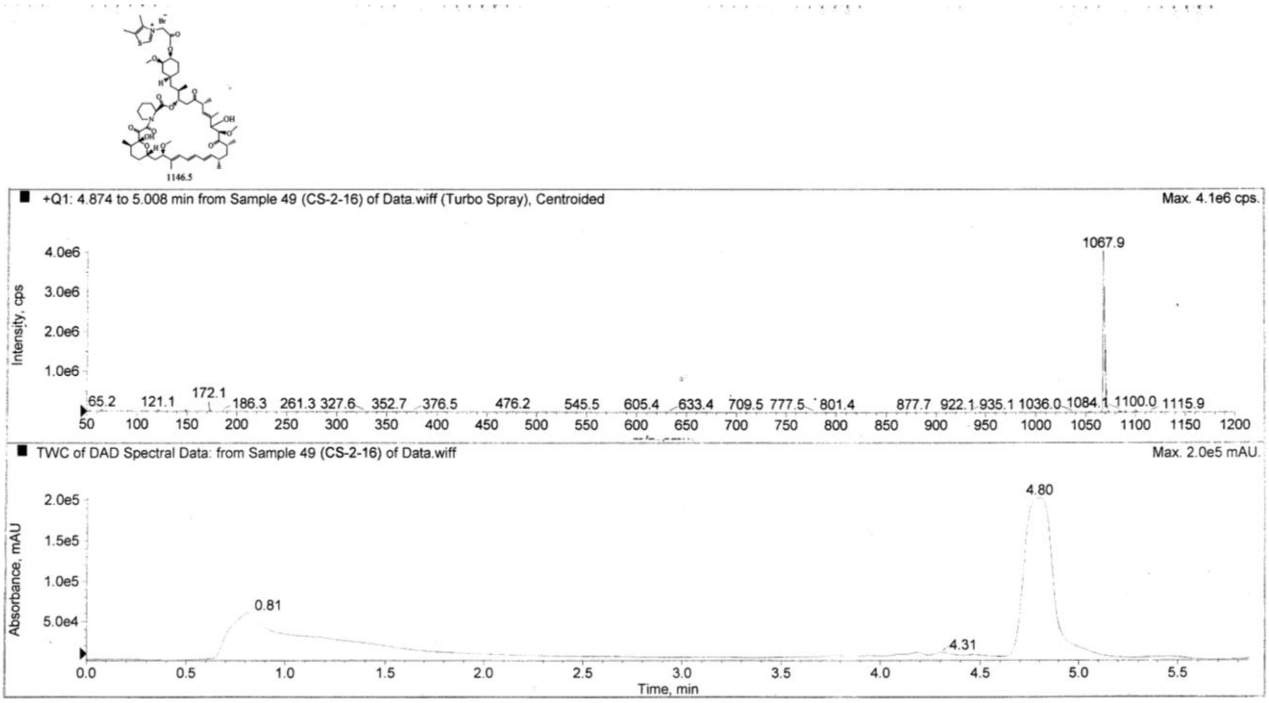

Figure S12. IR of compound **1**.

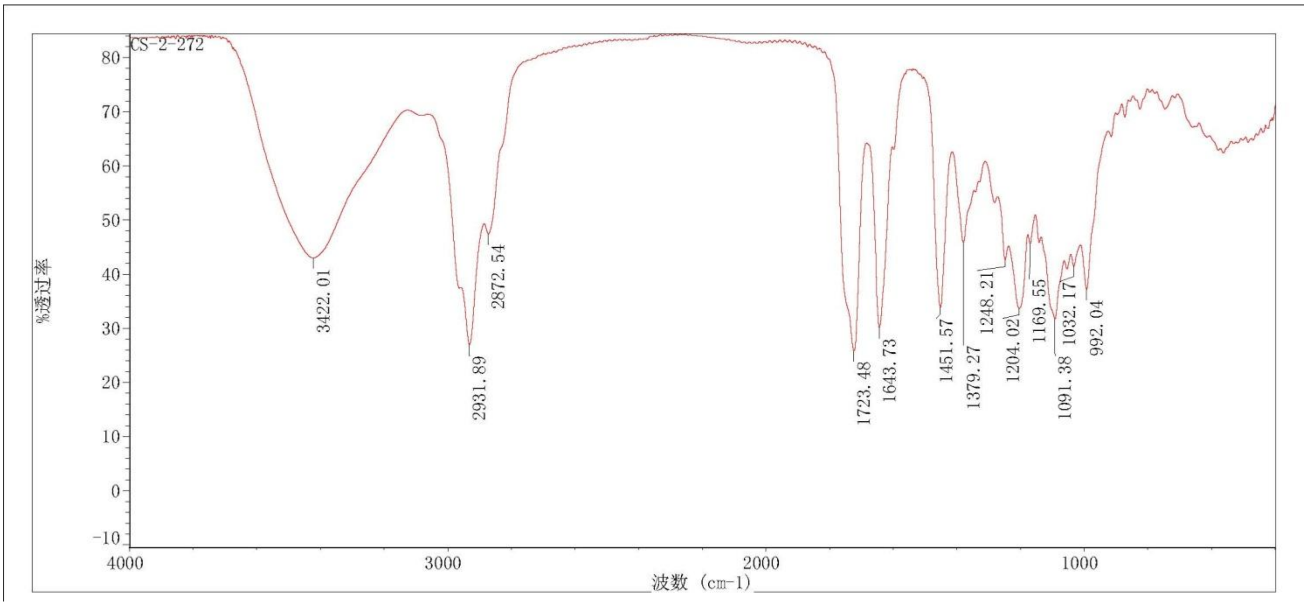

Figure S13. ^{1}H-NMR of compound **2**.

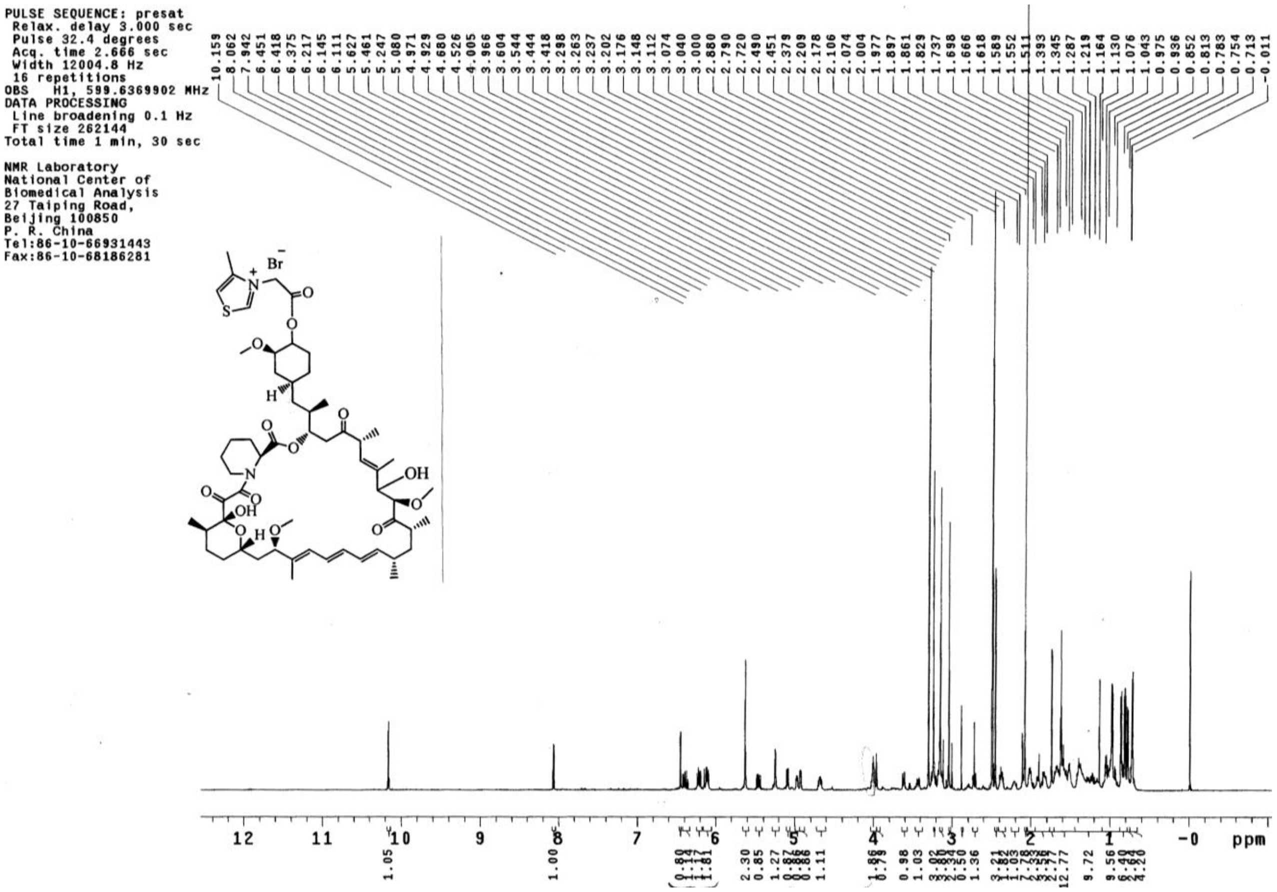

Figure S14. ^{13}C-NMR of compound **2**.

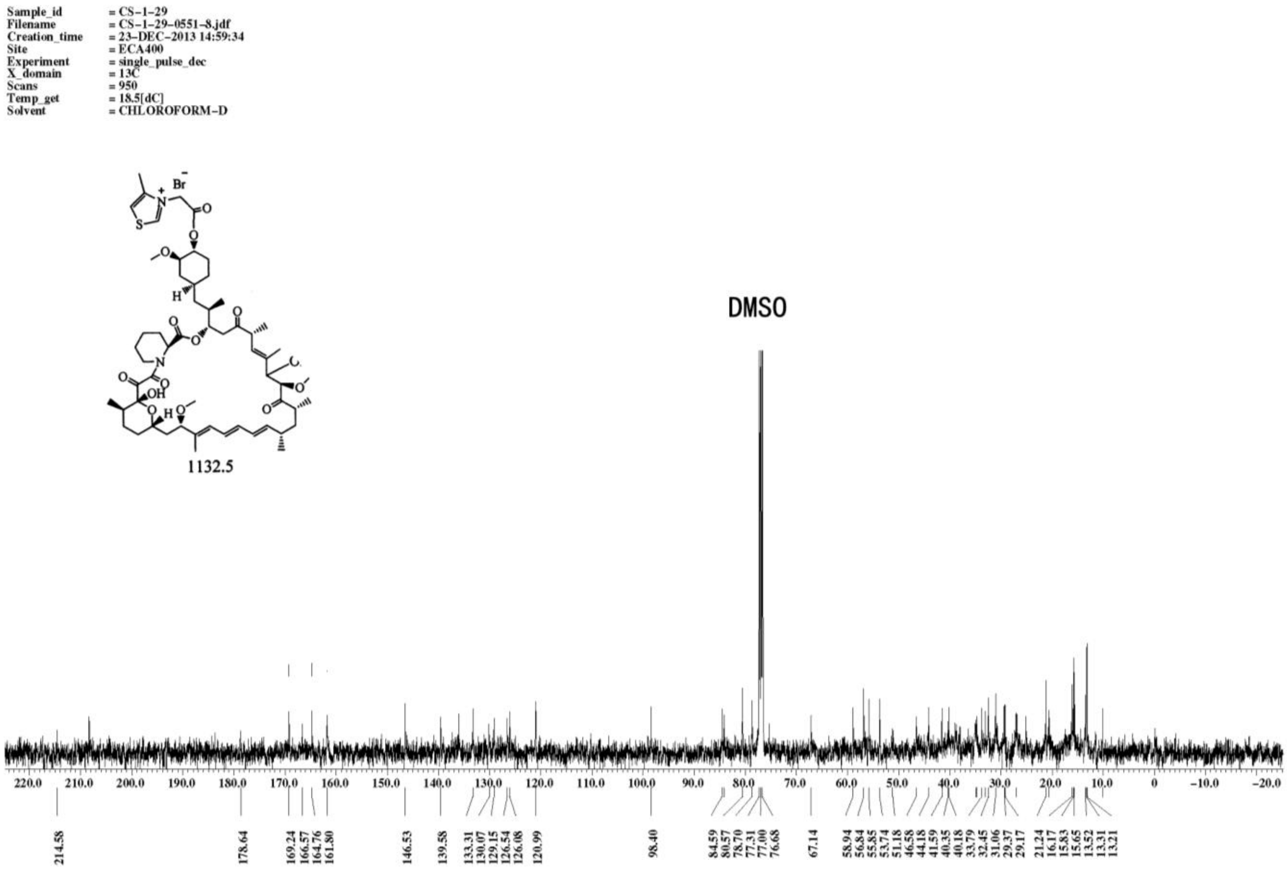

Figure S15. MS of compound **2**.

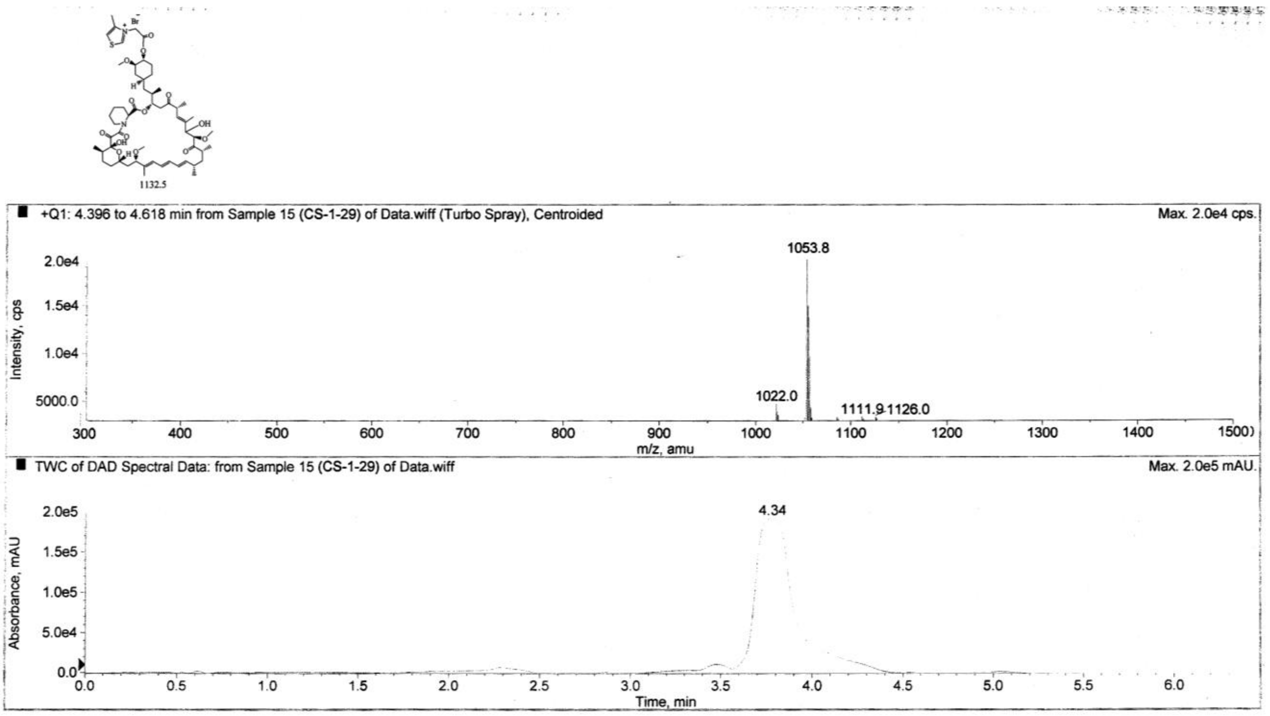

Figure S16. IR of compound **2**.

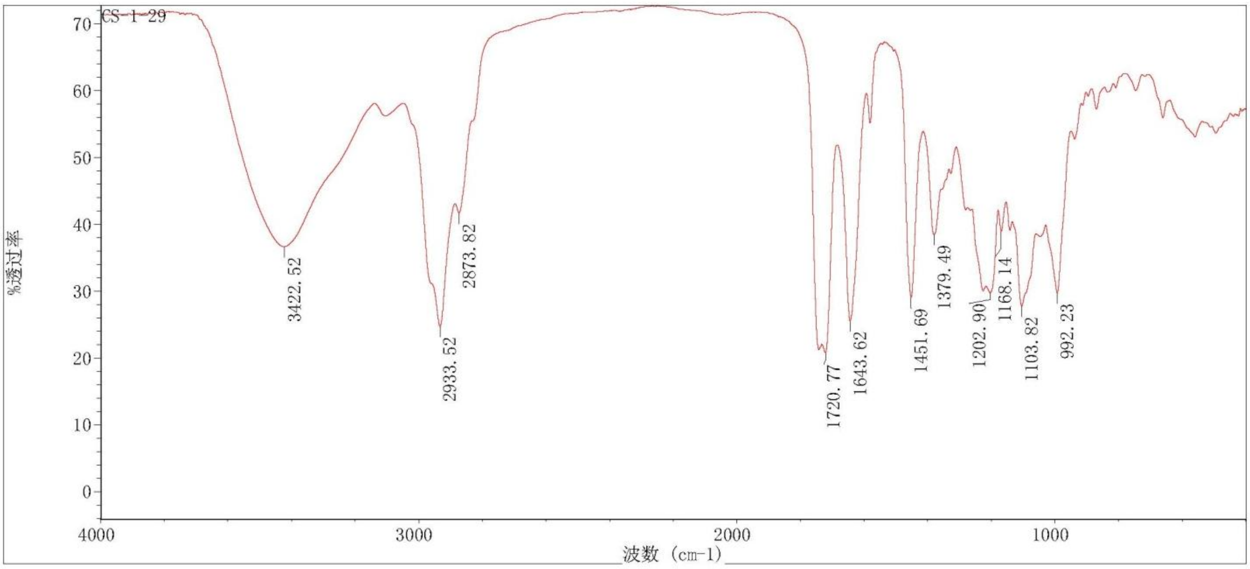

Figure S17. ^{1}H-NMR of compound **3**.

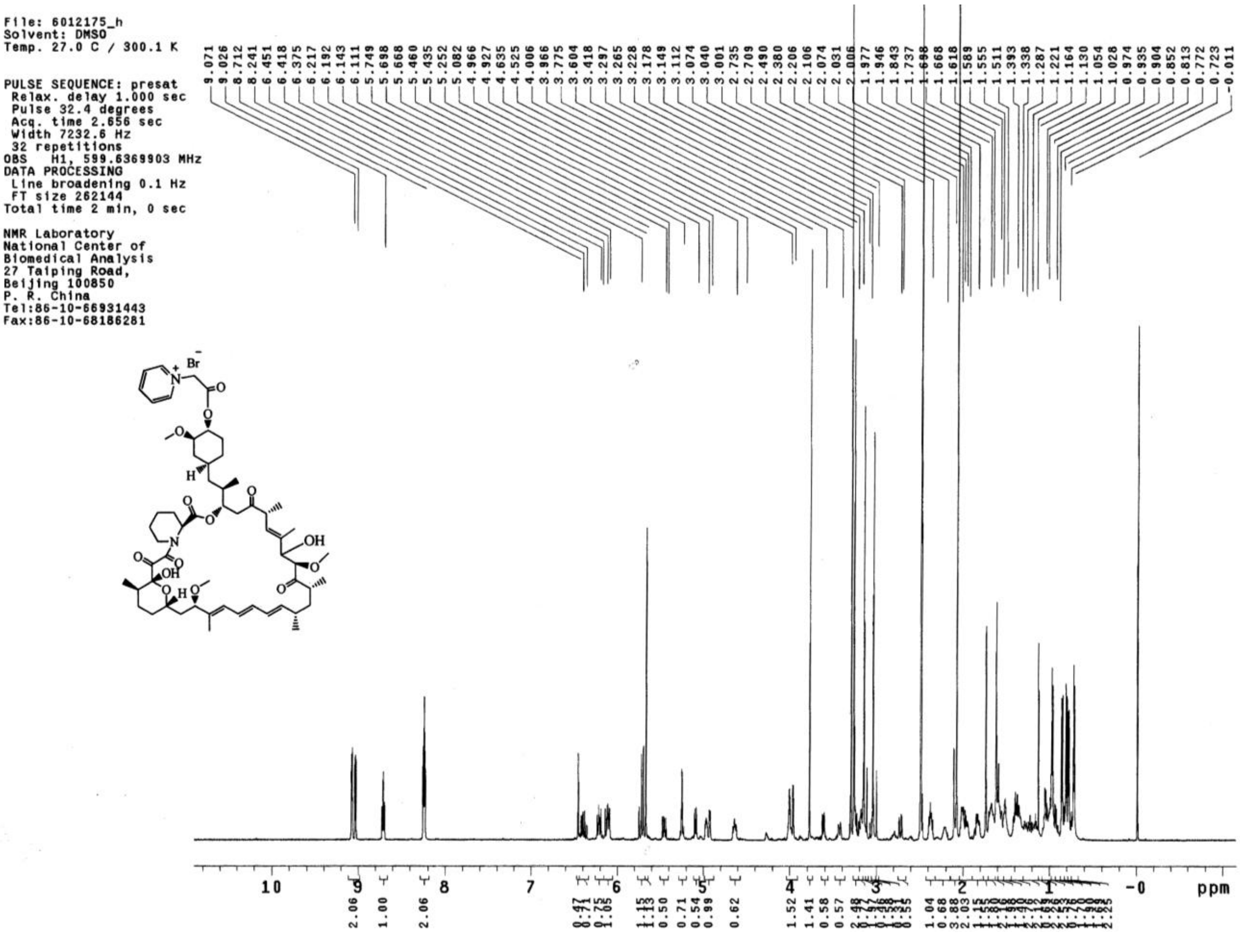

Figure S18. ^{13}C-NMR of compound **3**.

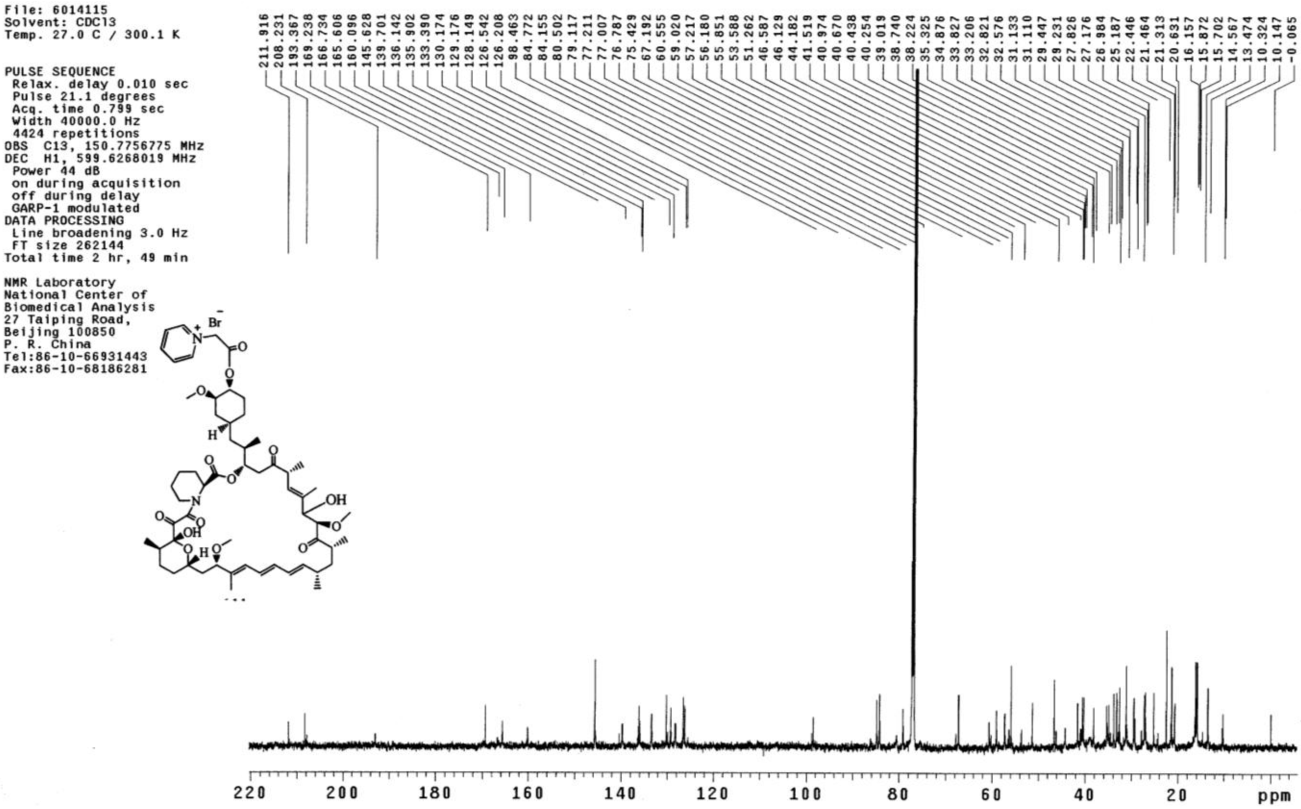

Figure S19. MS of compound **3**.

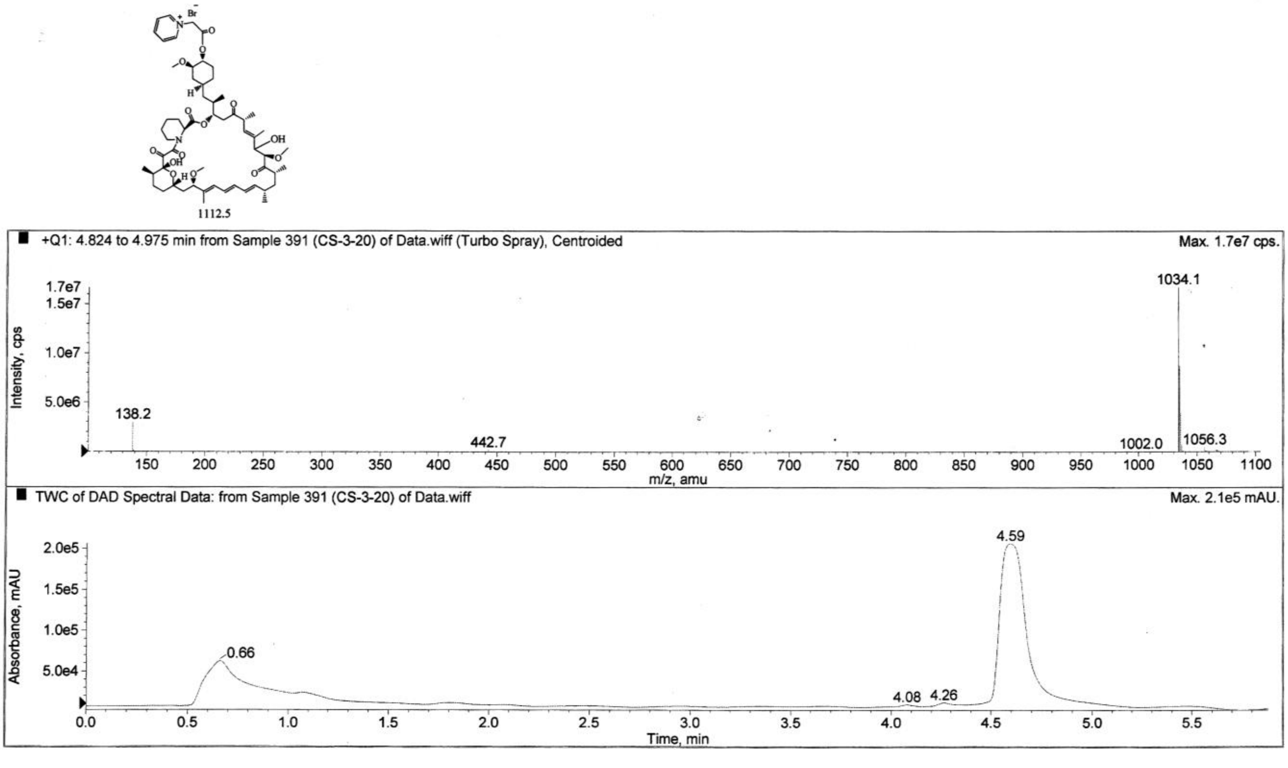

Figure S20. IR of compound **3**.

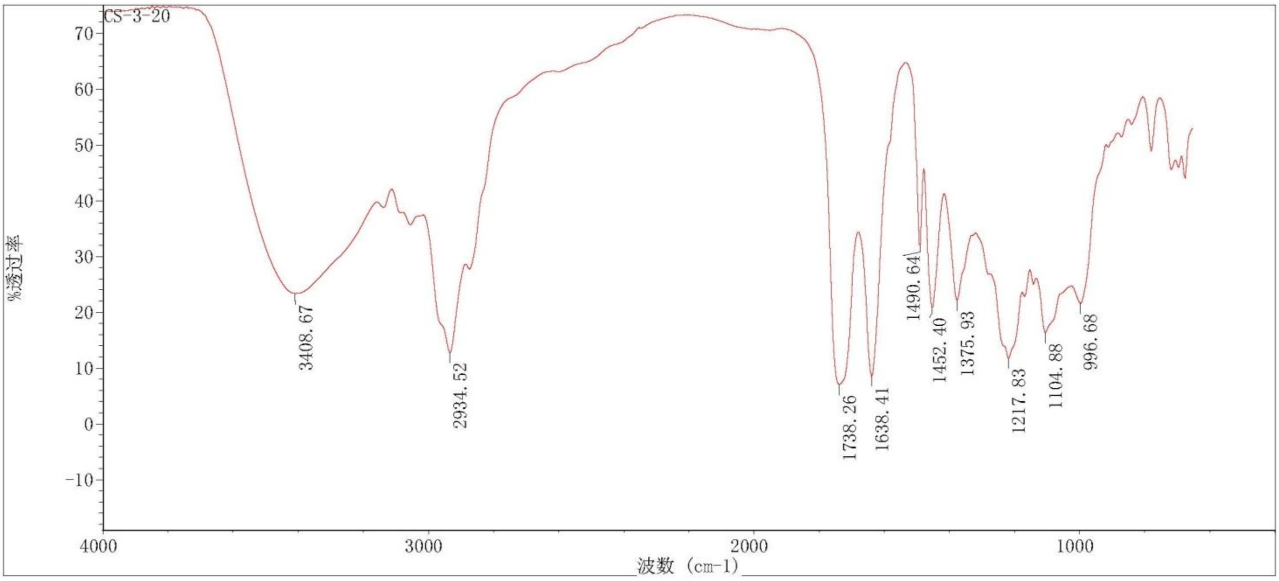

Figure S21. ^{1}H-NMR of compound **4**.

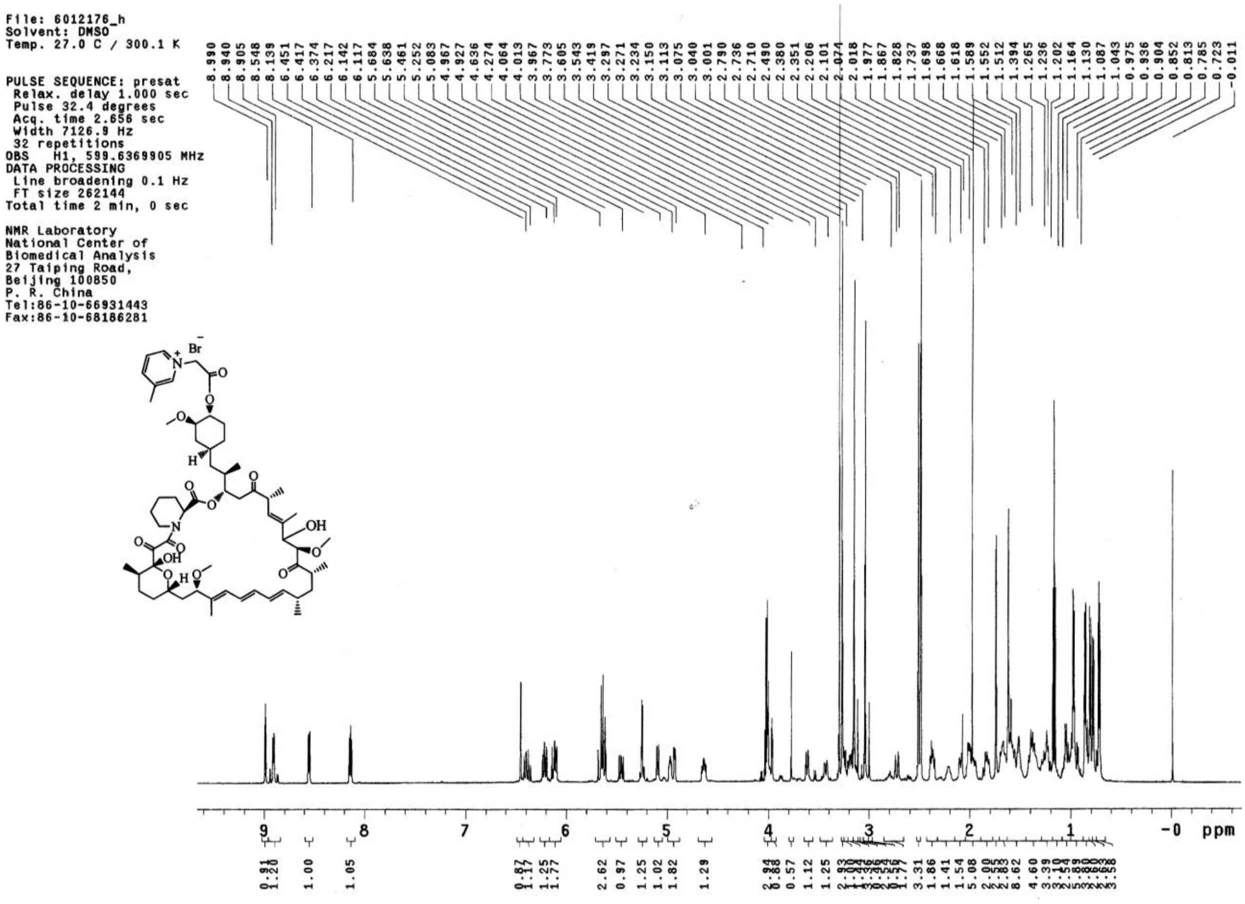

Figure S22. ^{13}C-NMR of compound **4**.

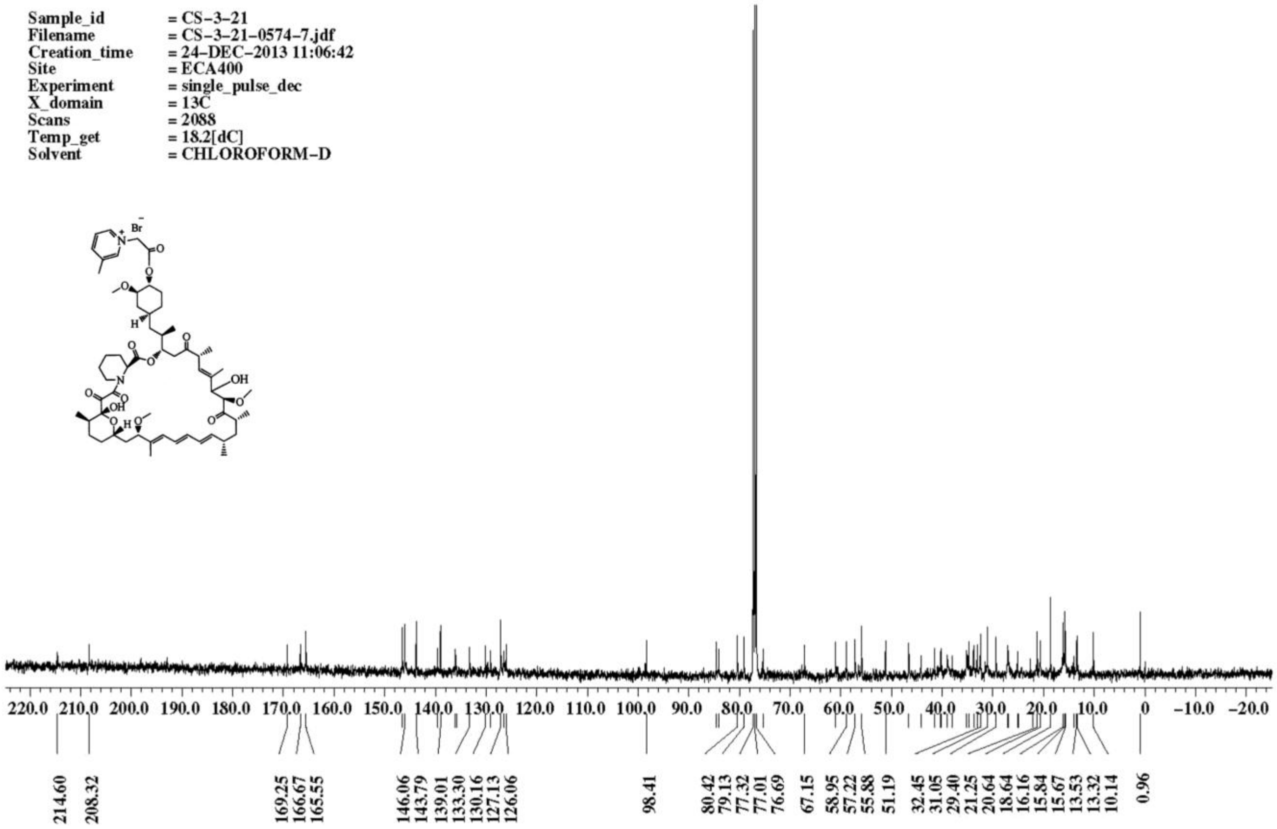

Figure S23. MS of compound **4**.

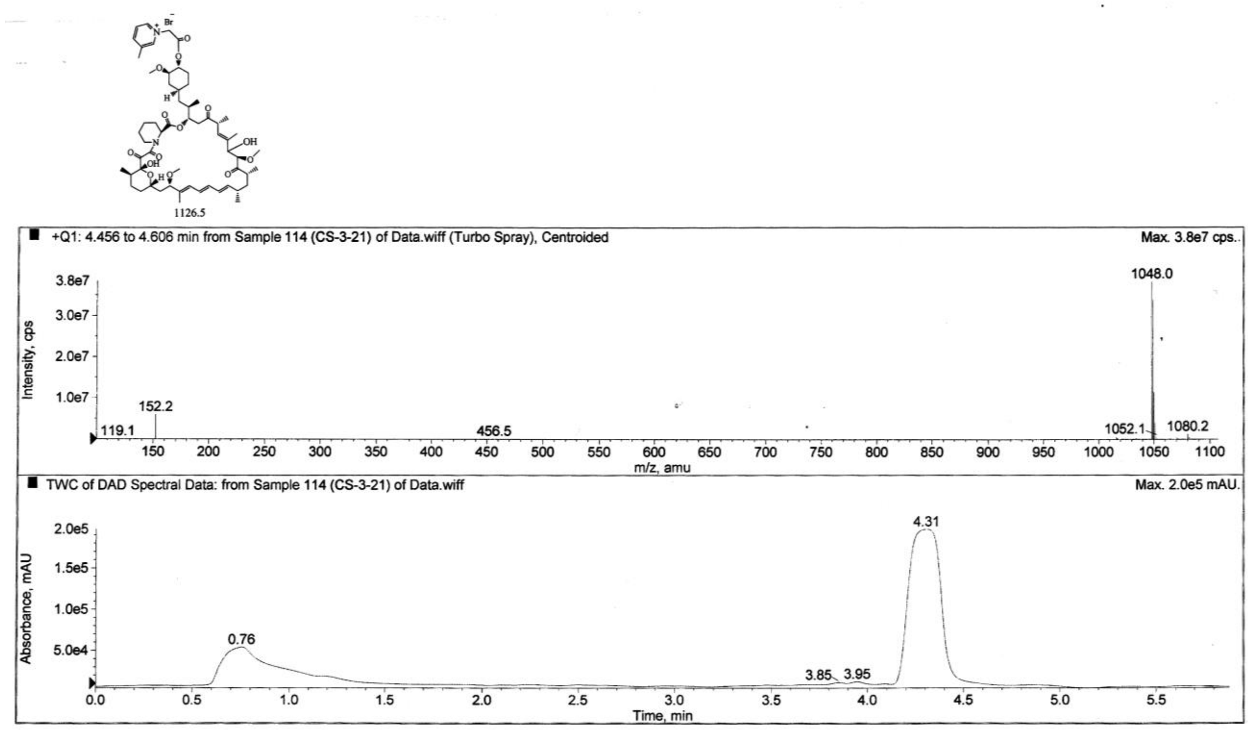

Figure S24. IR of compound **4**.

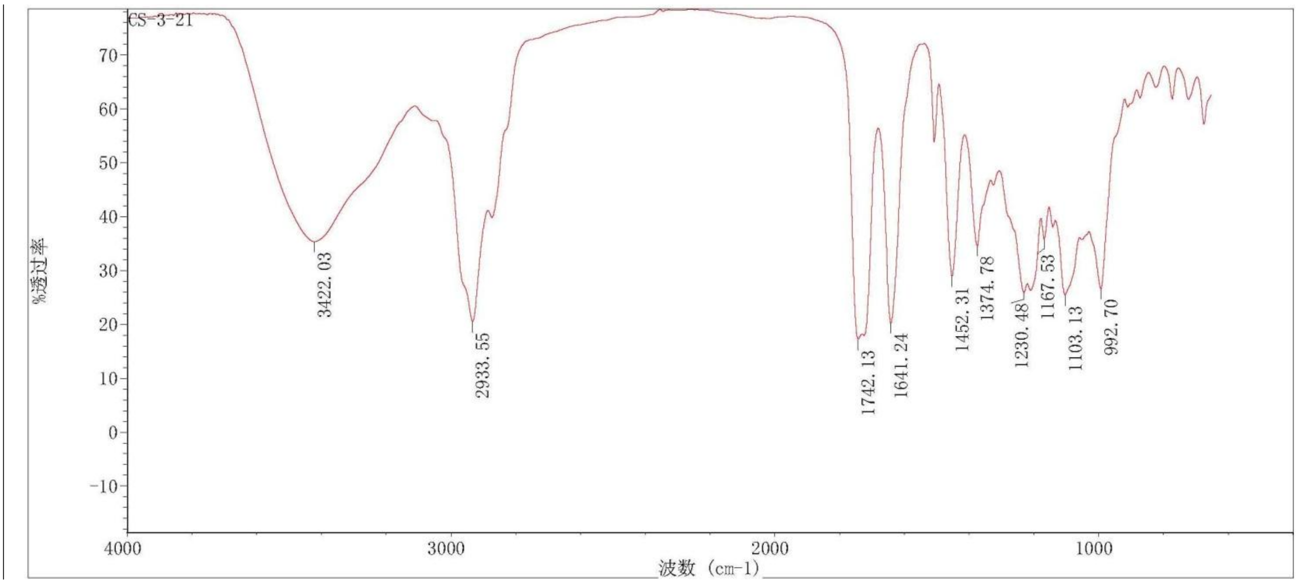

Figure S25. ^{1}H-NMR of compound **5**.

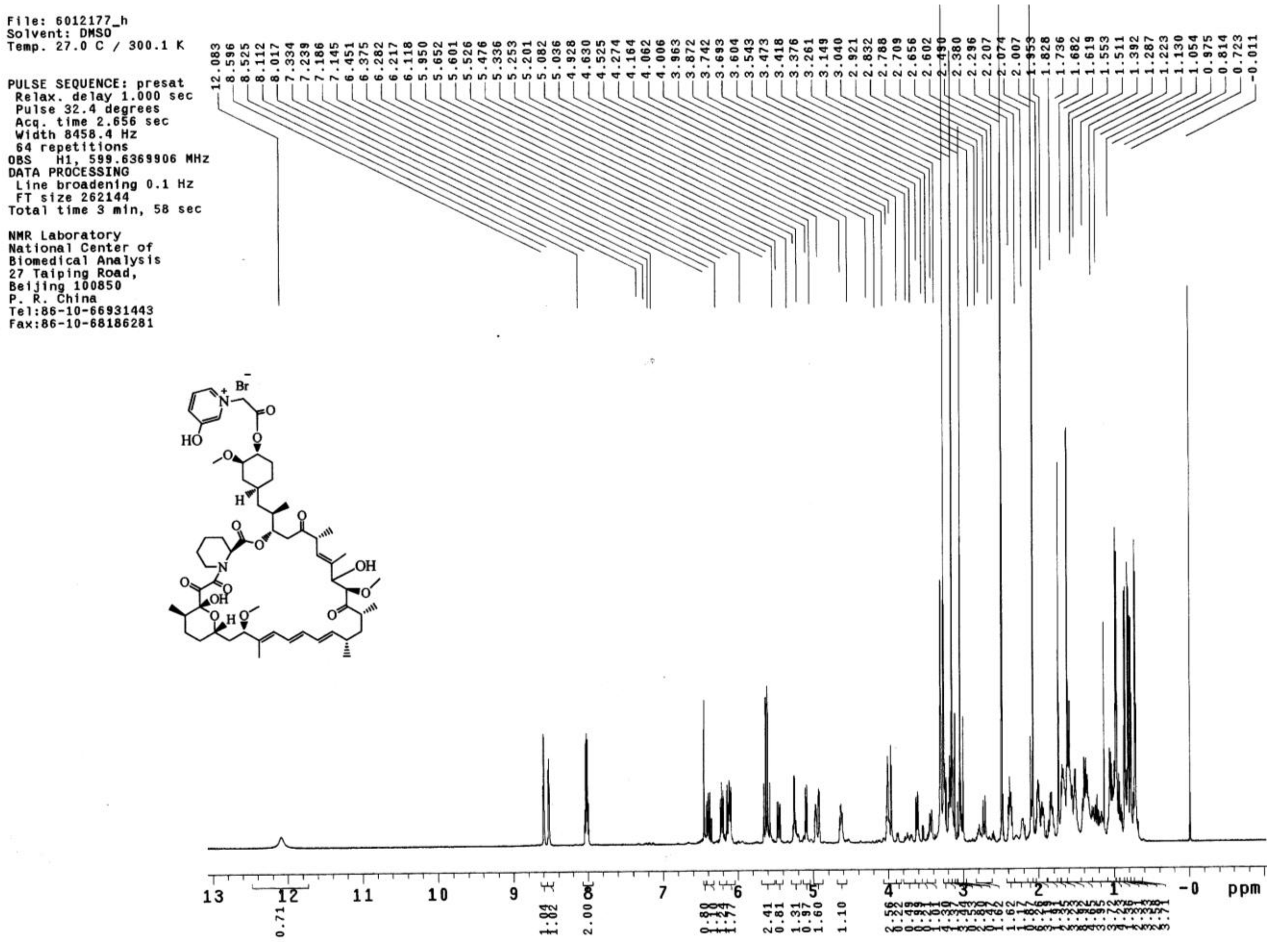

Figure S26. ^{13}C-NMR of compound **5**.

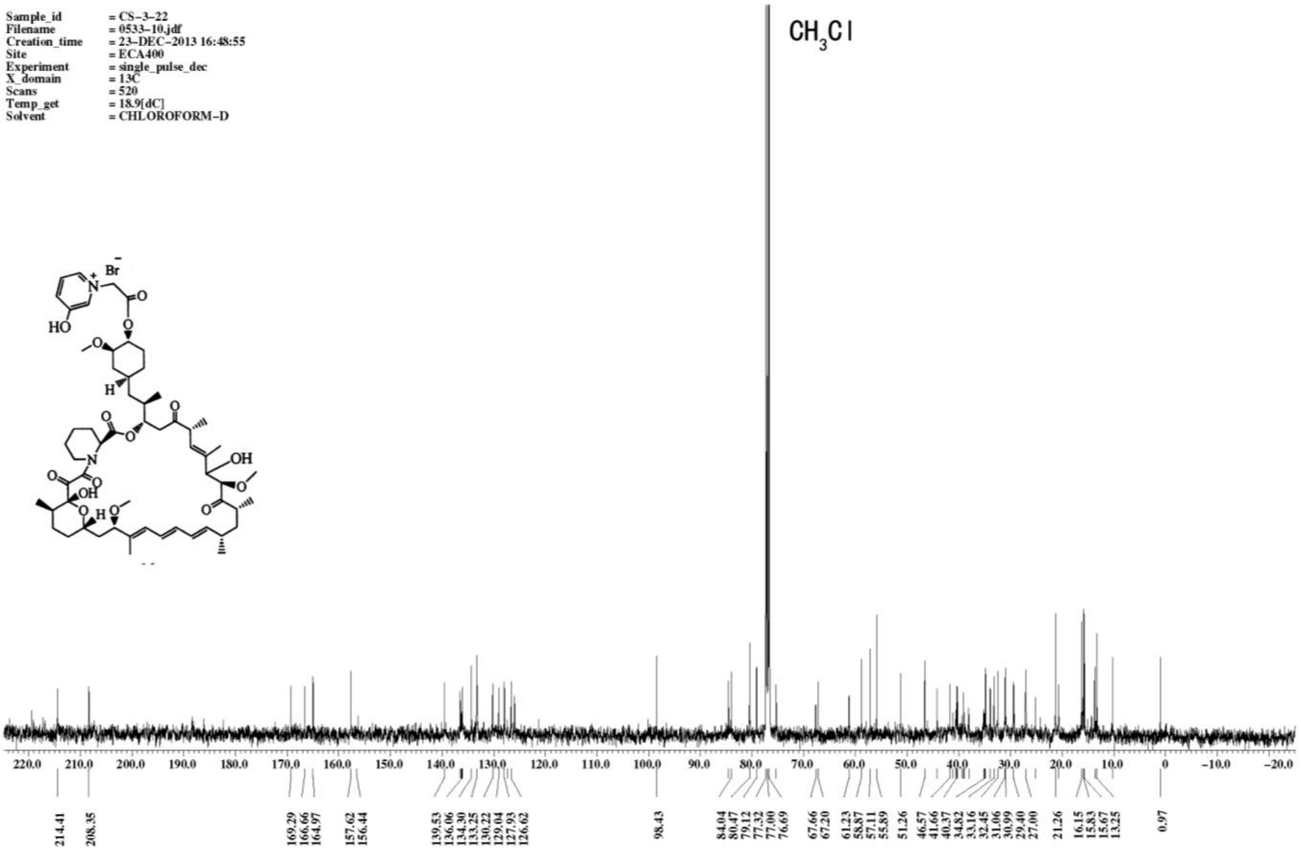

Figure S27. MS of compound **5**.

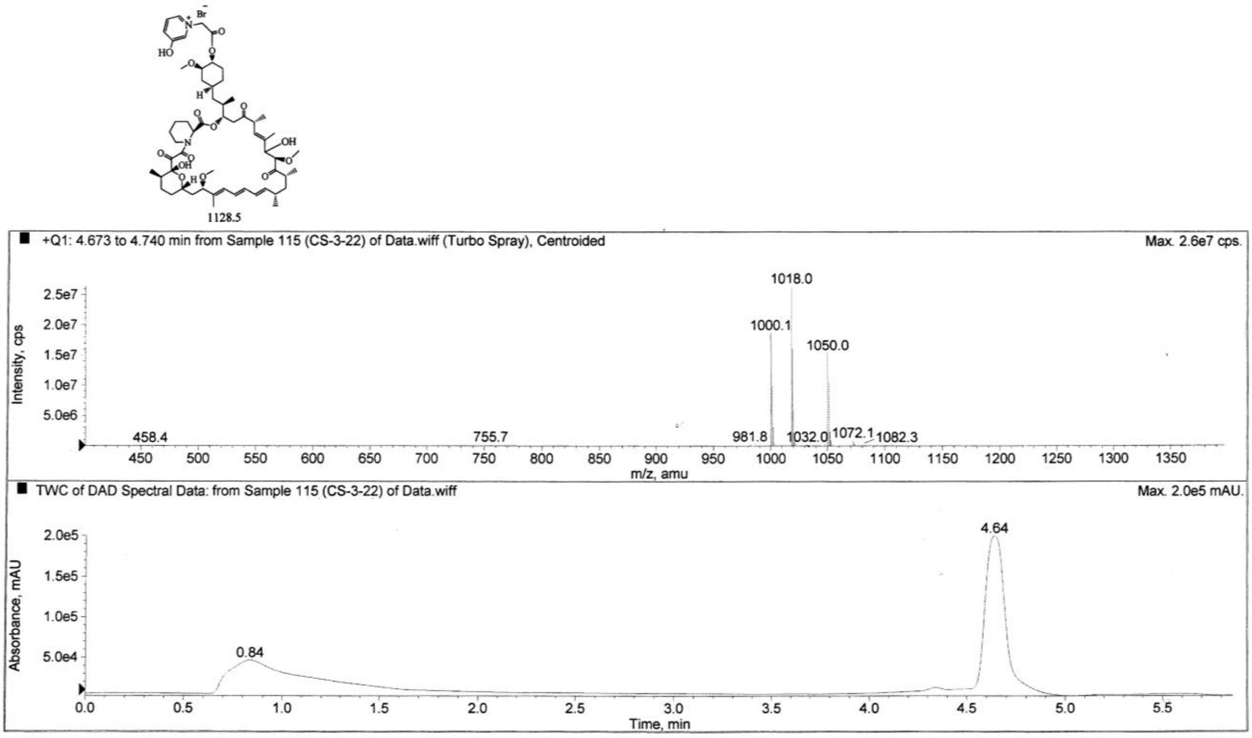

Figure S28. IR of compound **5**.

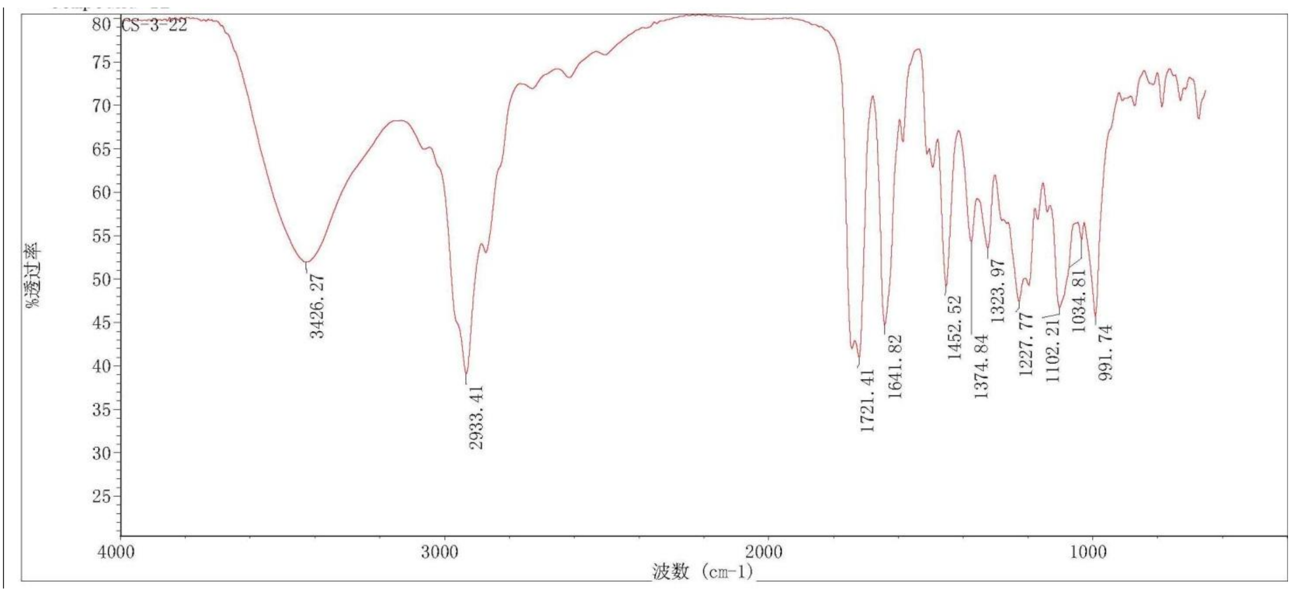

Figure S29. ^{1}H-NMR of compound **6**.

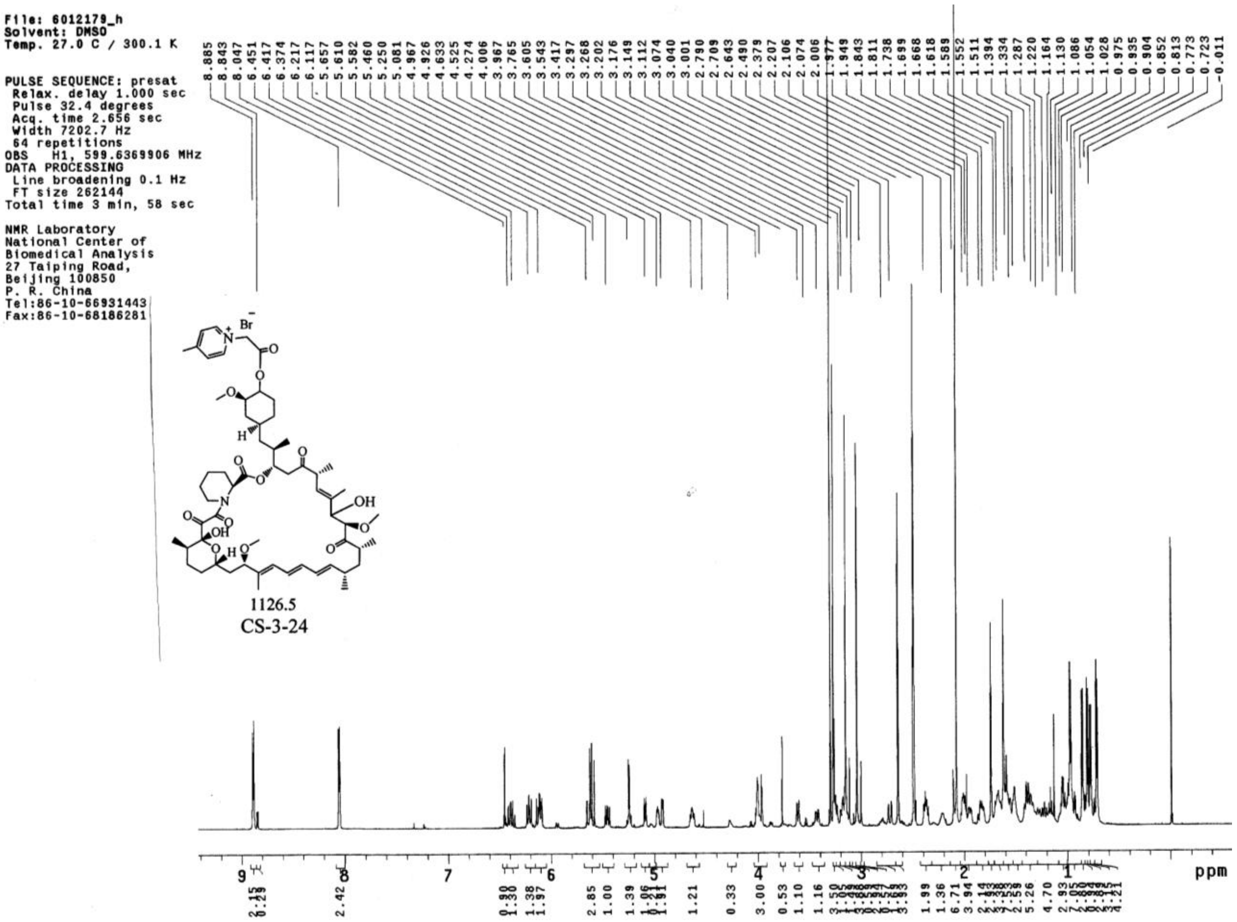

Figure S30. ^{13}C-NMR of compound **6**.

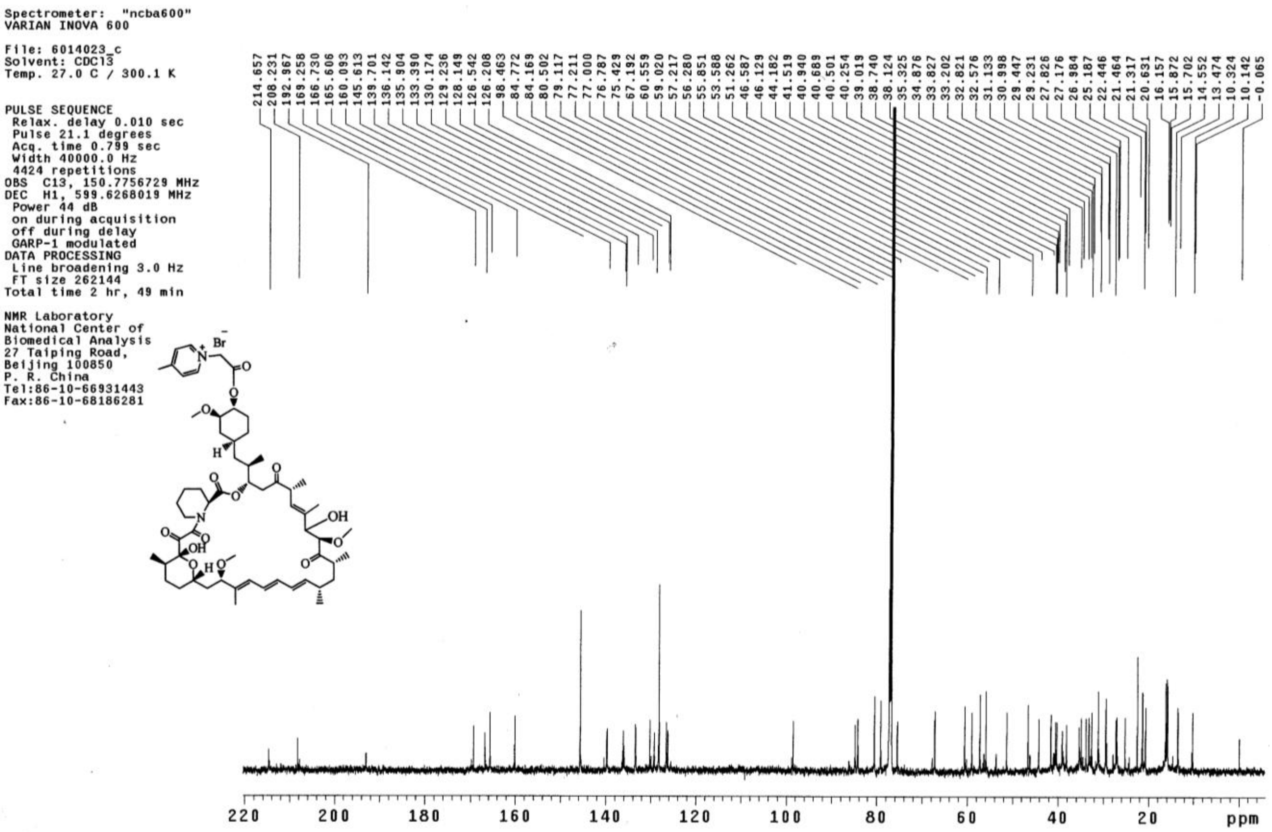

Figure S31. MS of compound **6**.

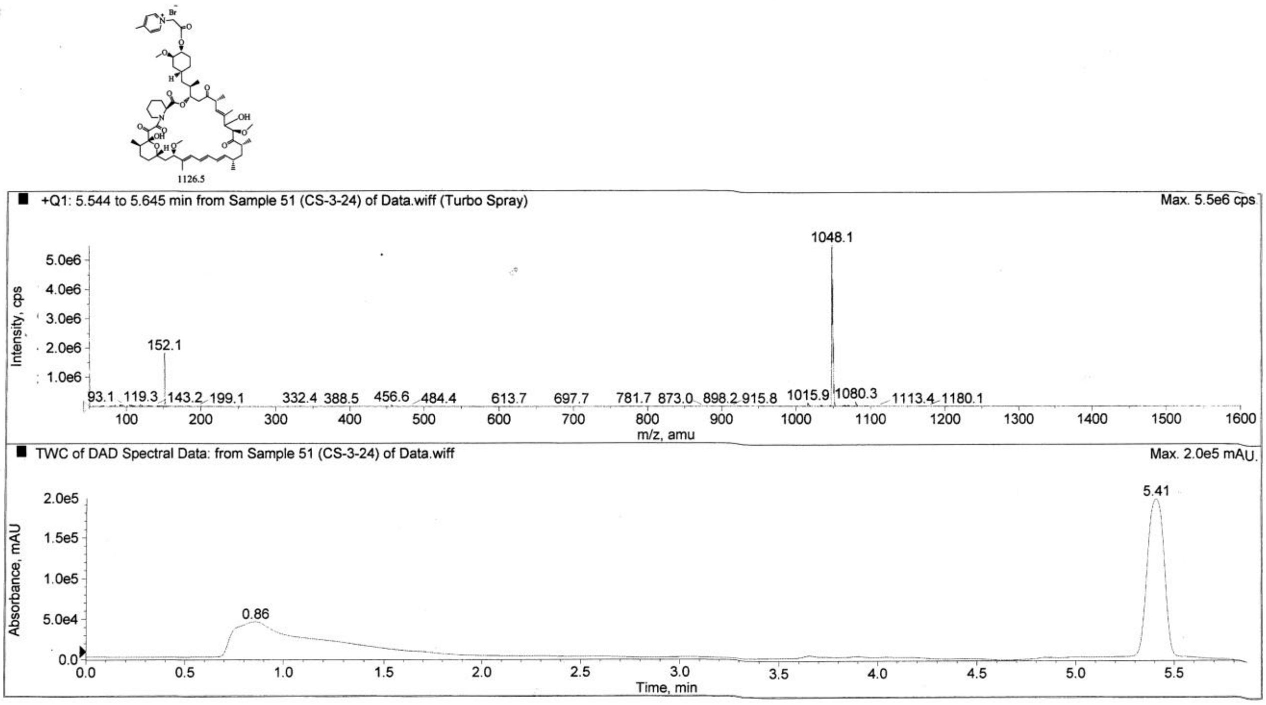

Figure S32. IR of compound **6**.

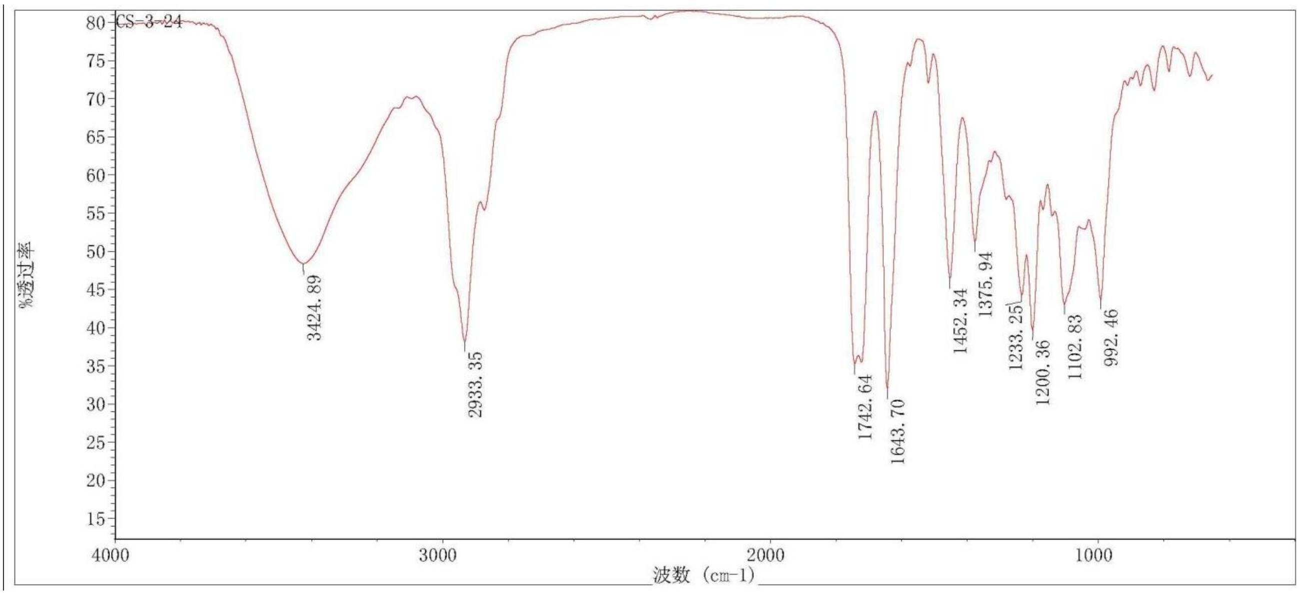

Figure S33. ^{1}H-NMR of compound **7**.

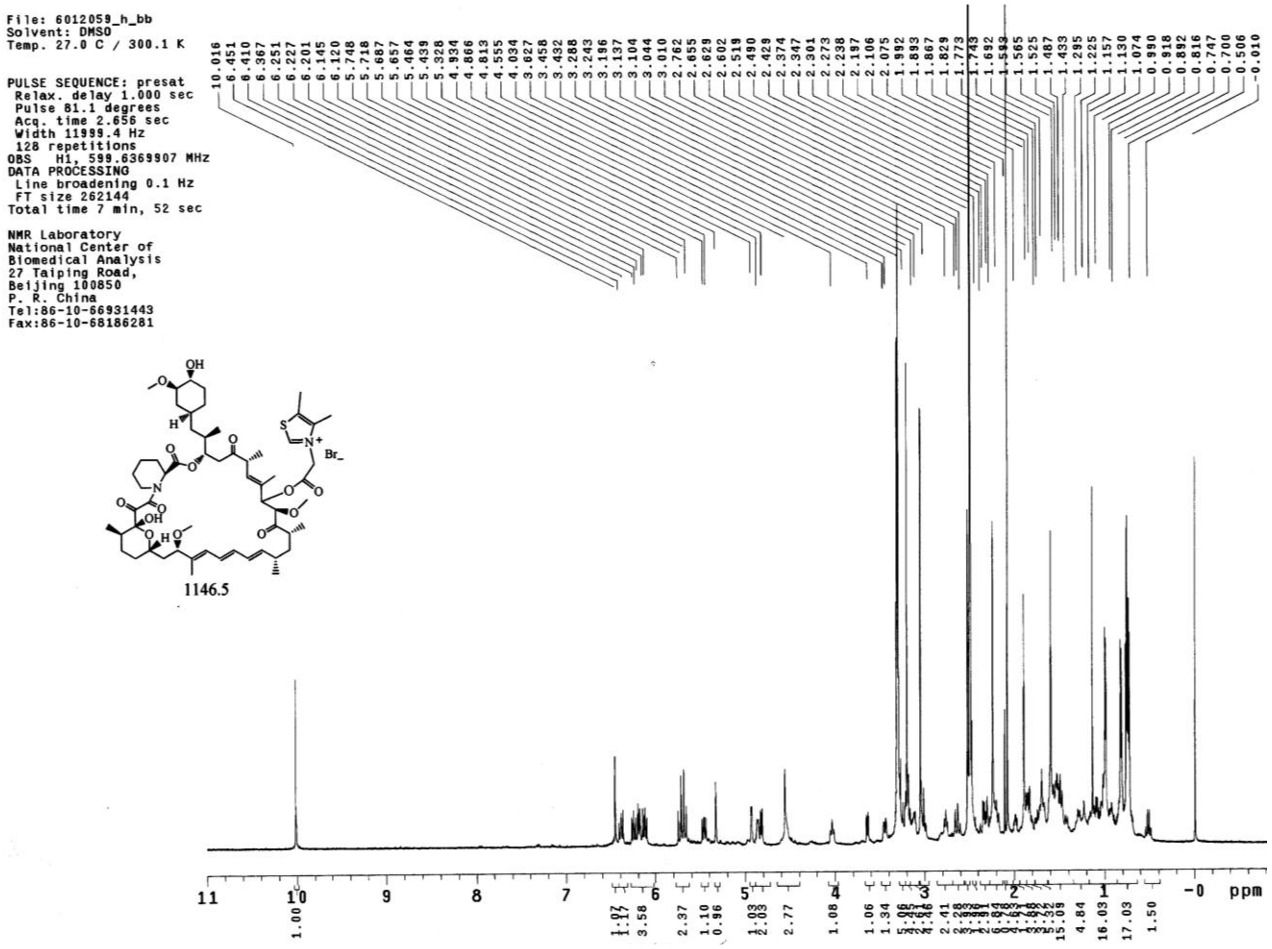

Figure S34. ^{13}C-NMR of compound **7**.

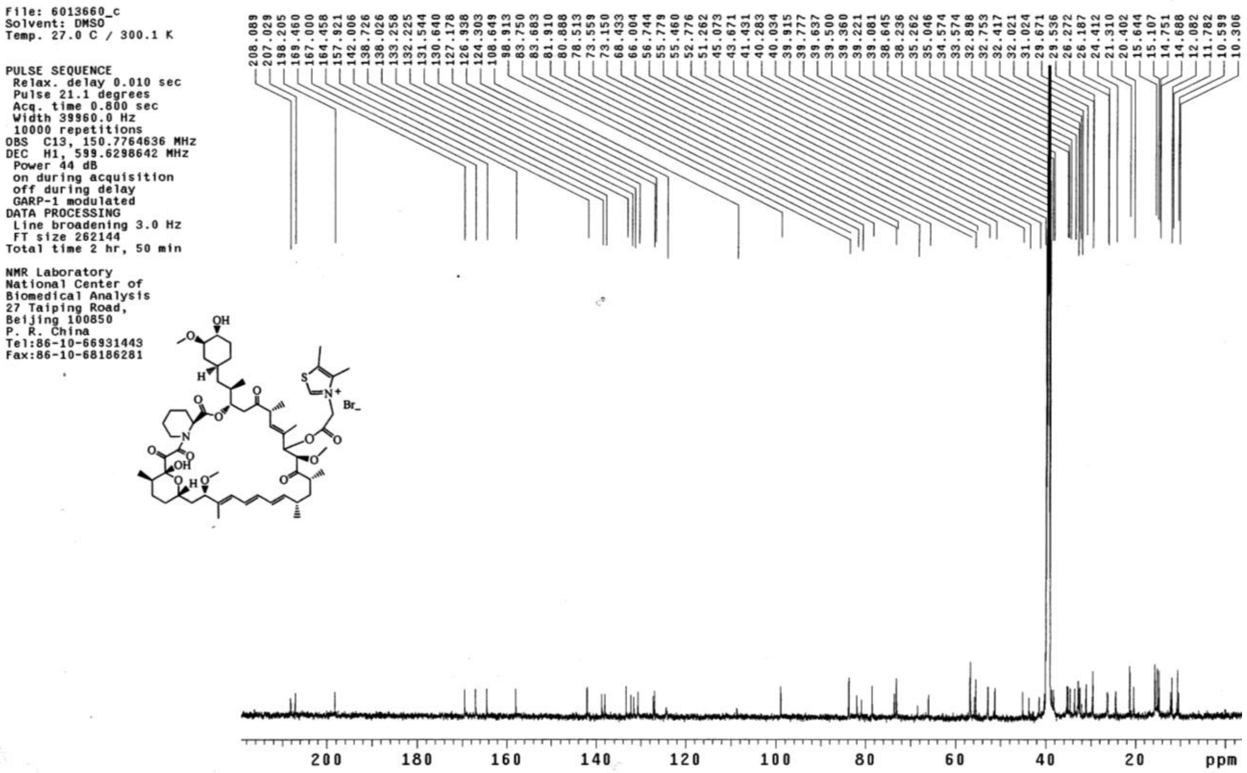

Figure S35. HSQC of compound **7**.

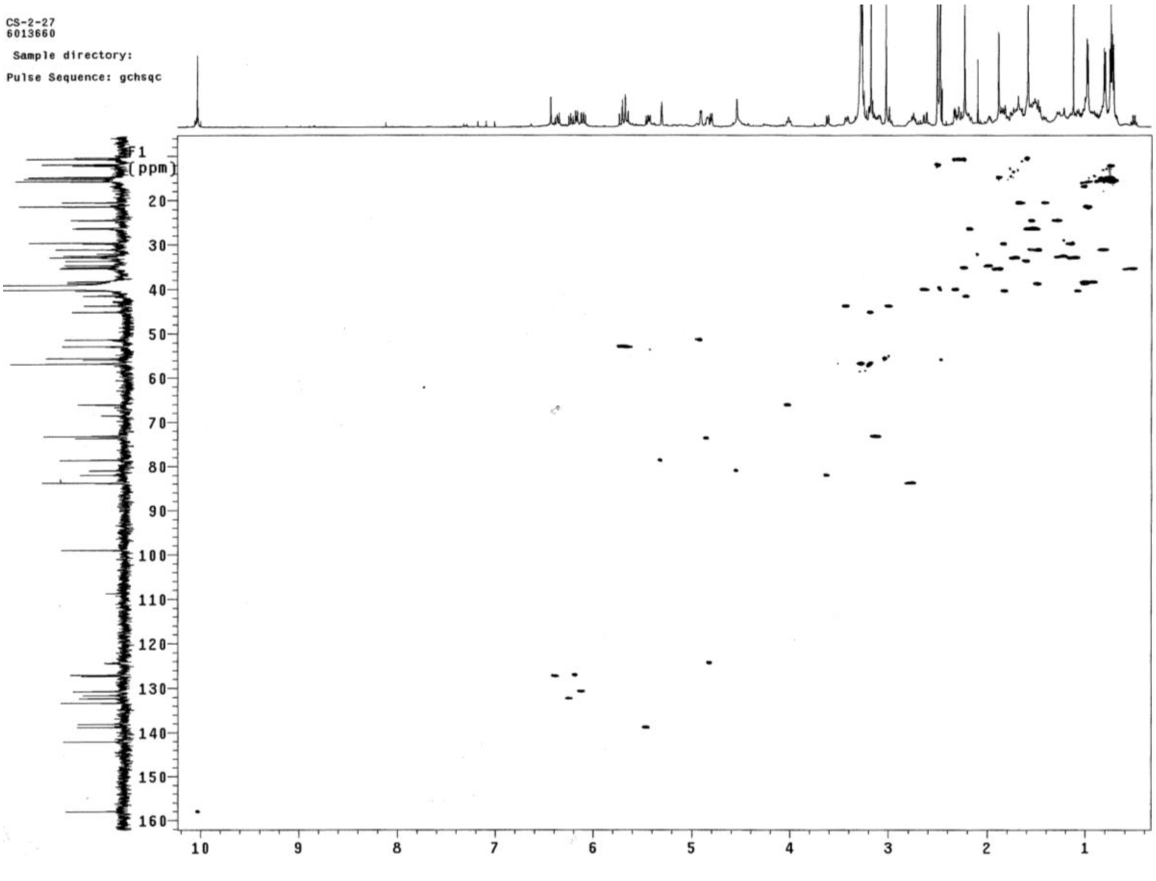

Figure S36. HMBC of compound **7**.

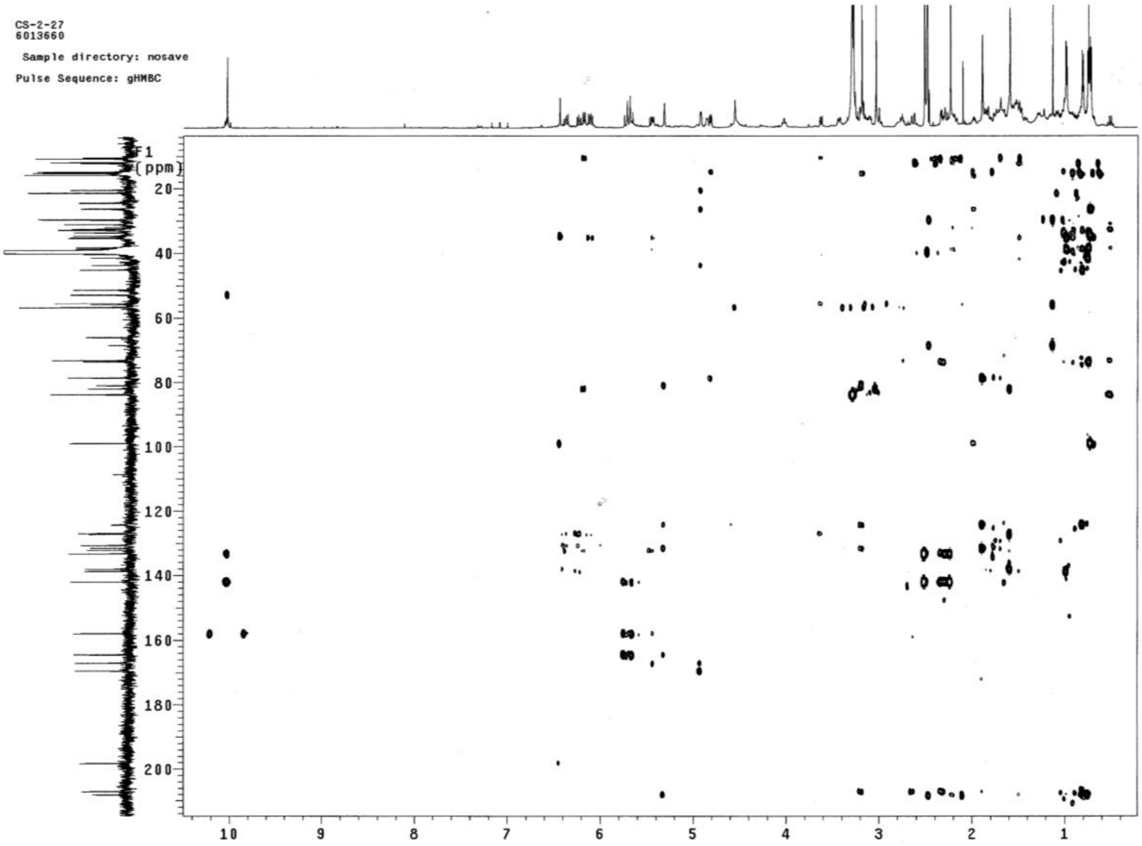

Figure S37. COSY of compound **7**.

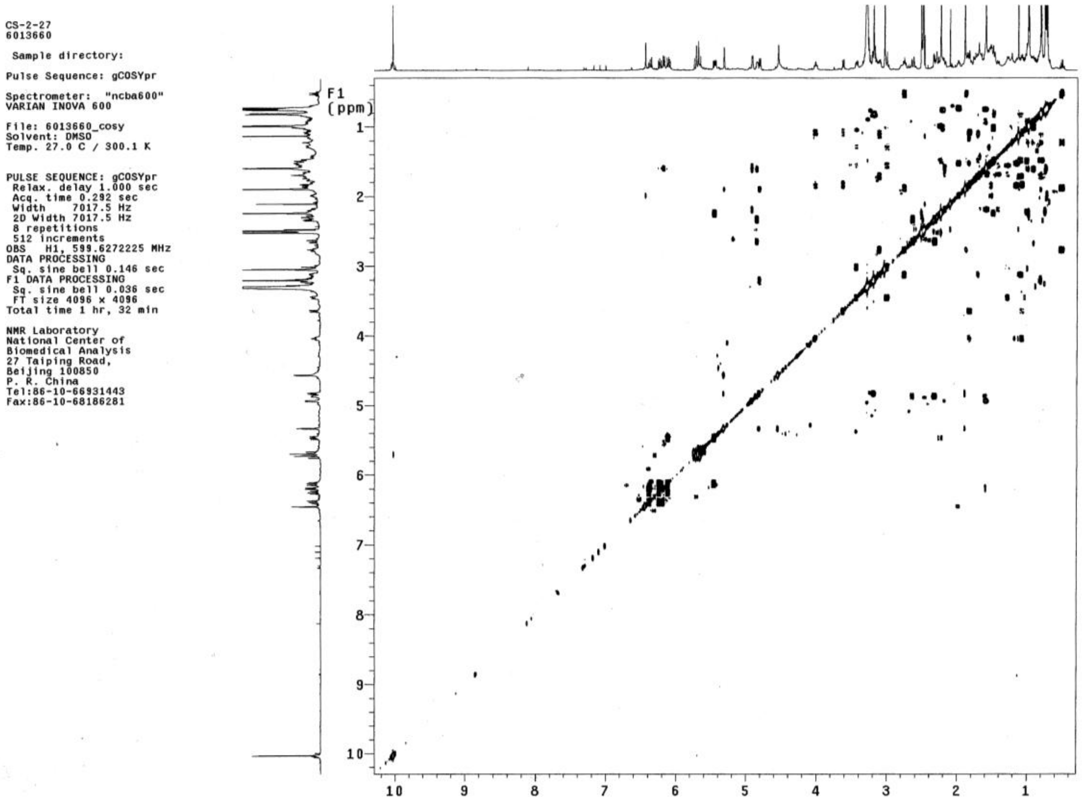

Figure S38. ROESY of compound **7**.

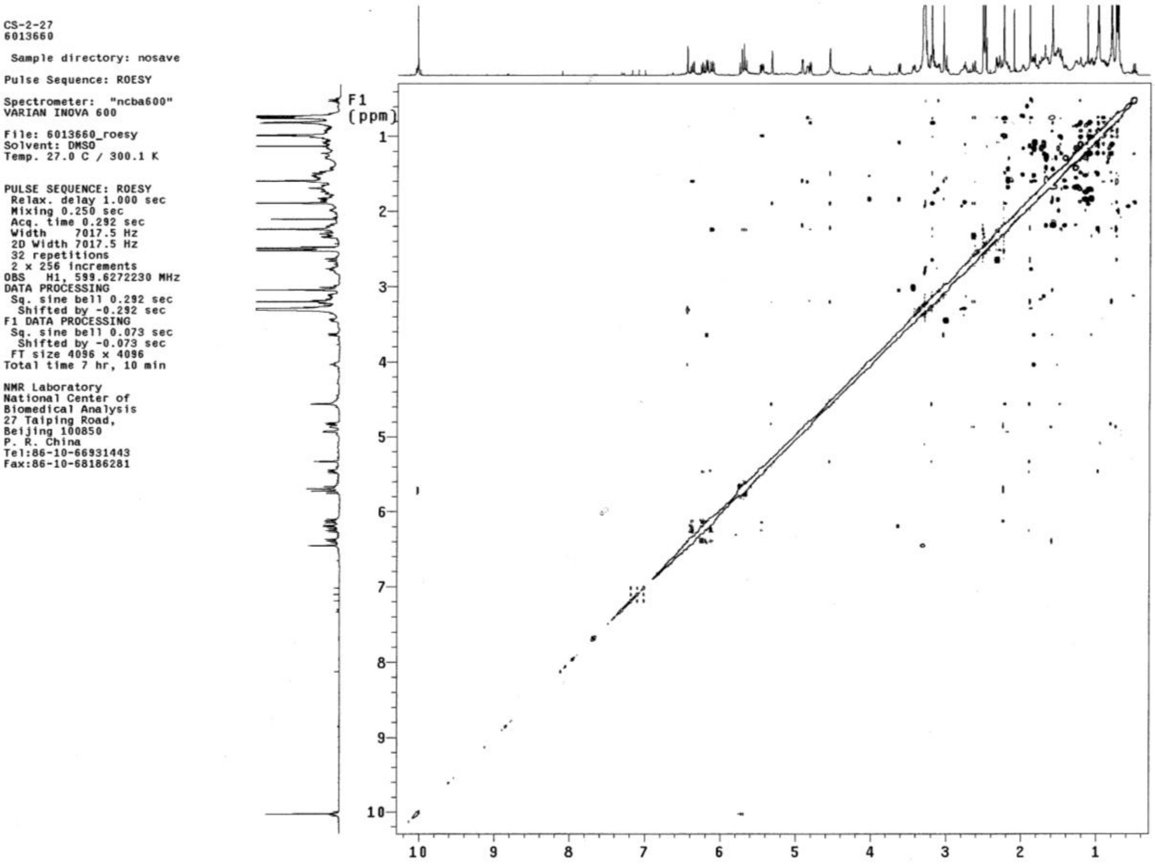

Figure S39. TOCSY of compound **7**.

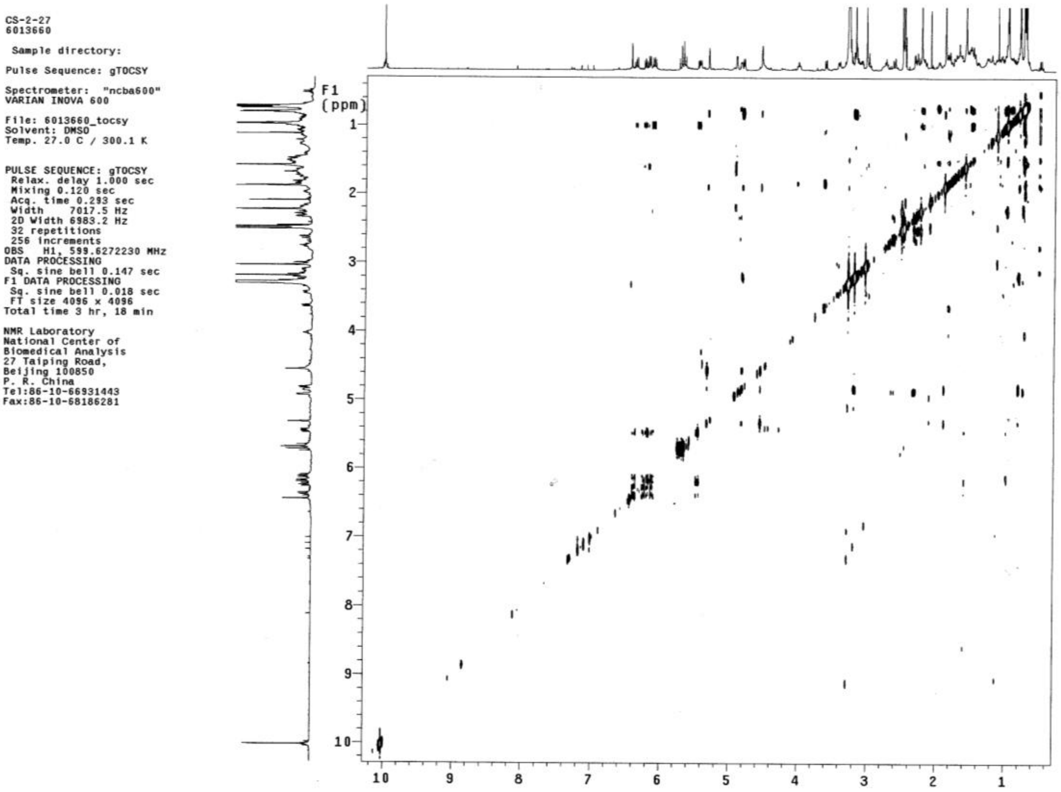

Figure S40. MS of compound **7**.

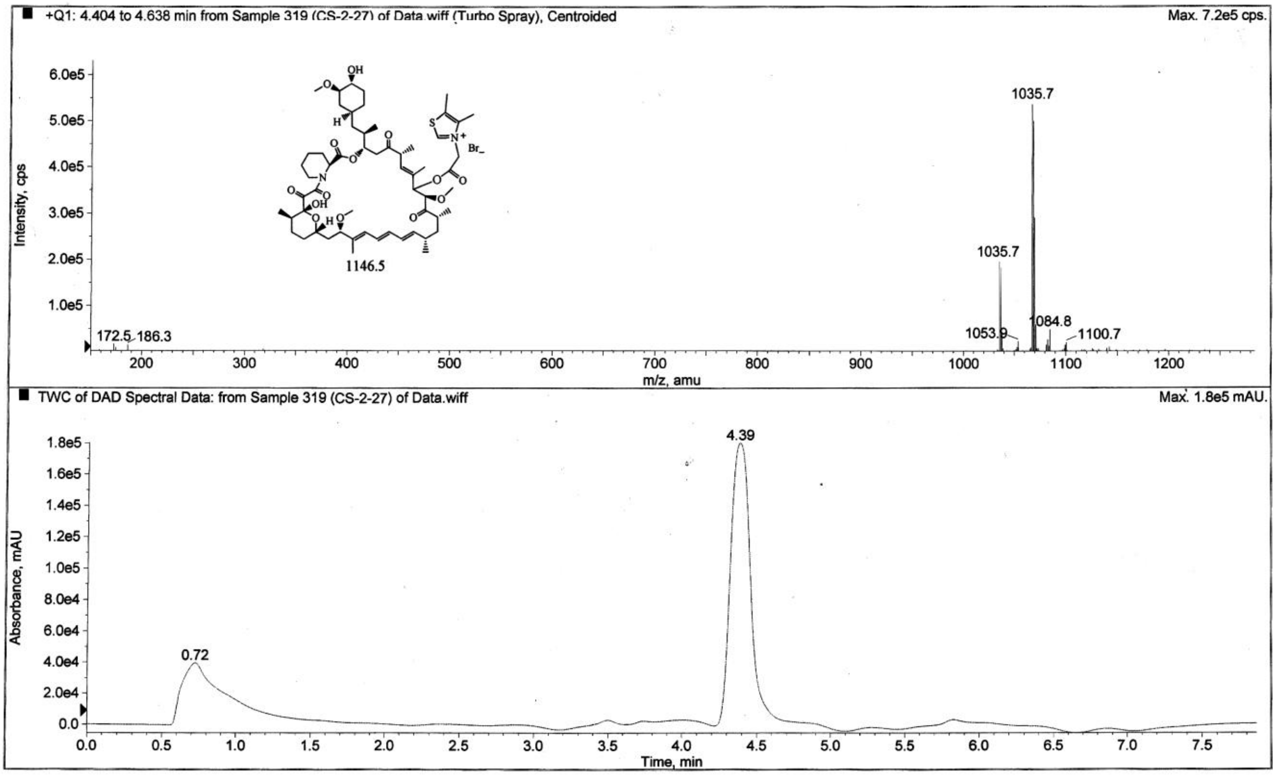

Figure S41. IR of compound **7**.

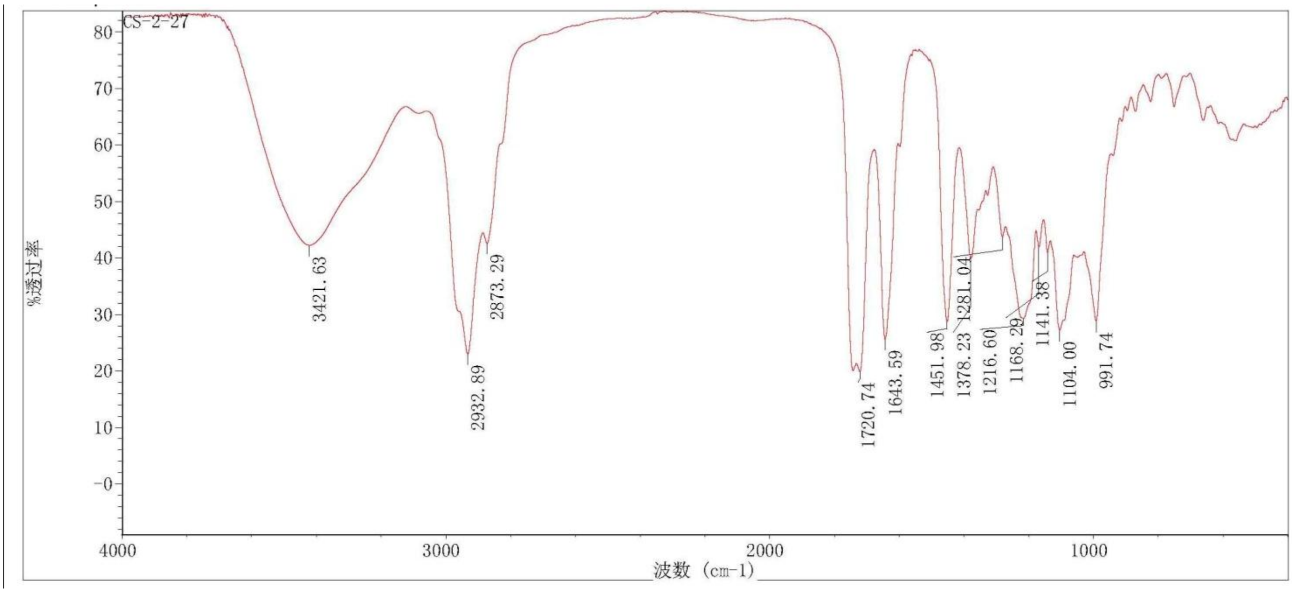

Figure S42. ^{1}H-NMR of compound **8**.

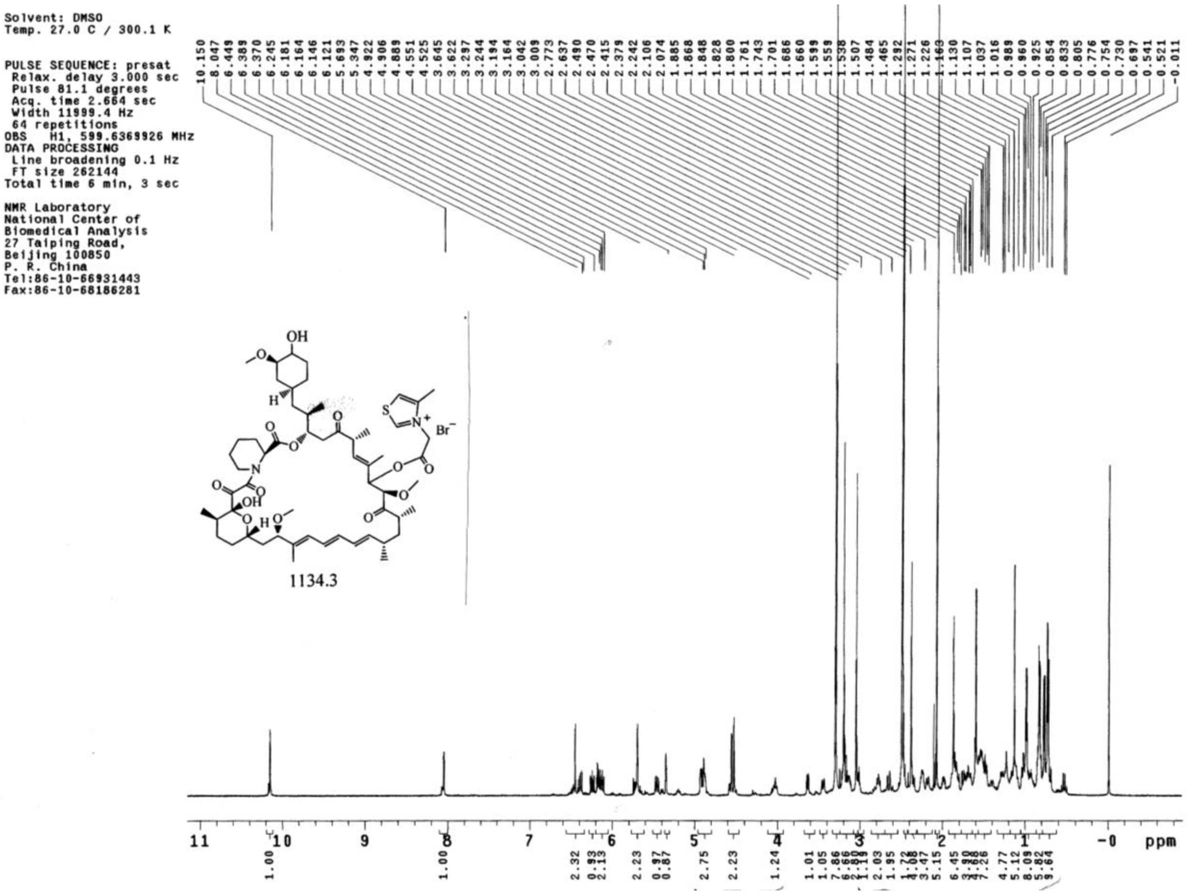

Figure S43. ^{13}C-NMR of compound **8**.

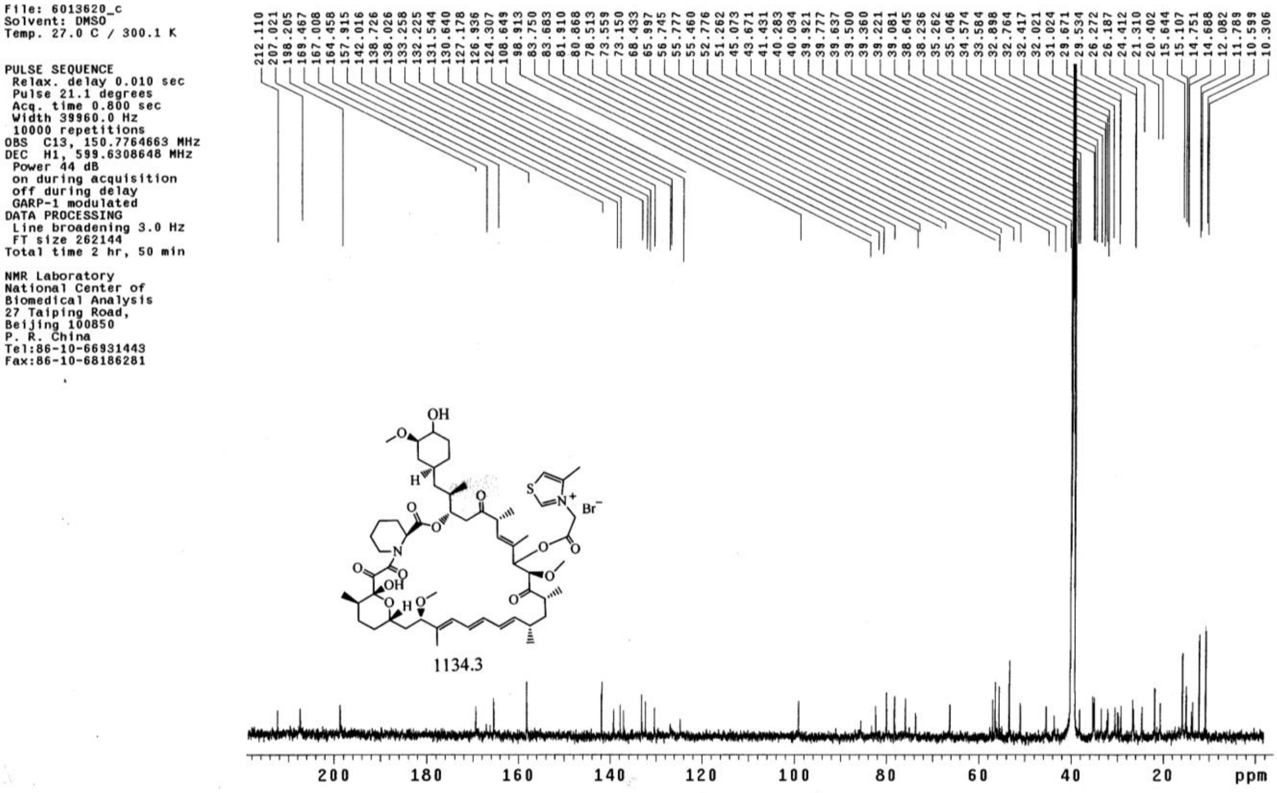

Figure S44. MS of compound **8**.

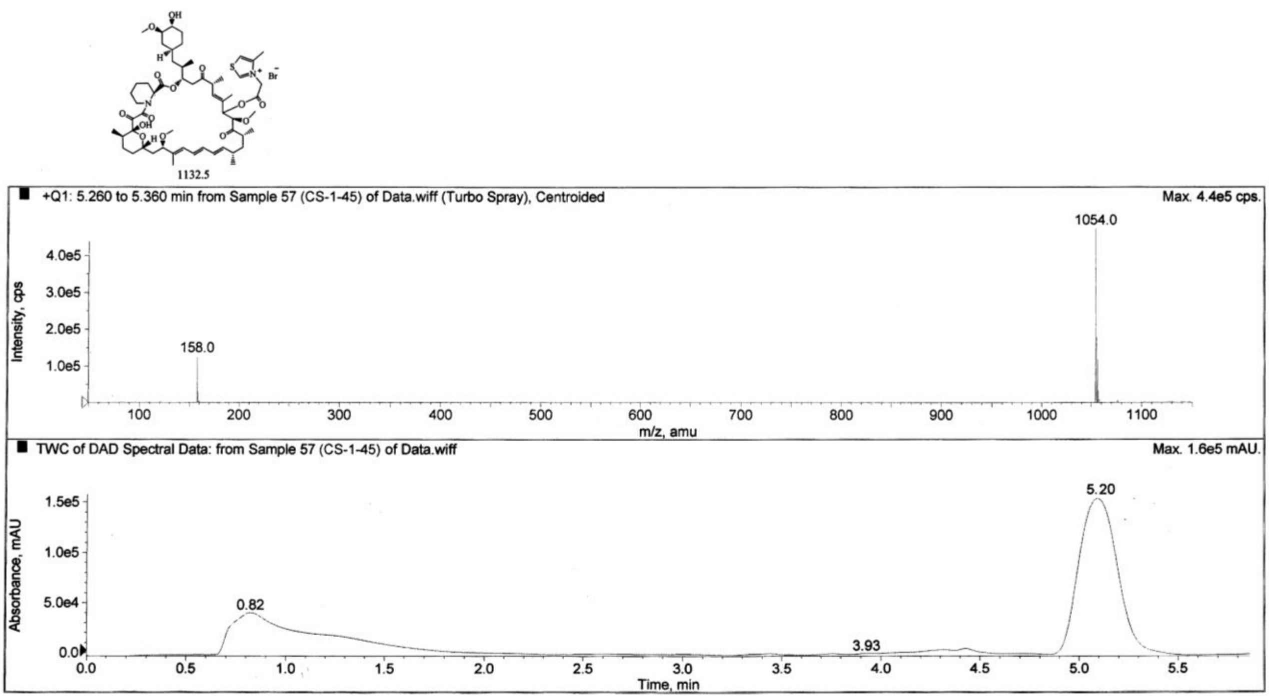

Figure S45. IR of compound **8**.

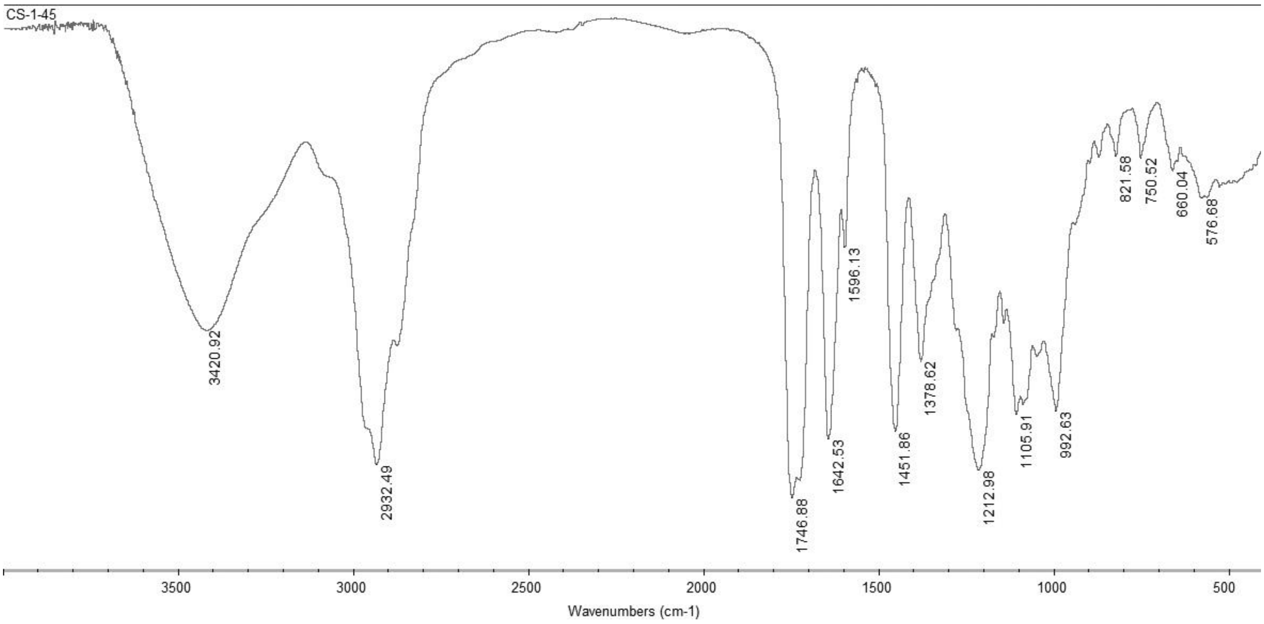

Figure S46. ^{1}H-NMR of compound **9**.

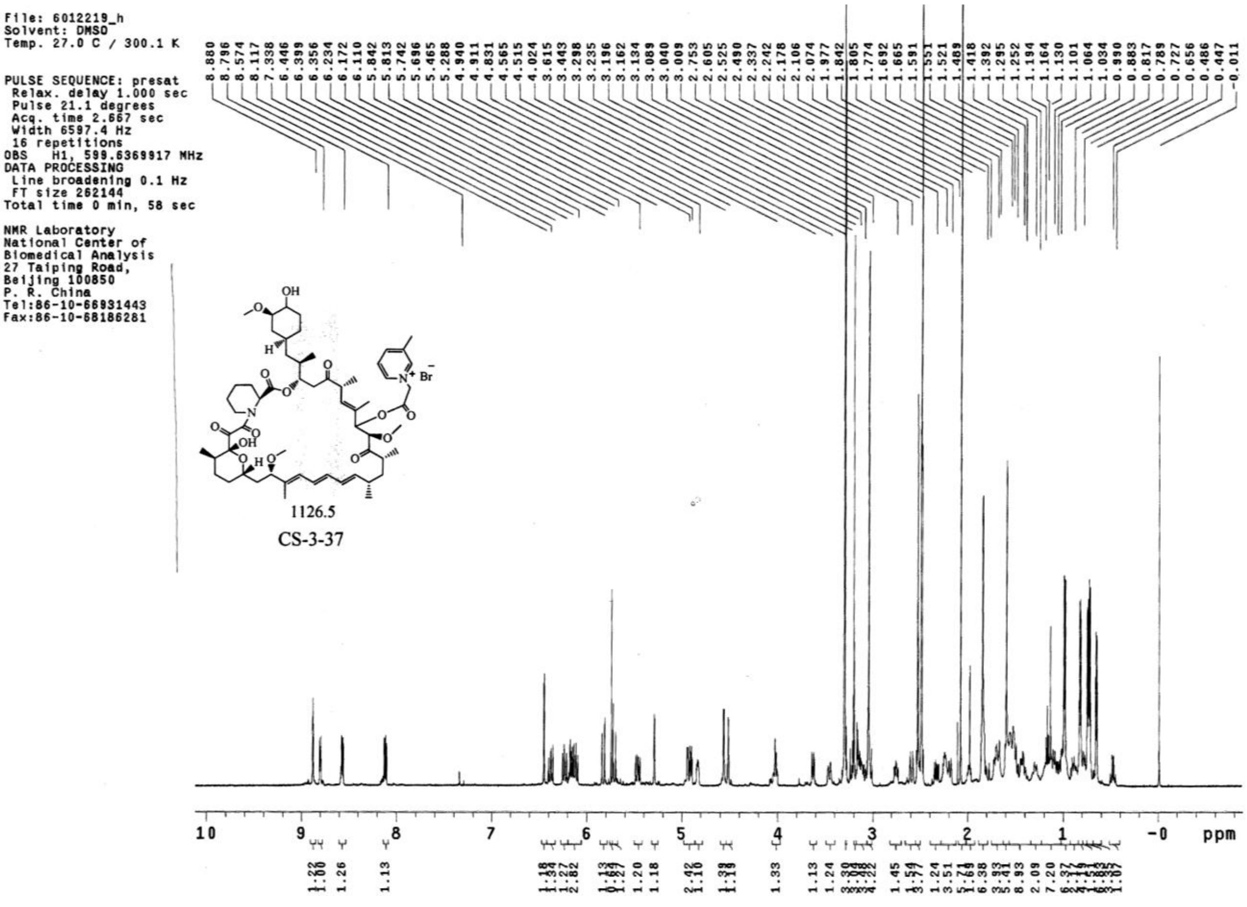

Figure S47. ^{13}C-NMR of compound **9**.

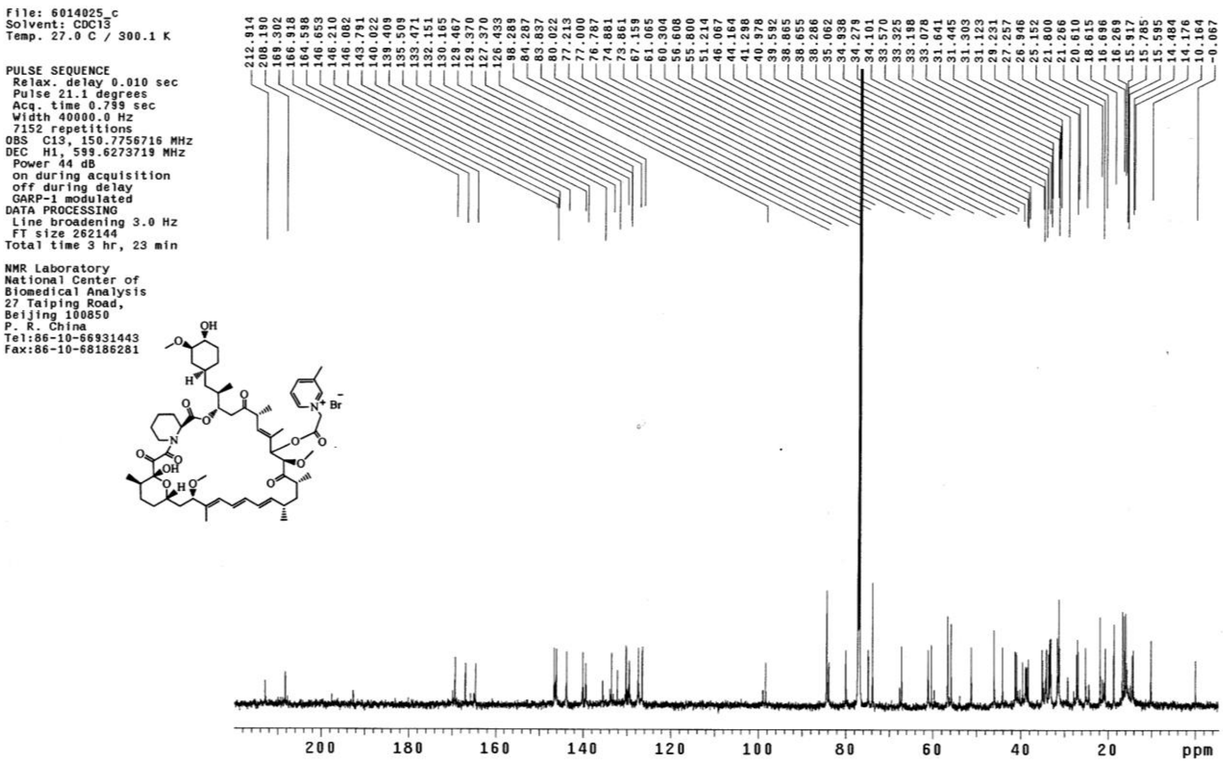

Figure S48. MS of compound **9**.

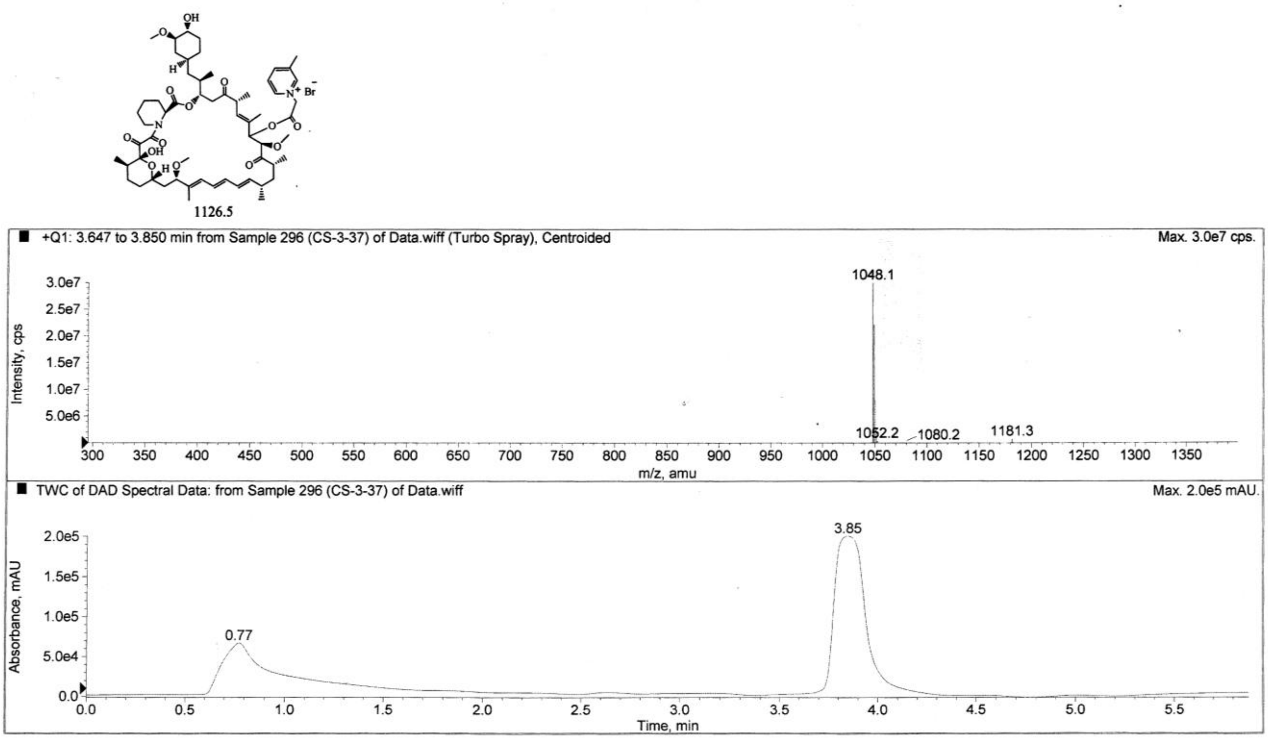

Figure S49. IR of compound **9**.

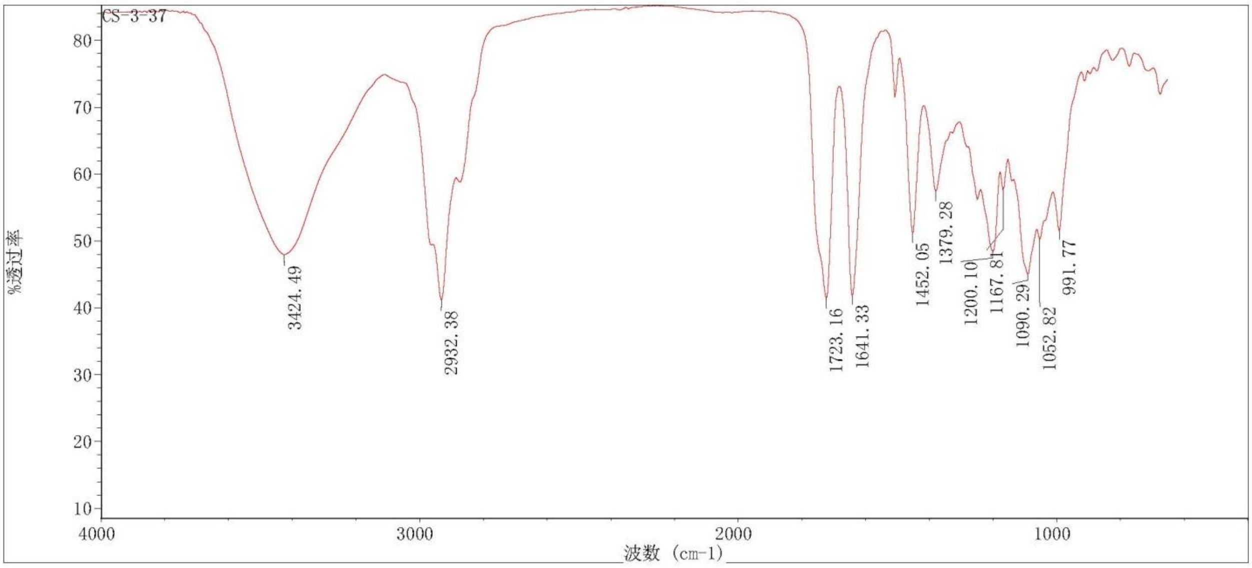

Figure S50. ^{1}H-NMR of compound **10**.

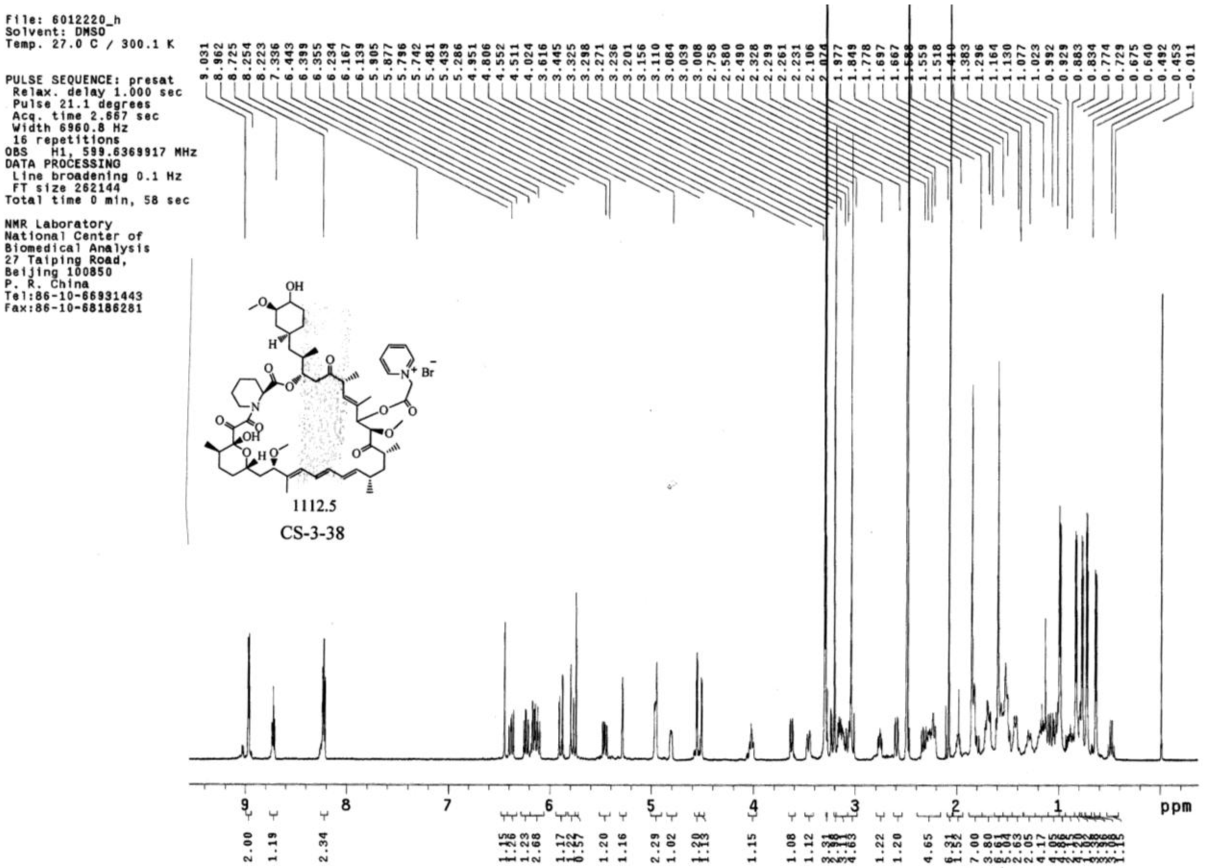

Figure S51. ^{13}C-NMR of compound **10**.

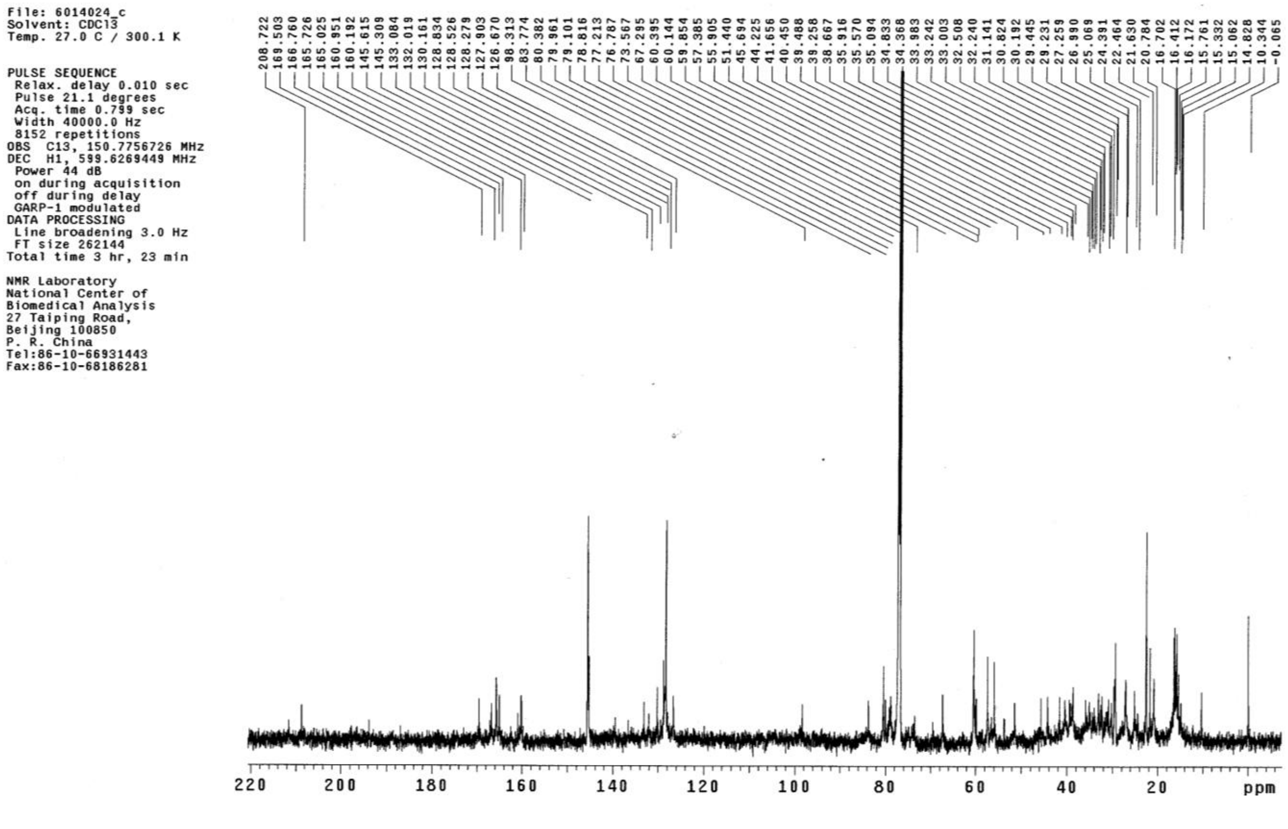

Figure S52. MS of compound **10**.

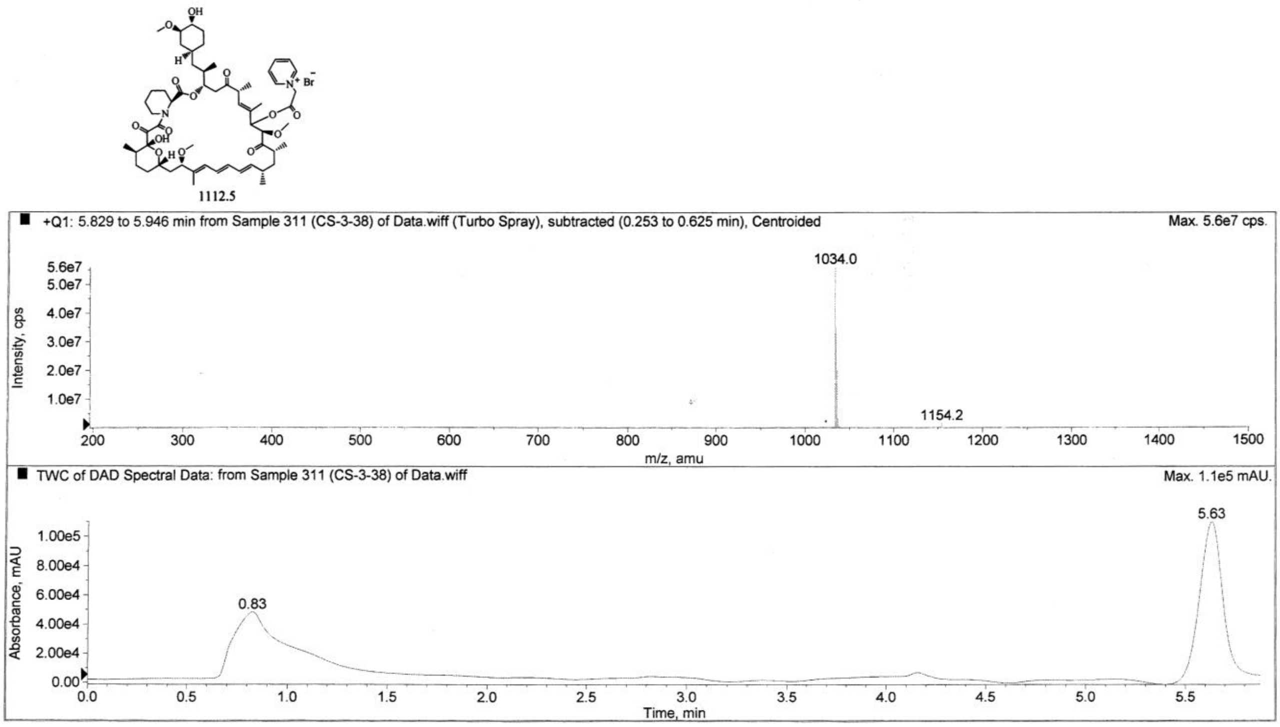

Figure S53. IR of compound **10**.

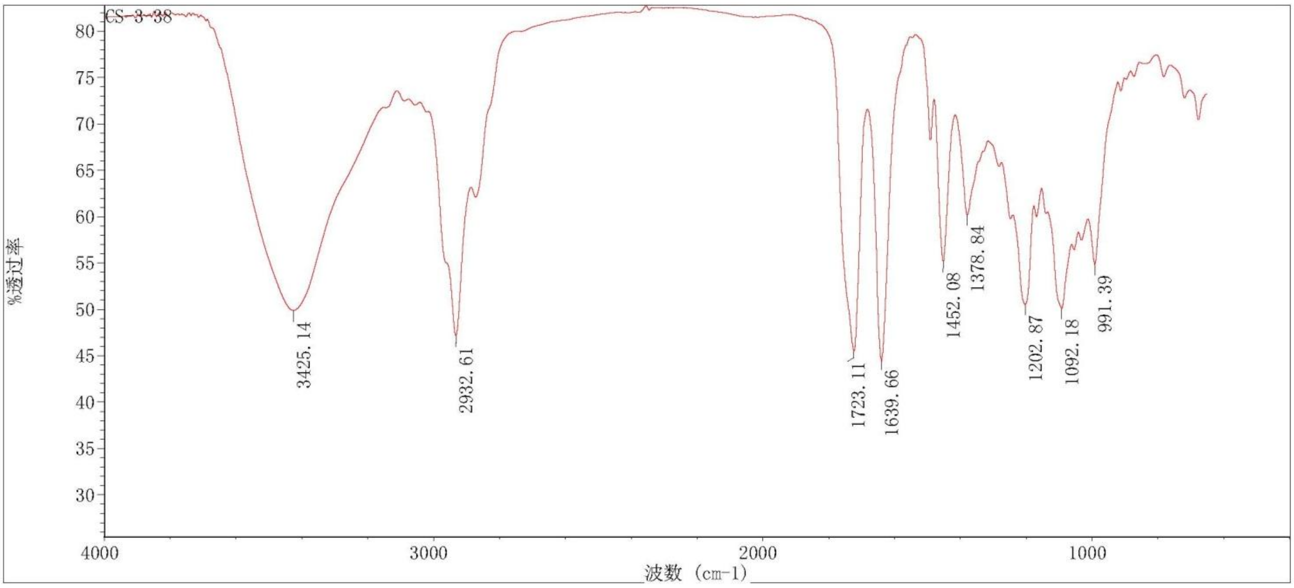

Figure S54. ^{1}H-NMR of compound **11**.

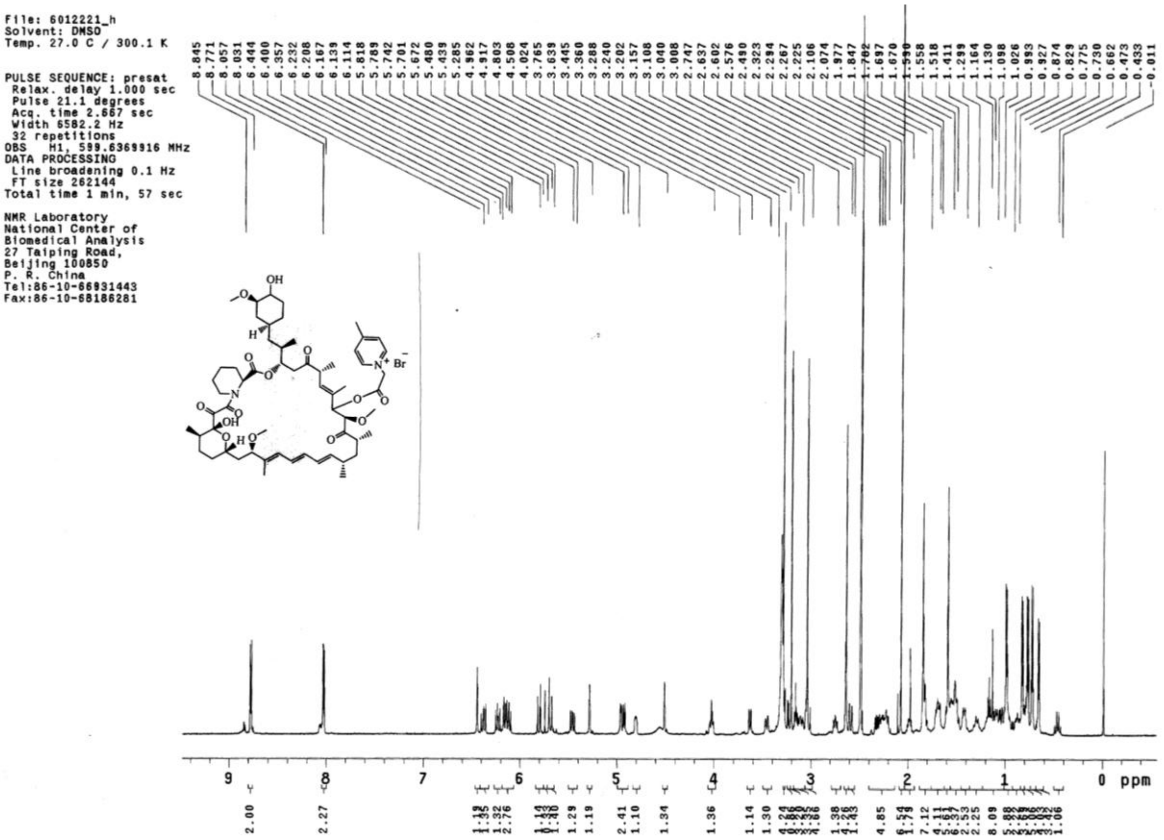

Figure S55. ^{13}C-NMR of compound **11**.

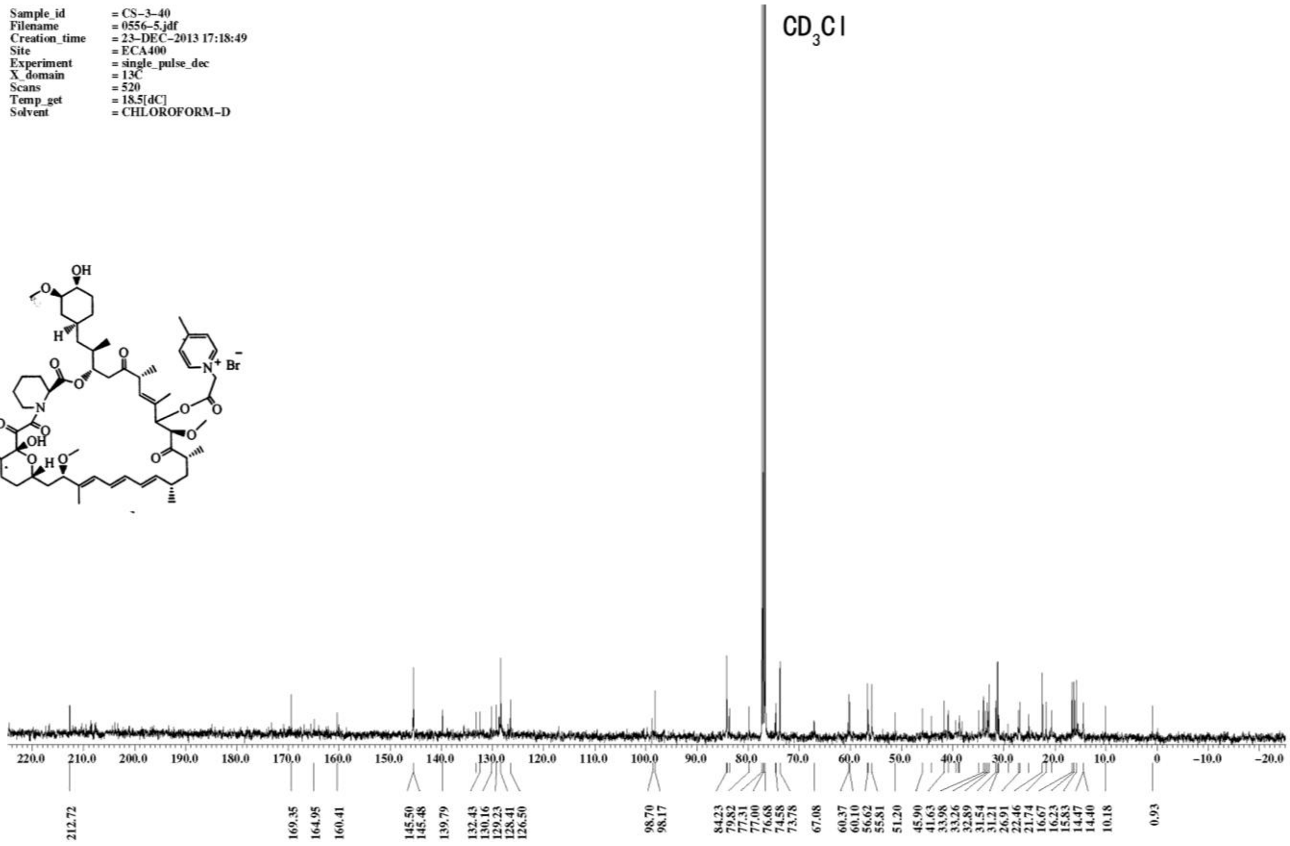

Figure S56. MS of compound **11**.

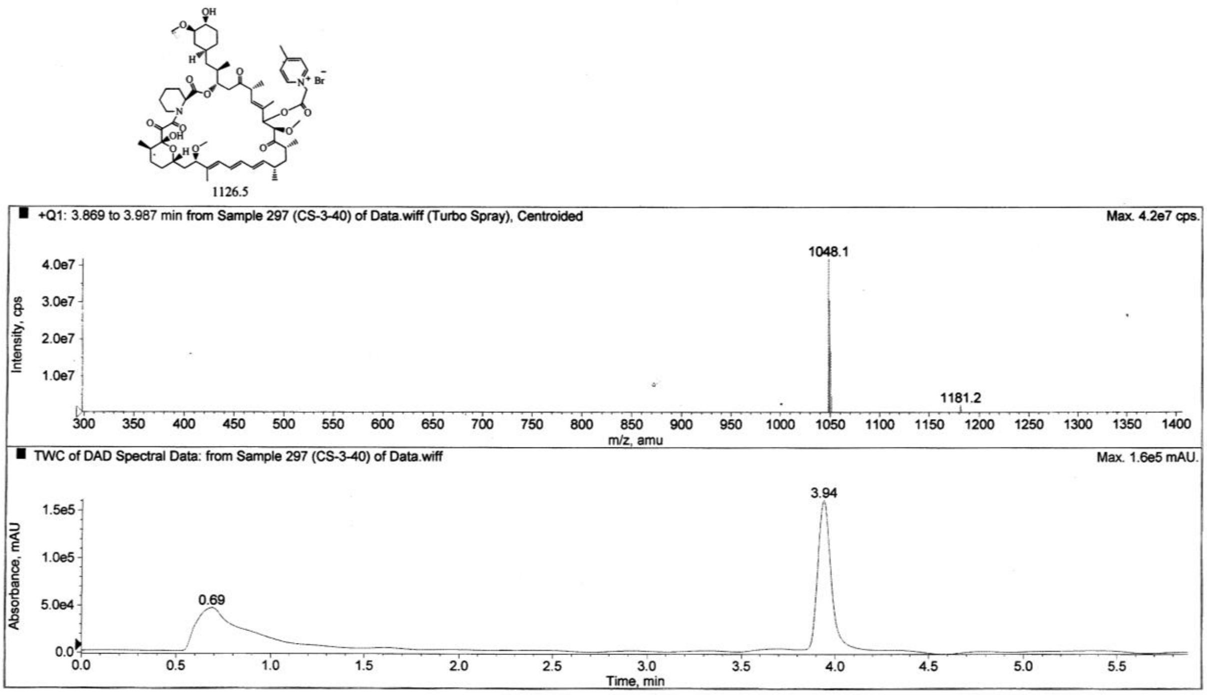

Figure S57. IR of compound **11**.

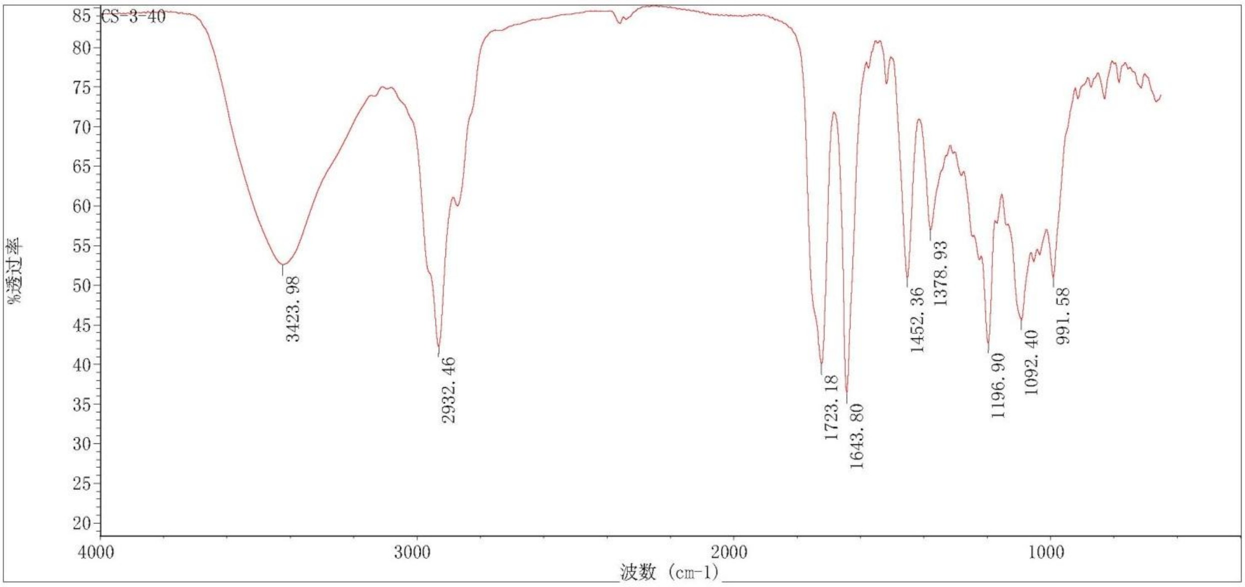

Supplement: Supplementary file 1 [file molecules-19-07770-s001.pdf]
